# DNA Metabarcoding Authentication of Ayurvedic Herbal Products on the European Market Raises Concerns of Quality and Fidelity

**DOI:** 10.3389/fpls.2019.00068

**Published:** 2019-02-05

**Authors:** Gopalakrishnan Saroja Seethapathy, Ancuta-Cristina Raclariu-Manolica, Jarl Andreas Anmarkrud, Helle Wangensteen, Hugo J. de Boer

**Affiliations:** ^1^Natural History Museum, University of Oslo, Oslo, Norway; ^2^Department of Pharmaceutical Chemistry, School of Pharmacy, University of Oslo, Oslo, Norway; ^3^Stejarul Research Centre for Biological Sciences, National Institute of Research and Development for Biological Sciences, Piatra Neamt, Romania

**Keywords:** Ayurvedic herbal products, botanical authentication, DNA barcoding, herbal medicines, pharmacovigilance, quality control

## Abstract

Ayurveda is one of the oldest systems of medicine in the world, but the growing commercial interest in Ayurveda based products has increased the incentive for adulteration and substitution within this herbal market. Fraudulent practices such as the use of undeclared fillers and use of other species of inferior quality is driven both by the increased as well as insufficient supply capacity of especially wild plant species. Developing novel strategies to exhaustively assess and monitor both the quality of raw materials and final marketed herbal products is a challenge in herbal pharmacovigilance. Seventy-nine Ayurvedic herbal products sold as tablets, capsules, powders, and extracts were randomly purchased via e-commerce and pharmacies across Europe, and DNA metabarcoding was used to assess the ability of this method to authenticate these products. Our analysis reveals that only two out of 12 single ingredient products contained only one species as labeled, eight out of 27 multiple ingredient products contained none of the species listed on the label, and the remaining 19 products contained 1 to 5 of the species listed on the label along with many other species not specified on the label. The fidelity for single ingredient products was 67%, the overall ingredient fidelity for multi ingredient products was 21%, and for all products 24%. The low level of fidelity raises concerns about the reliability of the products, and detection of threatened species raises further concerns about illegal plant trade. The study highlights the necessity for quality control of the marketed herbal products and shows that DNA metabarcoding is an effective analytical approach to authenticate complex multi ingredient herbal products. However, effort needs to be done to standardize the protocols for DNA metabarcoding before this approach can be implemented as routine analytical approaches for plant identification, and approved for use in regulated procedures.

## Introduction

Ayurveda, or Ayurvedic medicine, is one of the oldest systems of traditional medicine (TM), with origins in India more than 3,000 years ago. Nowadays Ayurveda is popular and used worldwide in complementary and alternative healthcare and medical practices (CAM) (World Health Organization [WHO], 2013). Ayurvedic formulations are obtained using an average of 80% botanicals, 12% animals, and 8% minerals, and are used as raw materials and preparations such as extracts (Joshi et al., 2017). About 7,000 plant species are used for medicinal purposes in India, from which, about 1,200 species have been reported to be actively traded (Goraya and Ved, 2017). The total commercial demand for herbal material in India, in 2014 and 2015, was estimated to be in excess of 512,000 tons, with a market value of 1 billion USD (Goraya and Ved, 2017). India has more than 8,000 licensed manufacturing units for medicinal products and the increasing level of consumption of herbal products exceed the supply capacity for some plant species (Goraya and Ved, 2017). In order to ensure a level of uniformity of the therapeutic formula and the ingredients used, the Ayurvedic formulary and Ayurvedic Pharmacopeia of India was published by the Government of India as a legally binding document describing the quality, purity, and strength of selected drugs that are manufactured, distributed and sold by the licensed manufacturers in India (Joshi et al., 2017).

As many other TMs, Ayurvedic herbal medicines, require quality assurances for their wider usage and acceptability in CAM practicing countries (World Health Organization [WHO], 2013). The growing demand for Ayurveda encourages an industry for mass production of herbal products, leading to the use of large quantities of plant raw material, mainly harvested from the wild flora (Valiathan, 2006; Goraya and Ved, 2017; Joshi et al., 2017). Many of the Indian medicinal plant species are in short supply due to the lack of cultivation and several wild species are not available in sufficient quantities for commercial exploitation (Goraya and Ved, 2017). The intensive use of herbal products increases the incentive for adulteration and substitution in the medicinal plant trade (Newmaster et al., 2013). This awareness of content irregularities calls attention to the quality of the traded mass produced herbal products with direct impact on their efficacy and safety (Leonti and Casu, 2013). One of the pharmacognostic parameters to assure quality, safety and efficacy of a herbal medicine is the utilization of correctly identified medicinal plants used as raw material (Evans, 2009). Several new strategies and appropriate standard methods have been proposed to exhaustively assess and monitor both the quality of raw materials and marketed herbal products (Barnes, 2003; De Boer et al., 2015). Standard methods routinely used to assess herbal material, preparations and products rely on morphological characters, microscopy, and chemical fingerprinting [i.e., thin–layer chromatography’, high–performance liquid chromatography (HPLC), and gas chromatography (GC)] (De Boer et al., 2015; Parveen et al., 2016). These methods are quick and cost-effective techniques for primary qualitative analysis of raw material and derived herbal products. Alternatively, the use of more advanced methods for identification and quantification of chemical marker compounds is becoming popular [i.e., liquid chromatography (LC)–mass spectrometry (MS), GC-MS, and LC-nuclear magnetic resonance (NMR)], but requires valuable instrumentation (Jiang et al., 2010; Wang et al., 2017; Zhang et al., 2017; Raclariu et al., 2018a).

Various important issues influence the quality of Ayurvedic herbal products and they need to be carefully taken into consideration when determining the analytical method of choice for quality control. The herbal products are usually complex mixtures of plant material and/or extracts and excipients, and results of manifold processing steps. To apply only standard analytical methods may pose serious challenges to the accuracy of herbal product quality control. Furthermore, adulteration by the deliberate use or admixture of substitutes and undeclared plant fillers, fraudulent adulteration by using fillers of botanical origin or plant materials of inferior quality (Zhang et al., 2012), the addition of pharmaceuticals or other synthetic substances in order to reach an expected effect or a certain level of marker compounds (Calahan et al., 2016; Rocha et al., 2016) raises concerns about the quality and safety of the herbal products. Multiple plant species as source for botanical drug as allowed in different pharmacopeias, as well as the accidental substitutions, all raise concerns ranging from simple misleading labeling to potential serious adverse drug reactions (Ernst, 1998; Heubl, 2010; Gilbert, 2011) or poisoning due to toxic contaminants (Chan, 2003).

All the standard analytical approaches, including sensory and chemical inspection may have a good resolution in quality control by detecting the quality and quantity of specific lead or phytochemical marker compounds. However, they are generally not applicable in identifying target plant species within a complex herbal product, and show low ability to detect non-targeted plant ingredients in herbal products (De Boer et al., 2015). To overcome this limitation, DNA-based approaches have been proposed as useful analytical tools for the quality control of herbs and herbal products (Parveen et al., 2016). DNA barcoding is a cost-effective, species-level identification based upon the use of short and standardized gene regions, known as ‘barcodes’ (Hebert et al., 2003). Several reviews have corroborated the diverse applicability of DNA barcoding in the field of medicinal plant research (Techen et al., 2014; De Boer et al., 2015). Initially used as an identification tool, DNA barcoding is now applied in the industrial quality assurance context to authenticate a wide range of herbal products (De Boer et al., 2015; Parveen et al., 2016; Sgamma et al., 2017).

The combination of High-Throughput Sequencing (HTS) and DNA barcoding, known as DNA metabarcoding, enables simultaneous high-throughput multi-taxa identification by using the extracellular and/or total DNA extracted from complex samples containing DNA of different origins (Taberlet et al., 2012). Several studies have utilized this approach in identifying and authenticating medicinal plants and derived herbal products. For example, *Echinacea* species, *Hypericum perforatum*, and *Veronica officinalis* were detected in 89, 68 and 15%, respectively, of the investigated herbal products (Raclariu et al., 2017a,b, 2018b). Similarly, Ivanova et al. (2016) found that 15 tested herbal supplements contained non-listed, non-filler plant DNA, and Cheng et al. (2014) showed that the quality of 27 tested herbal preparations was highly affected by the presence of contaminants. Coghlan et al. (2012) revealed the species composition of 15 highly processed traditional Chinese medicines using DNA metabarcoding, and showed that the products contained species included on CITES appendices I and II. A number of studies in India have surveyed herbal raw drug markets and tested the authenticity of the herbal drugs using DNA barcoding. These studies reported that 24% of raw drug samples of *Phyllanthus amarus* Schumach. & Thonn. were substituted with other phenotypically similar *Phyllanthus* species (Srirama et al., 2010). Similar substitution were reported for other species, such as *Sida cordifolia* L. (76%) (Vassou et al., 2015), *Cinnamomum verum* J.Presl (70%) (Swetha et al., 2014), *Myristica fragrans* Houtt. (60%) (Swetha et al., 2017), *Senna auriculata* (L.) Roxb. (50%) (Seethapathy et al., 2015), *Senna tora* (L.) Roxb. (37%) (Seethapathy et al., 2015) and *Senna alexandrina* Mill. (8%) (Seethapathy et al., 2015). Furthermore, Vassou et al. (2016) reported that 21% of raw drugs in Indian herbal markets were unauthentic. Shanmughanandhan et al. (2016) found that 60% of 93 herbal products sold in the form of capsules and plant powders in local stores in India were adulterated. Studies that combined spectroscopic methods, such as NMR, with DNA barcoding or microscopy to authenticate herbal products, reported 80% adulteration in *Saraca asoca* (Urumarudappa et al., 2016), 80% in *Berberis aristata* (Srivastava and Rawat, 2013) and 22% in *Piper nigrum* (Parvathy et al., 2014). All these studies utilizing DNA barcoding and metabarcoding have highlighted the concerns over the quality and good labeling practices of herbal products (Coghlan et al., 2012; Ivanova et al., 2016; Raclariu et al., 2017a,b; Veldman et al., 2017).

The aim of this study was threefold. First, we aimed to test the composition and fidelity of Ayurvedic products marketed in Europe using DNA metabarcoding. Secondly, we aimed to analyze the presence of any red listed species listed on the product label and used as ingredients using DNA metabarcoding. Our final aim was to evaluate the ability of DNA metabarcoding to identify the presence of authentic species, any substitution and adulteration and/or presence of other off labeled plant species.

## Materials and Methods

### Sample Collection

Seventy-nine Ayurvedic herbal products sold as tablets (*n* = 30), capsules (*n* = 30), powders (*n* = 16), and extracts (*n* = 3) were purchased via e-commerce (*n* = 53) and pharmacies (*n* = 26), from Norway (*n* = 21), Romania (*n* = 26), and Sweden (*n* = 32). Based on the label information, 26 were single plant ingredient products, 39 contained between two to ten plant ingredients, and 14 products contained between eleven to 27 plant ingredients (Supplementary Table S1). The products contained a total of 159 plant species belonging to 132 genera and 60 families (Supplementary Table S2). It was also confirmed that nrITS sequences of all the 159 plant species labeled in the analyzed herbal products were available within the NCBI/GenBank database (Supplementary Table S2). The accepted binomial names and authors of the plants species used as ingredients were validated using The Plant List (2013). The Ayurvedic herbal products were imported into Norway for scientific analyses under Norwegian Medicines Agency license no. 16/04551–2. An overview of the products, including label information, but not the producer/importer name, lot number, expiration date or any other information that could lead to the identification of that specific product, can be found in Supplementary Table S1.

### DNA Extraction, Amplicon Generation, and High Throughput Sequencing

The 79 Ayurvedic herbal products were processed depending on their pharmaceutical formulation, in addition to an extraction blank per DNA extraction round. A small amount of each herbal product, about 200 mg, was homogenized using 3–5 zirconium grinding beads in a Mini-Beadbeater-1 (Biospec Products Inc., Bartlesville, Oklahoma, United States). The total DNA from each product was extracted from homogenized contents using CTAB extraction (Doyle and Doyle, 1987). The final elution volume was 100 μl. Extracted DNA was quantified using a Qubit 2.0 Fluorometer and Qubit dsDNA HS Assay Kit (Invitrogen, Carlsbad, California, United States). All amplicon libraries, defined as PCR amplified products from a study sample, were prepared in three replicates. For each replicate two nuclear ribosomal target sequences were amplified, the internal transcribed spacers nrITS1 and nrITS2, respectively. The fusion primers included the annealing motif from the Sun et al. (1994) plant-specific primer pairs 17SE and 5.8I1, and 5.8I2 and 26SE. The forward primers included the Ion Torrent A adapter, a 10 bp multiplex identifier tag following the IonXpress setup for Ion Torrent (Thermo Fisher Scientific, Carlsbad, California, United States). The reverse primer included the truncated P1 (trP1) tags in addition to the annealing motif. Expected amplicon sizes were 300–350 bp.

Polymerase chain reactions were carried out using DNA extracted from the herbal products in final reaction volumes of 25 μl including 0.5 μl of template DNA solution (ranging from 0.5 to 2 ng/μl), 1X Q5 reaction buffer (New England Biolabs Inc., United Kingdom), 0.6 μM of each primer (Biolegio B.V., Netherlands), 200 nM dNTPs, 5 U Q5 High-Fidelity DNA Polymerase (New England Biolabs Inc., United Kingdom) and 1X Q5 High GC enhancer. The PCR cycling protocol consisted of initial denaturation at 98°C for 30 s, followed by 35 cycles of denaturation at 98°C for 10 s, annealing at 56°C for nrITS1 or 71°C for nrITS2 for 30 s, and elongation at 72°C for 30 s, followed by a final elongation step at 72°C for 2 min. Three PCR negative controls of the extraction blanks were included per amplification to control for external and cross sample contamination. After PCR, the amplicons were purified using Illustra Exostar (GE Healthcare, Chicago, Illinois, United States) in accordance with the manufacturer protocols. The molarity of each amplicon library was measured using a qPCR based assay (CFX96 Touch Real-Time PCR Detection System, Bio-Rad, Hercules, California, United States). The equimolar amounts of each amplicon library were merged and sequenced using an Ion Torrent Personal Genomic Machine (Thermo Fisher Scientific) as described by Raclariu et al. (2017a).

### Bioinformatics Analysis

The sequencing read data were analyzed and demultiplexed into FASTQ files, per sample, using Torrent Suite version 5.0.4 (LT), and each of the replicates was analyzed individually. FASTQ read files were processed using the HTS-barcode-checker pipeline available as a Galaxy pipeline at the Naturalis Biodiversity Center^1^ (Lammers et al., 2014). Using the HTS pipeline, nrITS1 and nrITS2 primer sequences were used to demultiplex the sequencing reads per sample and to filter out reads that did not match any of the primers. PRINSEQ was used to determine filtering and trimming values based on read lengths and Phred read quality. All reads with a mean Phred quality score of less than 26 were filtered out, as well as reads with a length of less than 200 bp. The remaining reads were trimmed to a maximum length of 380 bp. CD-HIT-EST was used to cluster reads into molecular operational taxonomic units (MOTUs) defined by a sequence similarity of >99% and a minimum number of ten reads. The consensus sequences of non-singleton MOTUs were queried using BLAST against a reference nucleotide sequence database, with a maximum e-value of 0.05, a minimum hit length of 100 bp and sequence identity of >97%. The number of reads per MOTU, as well as the BLAST results per MOTU, were compiled using custom scripts from the HTS Barcode Checker pipeline (Lammers et al., 2014). The reference sequence database consisted of a local copy of the NCBI/GenBank nucleotide database that is refreshed monthly. These parameters were applied to each of the replicates. A species was considered and validated as being present within the product only if this was detected in at least 2 out of the 3 replicates.

### Presence and Abundance of Species Across Samples

To assess species diversity within each sample, and to obtain insights into the dominant species within the Ayurvedic herbal products, the read abundances were normalized by dividing the number of reads for a MOTU by the total number of reads per sample. As a result, the read counts are transformed into a proportion of reads found per species within each sample (Supplementary Tables S3, S4). Furthermore, MOTUs detected in at least two out of the three replicates, for each sample, were categorized into expected-detected (MOTUs corresponding to species listed on the product label versus species detected in the analysis), expected-not detected (MOTUs corresponding to species listed on the product label but not detected in the analysis), and not expected-detected (MOTUs corresponding to species non-listed on the product label but detected in the analysis) (Supplementary Table S5). The total occurrences of MOTUs per category of expected and detected were evaluated (Supplementary Table S5), and a matrix of correlation was generated using ClustVis (Metsalu and Vilo, 2015).

## Results

### Fidelity of Ayurvedic Products

The genomic DNA extracts were highly variable in quantity and quality. Total DNA concentration for each of the 79 herbal products is provided in Supplementary Table S6. Table 1 shows the average DNA yield for each of the investigated herbal product types. The result shows that three samples labeled as containing only standardized extracts yielded an average of 0.5 ng/μl DNA, whereas tablets, capsules and powders yielded an average of 5.8, 9.6, and 44.7 ng/μl DNA, respectively. Out of 79 products used in the study, 10 tablets were also labeled to contain extracts in addition to crude plant material (#6, #12, #13, #14, #17, #18, #20, #21, #38, and #74). PCR amplification for nrITS1 and nrITS2 regions were performed for all 79 samples, and amplicons were generated for all replicates for nrITS1 and nrITS2 (for samples and concentrations see Supplementary Table S6). The extraction blanks yielded no molecular operational taxonomic units (MOTUs) with nrITS1 and nrITS2 primers.

**Table 1:** Genomic DNA yield and amplicon concentrations per herbal product type.

Product type	No. of herbal products	Average genomic DNA concentration (ng/μl) (SD)	Average amplicon concentration quantified by qPCR (ng/μl)						No. of products yielding DNA sequences	No. of products analyzed post filtering
			nrITS1			nrITS2				
			Replicate 1 (SD)	Replicate 2 (SD)	Replicate 3 (SD)	Replicate 1 (SD)	Replicate 2 (SD)	Replicate 3 (SD)		
Tablets	30	5.8 (6)	5.0 (7)	4.3 (4)	7.7 (15)	7.1 (10)	4.9 (4)	15.3 (18)	20	19
Capsules	30	9.6 (12)	6.2 (11)	6.0 (11)	5.1 (9)	5.8 (9)	4.2 (5)	8.3 (13)	16	15
Powders	16	44.7 (63)	19.2 (25)	3.4 (6)	2.4 (3)	9.4 (19)	4.0 (11)	8.3 (18)	5	5
Extracts	3	0.5 (0.5)	3.4 (0.4)	2.4 (2)	2.4 (2)	20.3 (13)	8.5 (4)	21 (19)	3	-


The sequencing success rate was 44% for ITS1 and 41% for ITS2 (Supplementary Table S6). Thirty-five products out of 79 (44%) yielded no MOTUs in any of the replicates either for nrITS1 or nrITS2 that fulfilled our quality criteria, and they were excluded from the results and the further discussion (#11, #20–22, #28, #29, #33, #35, #37–39, #41–51, #53, #54, #56, #57, #59, #62, 64, #65, #67, #71, #72, #76, and #78). These products consisted of 13 tablets, 11 capsules, and 11 powders (Supplementary Table S6). The products that yielded MOTUs were represented by 17 tablets, 19 capsules, 5 powders, and 3 extracts (Supplementary Table S7).

**FIGURE 1:**
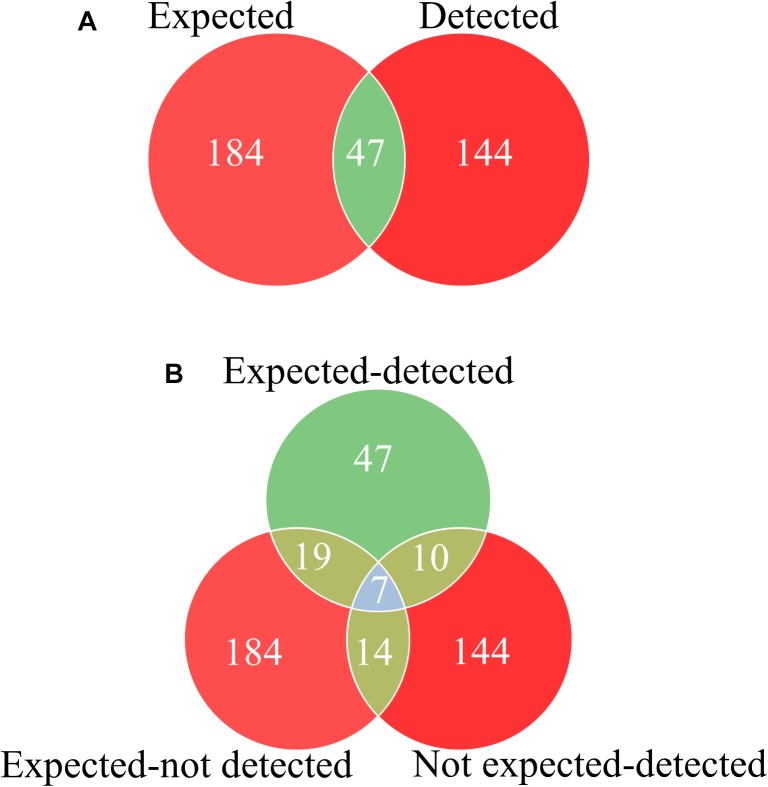
Discrepancies between listed species and detected species using DNA metabarcoding in Ayurvedic herbal products. **(A)** Total number of occurrences of expected species as labeled in the herbal products and detected species using DNA metabarcoding. **(B)** Total number of detected species occurred among expected species as labeled in herbal products (expected-detected), the number of undetected species among the expected species as labeled (expected-not detected), and the number of detected unexpected species (not expected-detected) found in herbal products using DNA metabarcoding. The overlapping numbers are the same species detected in herbal products as expected, detected and unexpected detected.

A total of 188 different plant species belonging to 154 genera and 65 families were identified from the retained MOTUs using BLAST. The separate analyses resulted in 131 plant species (110 genus and 53 families) for nrITS1, and 101 plant species (84 genus and 39 families) for nrITS2. The number of species detected per sample ranged from one to 42. After applying our quality selection criteria, where a species was considered and validated as being present within the product only if it was detected in at least 2 out of the 3 replicates, five additional products (#4, #15, #24, #25, and #26 includes 2 tablets and 3 extracts) that failed to yield the same MOTU in any of the replicates were discarded. The remaining 39 products resulted in a total of 97 plant species belonging to 40 families (62 species for nrITS1, and 60 species for nrITS2). The species detected for all the replicates for both ITS1 and ITS2, were merged for each sample for further analyses (Figure 1 and Supplementary Tables S3, S7).

**FIGURE 2:**
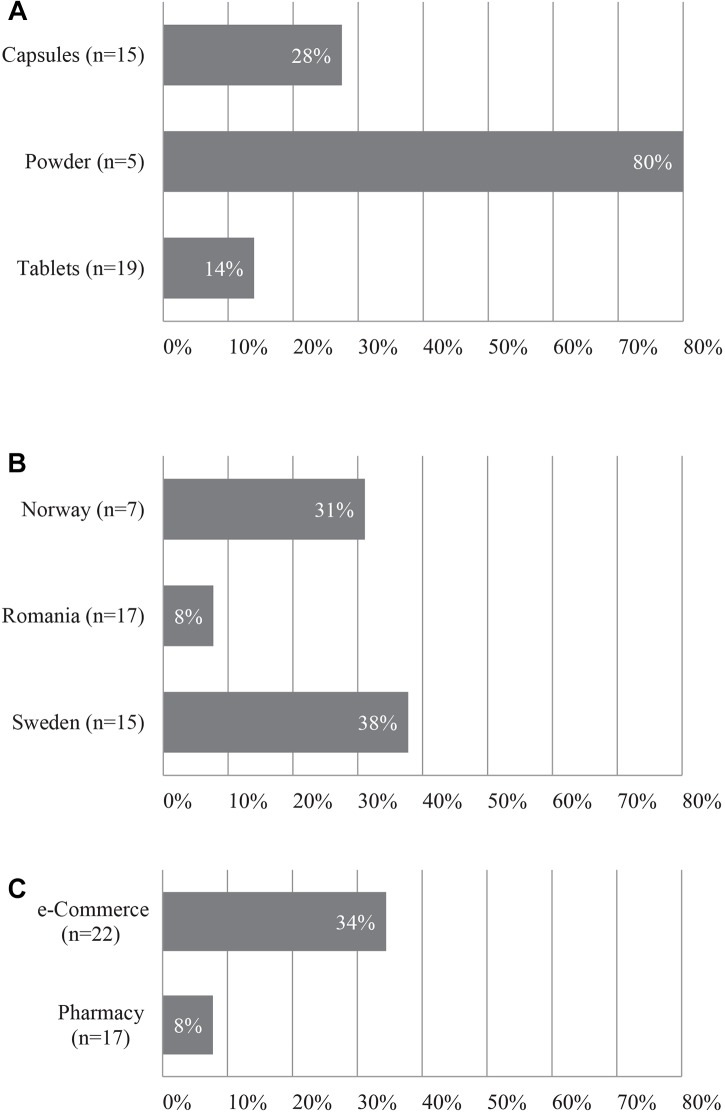
Fidelity of herbal products **(A)** per product form; **(B)** per country; **(C)** per acquisition method. *n* = total number of herbal products.

Figure 2 illustrates the fidelity of herbal products between various product forms, country, and method of acquisition. In ten out of twelve single ingredient products that were labeled as containing only one species, we detected multiple species (exceptions #5 and #52), from which six contained the species labeled on the product together with other species, whereas four products did not contained the species listed on the product label but contained several other non-listed species. Out of 27 successfully analyzed multiple ingredient products, 8 (29.6%) products contained none of the species listed on the label, and the remaining 19 products contained between one to five species listed on the label along with many other species not specified on the product label. The fidelity rate for single ingredient products was 67% (8 out of 12), and the overall ingredient fidelity (detected species from product label/total number of species on label) for multi ingredient products was 21% and for all products 24%. Table 2 shows the top ten products with highest fidelity is also relatively high in the level of substitution, whereas Table 3 shows the top ten products with highest adulteration and its fidelity.

**Table 2:** Top ten products with the highest fidelity and their level of adulteration.

Herbal product code	Product type	No. species on label	Detected by DNA metabarcoding	Fidelity (Expected-detected, absolute)	Fidelity (Expected-detected, relative)	Adulteration (Detected-Not expected, absolute)	Adulteration (Detected-Not expected, relative)
34	Tablets	8	15	5	63%	10	67%
31	Tablets	10	7	5	50%	2	29%
36	Tablets	13	7	4	31%	3	43%
73	Tablets	14	14	3	21%	11	79%
74	Tablets	9	5	3	33%	2	40%
66	Capsules	6	5	3	50%	2	40%
7	Capsules	6	5	2	33%	3	60%
75	Tablets	3	3	2	67%	1	33%
69	Capsules	1	13	1	100%	12	92%
3	Tablets	4	9	1	25%	8	89%


**Table 3:** Top ten products with the highest adulteration and their fidelity.

Herbal product code	Product type	No species on label	Detected by DNA metabarcoding	Fidelity (Expected-detected, absolute)	Fidelity (Expected-detected, relative)	Adulteration (Detected-Not expected, absolute)	Adulteration (Detected-Not expected, relative)
69	Capsules	1	13	1	100%	12	92%
32	Tablets	6	12	0	0%	12	100%
73	Tablets	14	14	3	21%	11	79%
34	Tablets	8	15	5	63%	10	67%
3	Tablets	4	9	1	25%	8	89%
68	Capsules	1	8	0	0%	8	100%
40	Capsules	4	8	1	25%	7	88%
27	Capsules	1	8	1	100%	7	88%
6	Tablets	9	7	1	11%	6	86%
23	Tablets	4	7	1	25%	6	86%


**FIGURE 3:**
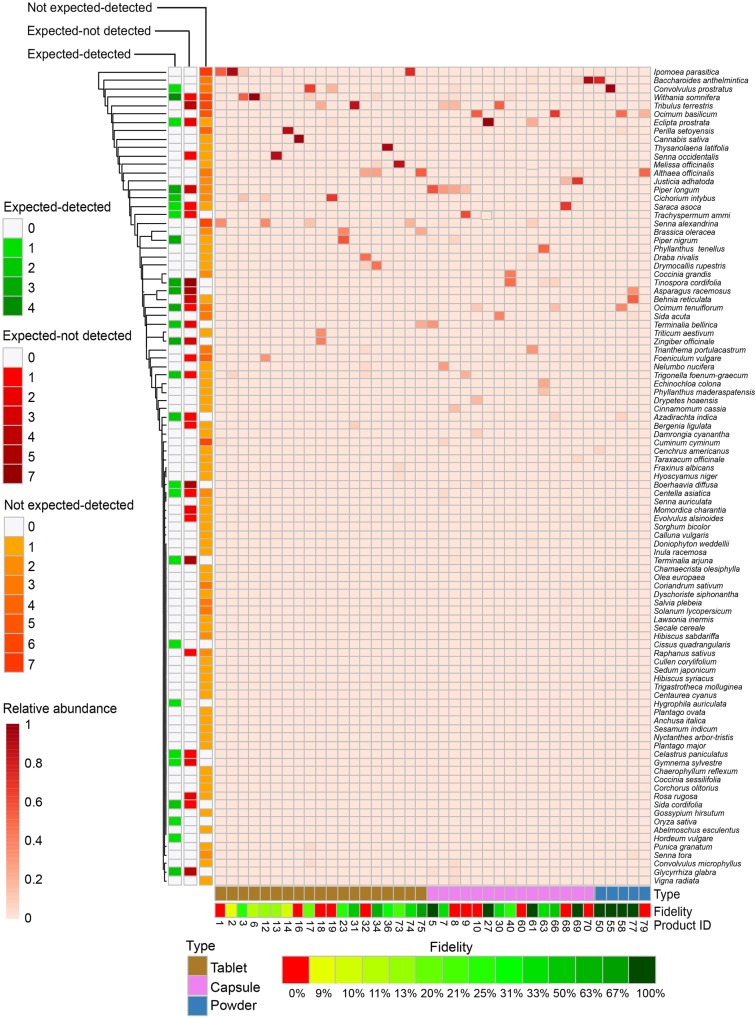
Detection of species in Ayurvedic herbal products. Species (y-axis) are colored by relative abundance of normalized read numbers. Species are categorized in expected-detected and not expected-detected, based on the total number of occurrences, whereas the category expected-not detected is based on the number of times that the species is expected but not detected. Species are clustered by Euclidean distances. Ayurvedic samples (x-axis) are numbered with product code and grouped by product type.

Figure 3 depicts all 97 detected species based on the relative abundance of read numbers in 39 herbal products per type under the categories of expected-detected, expected-not detected, and not expected-detected.

### Plant Ingredients in Herbal Products

**FIGURE 4:**
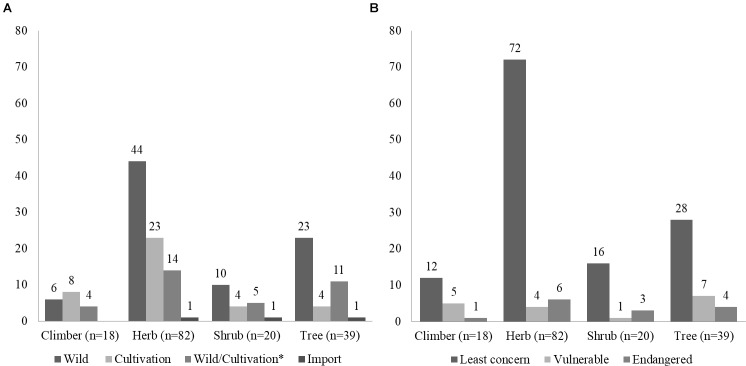
Source and conservation status of species in Ayurvedic products. **(A)** Source of plants labeled as ingredients in the herbal products studied. **(B)** Conservation status of plants labeled as ingredients in the herbal products studied. *N* = total number of species. ^∗^Wild/Cultivation denotes that the plants species are sourced both from wild and cultivation.

A total of 159 plant species belonging to 132 genera and 60 families were specified on the labels of the 79 Ayurvedic herbal products used in this study. Assessing the source and availability of these plants, we found that 83 plants species are solely harvested from wild, and 31 of these are under various threat levels, including critically endangered and protected species, such as *Pterocarpus marsupium* Roxb., *Pterocarpus santalinus* L.f., *Santalum album* L., and *Saraca asoca* (Roxb.) Willd. (Figure 4 and Supplementary Table S2; Ved and Goraya, 2007; Envis Frlht, 2017; Goraya and Ved, 2017). The DNA metabarcoding analysis confirms the presence of four of these threatened species, i.e., *Celastrus paniculatus*, *Glycyrrhiza glabra, Gymnema sylvestre,* and *Saraca asoca*, whereas the remaining threatened species were not detected despite being included as labeled ingredients (Figure 3 and Supplementary Table S7). The following species were found in over 20% of the products: *Withania somnifera* (L.) Dunal (39%), *Tribulus terrestris* L. (27%), *Convolvulus prostratus* Forssk. (23%), *Coriandrum sativum* L. (23%), *Ipomoea parasitica* (Kunth) G. Don (23%), *Ocimum basilicum* L. (23%) and *Senna alexandrina* Mill. (23%) (Figure 3 and Supplementary Table S3). Seventeen are present in more than 10% of samples are listed in the Supplementary Table S3.

## Discussion

The British Pharmacopeia is one the first to publish a specific methods section on DNA barcoding, and in the 2016 version it included a new methods appendix on “Deoxyribonucleic acid (DNA) based identification techniques for herbal drugs” to create a framework for compliance of DNA barcoding with regulatory requirements (British Pharmacopeia Commission, 2016; Sgamma et al., 2017). However, DNA barcoding and metabarcoding are not yet widespread validated methods for use in the regulatory context of quality control. Several studies advocate its usefulness for herbal product authentication and pharmacovigilance either as a standard method or as a complementary method (Ivanova et al., 2016; Raclariu et al., 2017a,b, 2018a; Sgamma et al., 2017). In this study, DNA metabarcoding was used as an analytical approach in Ayurveda herbal product authentication.

A number of studies have shown that the quality of the extraction substrate influences amplification and sequencing success (Ivanova et al., 2016; Raclariu et al., 2017a, 2018b). In addition the presence of DNA in the extraction substrates is influenced by degradation during the harvesting, drying, storage, and industrial processing of plant material (Novak et al., 2007). The success rate in generating raw sequence reads from the herbal products, and the number of products from which MOTUs could be identified per product after applying strict trimming and filtering quality criteria, reduced the number of samples yielding DNA metabarcoding results from 79 to 39 samples. In this study, 44% of products did not yield MOTUs in any of the replicates either for nrITS1 or nrITS2. Also, in the herbal products labeled to contain only extracts, no plant DNA was detected. The undetected MOTUs in these products could be related to the methodological framework of DNA metabarcoding such as DNA extraction protocol, suitability of primer pair sequences, amplification protocols in PCR for the library preparation, sequencing platform, filtering, quality thresholds, and chimera removal, and clustering thresholds (De Boer et al., 2017; Sgamma et al., 2017; Raclariu et al., 2018b). In addition, extraction of crude herbal drugs either in pre-processing or manufacturing can reduce the availability of plant DNA from those species, especially if material is extracted in boiling water or alcohol, and evaporated or dried at high temperatures.

Considerable incongruences were observed between the detected species and those listed on the label of the products. Similarly, Raclariu et al. (2017b) demonstrated the ability of DNA metabarcoding in detecting *Hypericum* species in complex herbal formulations, and revealed the incongruence between constituent species and those listed on the label in all products. Also, De Boer et al. (2017) performed DNA metabarcoding analyses on 55 commercial products based on orchids (*salep*) purchased in Iran, Turkey, Greece, and Germany, and concluded that there are significant differences in labeled and detected species. They also highlighted the applicability of DNA metabarcoding in targeted efforts for conservation of endangered orchid species. In our study, we detected a total of 97 species in 39 products that passed our quality criteria, and most of the identified species are likely ingredients of Ayurvedic herbal products. Detection of certain species is improbable given their distribution or unlikely use, and these include *Achillea millefolium* L., *Anchusa italica* Retz., *Calluna vulgaris* (L.) Hull, *Damrongia cyanantha* Triboun, *Fraxinus albicans* Buckley and *Trigastrotheca molluginea* F.Muell. The identification of these plant species may be explained by *(i)* amplified PCR chimeras; *(ii)* false-positive BLAST identifications due to incomplete or error-prone reference databases; or *(iii)* presence of pollen from wind pollinating species, and this confirms previously raised concerns about the hypersensitivity of DNA metabarcoding (De Boer et al., 2017).

Out of 97 species detected in the DNA metabarcoding analysis, 40 species are sourced from wild, 38 species are cultivated, and 15 species are sourced from both wild and cultivation. Similarly, among the 89 species which were not detected in the analysis, 62 species are mainly sourced from wild, including endangered species such as *Embelia ribes* Burm.f., *Pterocarpus marsupium* Roxb., *Pterocarpus santalinus* L.f., *Pueraria tuberosa* (Willd.) DC., and *Santalum album* L. Understanding, the discrepancies between the species detected using DNA metabarcoding and those listed on the label of the products require careful consideration. In DNA metabarcoding analyses, the level of similarity clustering thresholds (>97, >99, and 100%) have an impact on the number and size of assigned MOTUs (Raclariu et al., 2017a). In this study, we used a 99% clustering threshold similar to previously published studies (Raclariu et al., 2017a; Veldman et al., 2017). Furthermore, to limit the impact of sequencing errors, which are known to affect the Ion Torrent sequencing platform (Salipante et al., 2014) and which could lead to the formation of false MOTUs, we used only the clusters that contained a minimum of 10 reads. In addition, by using three replicates for each sample and marker, we reduced further noise by accepting MOTUs only if present in more than one replicate. Furthermore, the strict filtering and trimming thresholds for base calling, length and quality, and strict clustering criteria for MOTUs formation, increase confidence of the results. As reported by previous studies (Ivanova et al., 2016; Raclariu et al., 2017b), the results related to the authentication of herbal products using DNA metabarcoding need to focus primarily on checking the presence of the labeled ingredients and contaminants. The presence of non-listed species may be explained by various factors, including but not limited to the deliberate adulteration and unintentional substitution that may occur from the early stage of the supply chain of medicinal plants (i.e., cultivation, transport, and storage), to the manufacturing process and the commercialization of the final products. DNA metabarcoding is a highly sensitive method and even traces of DNA, e.g., contamination from grains of pollinating species or plant dust in the manufacturing process, can be detected and identified.

The advantage of DNA metabarcoding is its ability to simultaneously identify total species diversity within complex multi-ingredient and processed mixtures. Importantly, DNA metabarcoding data is used for qualitative evaluation only, to determine presence of taxa, and not for quantitative assessment of relative species abundance based on read numbers, as many variables considerably impact the obtained sequence read results (Staats et al., 2016). In the context of the quality control of herbal products, DNA metabarcoding does not provide any quantitative nor qualitative information of the active metabolites in the raw plant material or the resulting preparation, and this narrows its applicability only to identification and authentication procedures. Thus, if product safety control relies on threshold levels of specific marker compounds, absence of toxins, allergens and admixed pharmaceuticals, then other methods may be more relevant than DNA-based composition analysis. On the other hand, if product fidelity, species substitution or adulteration is suspected then the latter method outperforms in terms of resolution.

The results of this study reveal that there is a need for a better quality control of herbal products. A novel analytical approach should eventually use a combination of innovative high throughput methods that complement the standard ones recommended today.

## Conclusion

Assessment of Ayurvedic herbal medicines using DNA metabarcoding provides insight into species diversity in these products and highlights a marked incongruence between species listed as ingredients on the product labels and those detected from DNA present in the samples. Detection of not-listed and not-expected species first and foremost suggests irregularities in the manufacturing process. The presence of foreign plant material could be due accidental reasons, such as contamination from insufficiently cleaned bags, containers, mills, conveyors, and other equipment, or co-occurrence of weeds in cultivation, pollen from wind pollinated species or seeds from wind-dispersed species. However, foreign plant material could also result from fraud, i.e., substitution, adulteration and/or admixture of other species. Interpretation of incongruences should focus on the detected species in the products, and less on the failure to detect species as there are many steps in manufacturing processes that could lead to degradation or loss of DNA beyond detectable limits, e.g., alcoholic extraction, decoction and drying of material at high temperatures. Our study showed that the investigated herbal products contained species not listed on the product labels, and this reveals a clear need for improved quality control. A novel analytical approach should eventually use a combination of advanced chemical methods and innovative high throughput sequencing to complement the standard ones recommended today. The findings of our study show that DNA metabarocoding is a promising tool for quality evaluation of herbal products and pharmacovigilance, and a good candidate for an effective use as a regulatory tool to authenticate complex herbal products. However, standardization of protocols is necessary before DNA metabarcoding can be implemented as a routine analytical approach and approved by competent authorities for use in a regulatory framework.

## Supporting Information

Ion-Torrent sequencing data is deposited in Zenodo doi: 10.5281/zenodo.2548681.

## Author Contributions

GS, ACRM, HW, and HdB conceived the experiment. GS collected the material and carried out the molecular lab work and analysis together with ACRM. JA carried out high-throughput sequencing together with GS. GS wrote the manuscript together with HdB. All authors contributed to and approved the final version of the manuscript.

## Conflict of Interest Statement

The authors declare that the research was conducted in the absence of any commercial or financial relationships that could be construed as a potential conflict of interest.

## Footnotes

1 http://145.136.240.164:8080/

## References

[B1] Barnes J. (2003). Pharmacovigilance of herbal medicines. *Drug Saf.* 26 829–851. 10.2165/00002018-200326120-00001 12959628

[B2] British Pharmacopeia Commission (2016). *British Pharmacopoeia 2016. Deoxyribonucleic acid (DNA) Based Identification Techniques for Herbal Drugs. Appendix XI V.* London: TSO.

[B3] Calahan J. Howard D. Almalki A. J. Gupta M. P. Calderón A. I. (2016). Chemical adulterants in herbal medicinal products: a review. *Planta Med.* 82 505–515. 10.1055/s-0042-103495 27054916

[B4] Chan K. (2003). Some aspects of toxic contaminants in herbal medicines. *Chemosphere* 52 1361–1371. 10.1016/S0045-6535(03)00471-5 12867165

[B5] Cheng X. Su X. Chen X. Zhao H. Bo C. Xu J. (2014). Biological ingredient analysis of traditional Chinese medicine preparation based on high-throughput sequencing: the story for Liuwei Dihuang Wan. *Sci. Rep.* 4:5147. 10.1038/srep05147 24888649 PMC4042125

[B6] Coghlan M. L. Haile J. Houston J. Murray D. C. White N. E. Moolhuijzen P. (2012). Deep sequencing of plant and animal DNA contained within Traditional Chinese Medicines reveals legality issues and health safety concerns. *PLoS Genet.* 8:e1002657. 10.1371/journal.pgen.1002657 22511890 PMC3325194

[B7] De Boer H. J. Ghorbani A. Manzanilla V. Raclariu A.-C. Kreziou A. Ounjai S. (2017). DNA metabarcoding of orchid-derived products reveals widespread illegal orchid trade. *Proc. R. Soc. Lond. B Biol. Sci.* 284:20171182. 10.1098/rspb.2017.1182 28931735 PMC5627200

[B8] De Boer H. J. Ichim M. C. Newmaster S. G. (2015). DNA barcoding and pharmacovigilance of herbal medicines. *Drug Saf.* 38 611–620. 10.1007/s40264-015-0306-8 26076652

[B9] Doyle J. Doyle J. (1987). A rapid DNA isolation procedure for small quantities of fresh leaf tissue. *Phytochem. Bull* 19 11–15.

[B10] Envis Frlht (2017). *“ENVIS Resource Partner on Medicinal Plants”.* Available at: http://envis.frlht.org/ [accessed July 27 2017].

[B11] Ernst E. (1998). Harmless herbs? A review of the recent literature. *Am. J. Med. Sci.* 104 170–178. 10.1016/S0002-9343(97)00397-5 9528737

[B12] Evans W. C. (2009). *Trease and Evans’ Pharmacognosy E-Book.* Amsterdam: Elsevier Health Sciences.

[B13] Gilbert N. (2011). Regulations: herbal medicine rule book. *Nature* 480 S98–S99. 10.1038/480S98a 22190094

[B14] Goraya G. S. Ved D. K. (2017). *Medicinal Plants in India: An Assessment of their Demand and Supply.* Dehradun: Ministry of AYUSH.

[B15] Hebert P. Cywinska A. Ball S. Ewaard J. (2003). Biological identifications through DNA barcodes. *Proc. R. Soc. Lond. B Biol. Sci.* 270 313–321. 10.1098/rspb.2002.2218 12614582 PMC1691236

[B16] Heubl G. (2010). New aspects of DNA-based authentication of Chinese medicinal plants by molecular biological techniques. *Planta Med.* 76 1963–1974. 10.1055/s-0030-1250519 21058240

[B17] Ivanova N. V. Kuzmina M. L. Braukmann T. W. A. Borisenko A. V. Zakharov E. V. (2016). Authentication of herbal supplements using next-generation sequencing. *PLoS One* 11:e0156426. 10.1371/journal.pone.0156426 27227830 PMC4882080

[B18] Jiang Y. David B. Tu P. Barbin Y. (2010). Recent analytical approaches in quality control of traditional Chinese medicines—a review. *Anal. Chim. Acta* 657 9–18. 10.1016/j.aca.2009.10.024 19951752

[B19] Joshi V. K. Joshi A. Dhiman K. S. (2017). The Ayurvedic Pharmacopoeia of India, development and perspectives. *J. Ethnopharmacol.* 197 32–38. 10.1016/j.jep.2016.07.030 27404231

[B20] Lammers Y. Peelen T. Vos R. A. Gravendeel B. (2014). The HTS barcode checker pipeline, a tool for automated detection of illegally traded species from high-throughput sequencing data. *BMC Bioinformatics* 15:44. 10.1186/1471-2105-15-44 24502833 PMC3922334

[B21] Leonti M. Casu L. (2013). Traditional medicines and globalization: current and future perspectives in ethnopharmacology. *Front. Pharmacol.* 4:92. 10.3389/fphar.2013.00092 23898296 PMC3722488

[B22] Metsalu T. Vilo J. (2015). ClustVis: a web tool for visualizing clustering of multivariate data using principal component analysis and heatmap. *Nucleic Acids Res.* 43 W566–W570. 10.1093/nar/gkv468 25969447 PMC4489295

[B23] Newmaster S. G. Grguric M. Shanmughanandhan D. Ramalingam S. Ragupathy S. (2013). DNA barcoding detects contamination and substitution in North American herbal products. *BMC Med.* 11:222. 10.1186/1741-7015-11-222 24120035 PMC3851815

[B24] Novak J. Grausgruber-Gröger S. Lukas B. (2007). DNA-based authentication of plant extracts. *Food Res. Int.* 40 388–392. 10.1016/j.foodres.2006.10.015

[B25] Parvathy V. A. Swetha V. P. Sheeja T. E. Leela N. K. Chempakam B. Sasikumar B. (2014). DNA barcoding to detect chilli adulteration in traded black pepper powder. *Food Biotechnol.* 28 25–40. 10.1080/08905436.2013.870078

[B26] Parveen I. Gafner S. Techen N. Murch S. J. Khan I. A. (2016). DNA barcoding for the identification of botanicals in herbal medicine and dietary supplements: strengths and limitations. *Planta Med.* 82 1225–1235. 10.1055/s-0042-111208 27392246

[B27] Raclariu A. C. Mocan A. Popa M. O. Vlase L. Ichim M. C. Crisan G. (2017a). Veronica officinalis product authentication using DNA metabarcoding and HPLC-MS reveals widespread adulteration with Veronica chamaedrys. *Front. Pharmacol.* 8:378 10.3389/fphar.2017.00378 PMC5474480 28674497

[B28] Raclariu A. C. Paltinean R. Vlase L. Labarre A. Manzanilla V. Ichim M. C. (2017b). Comparative authentication of *Hypericum perforatum* herbal products using DNA metabarcoding, TLC and HPLC-MS. *Sci. Rep.* 7:1291. 10.1038/s41598-017-01389-w 28465563 PMC5431008

[B29] Raclariu A. C. Heinrich M. Ichim M. C. De Boer H. (2018a). Benefits and limitations of DNA barcoding and metabarcoding in herbal product authentication. *Phytochem. Anal.* 29 123–128. 10.1002/pca.2732 28906059 PMC5836936

[B30] Raclariu A. C. Tebrencu C. E. Ichim M. C. Ciuperca O. T. Brysting A. K. De Boer H. J. (2018b). What’s in the box? Authentication of Echinacea herbal products using DNA metabarcoding and HPTLC. *Phytomedicine* 44 32–38. 10.1016/j.phymed.2018.03.058 29895490

[B31] Rocha T. Amaral J. S. Oliveira M. B. P. P. (2016). Adulteration of dietary supplements by the illegal addition of synthetic drugs: a review. *Compr. Rev. Food Sci. Food Saf.* 15 43–62. 10.1111/1541-4337.12173 33371574

[B32] Salipante S. Kawashima T. Rosenthal C. Hoogestraat D. Cummings L. Sengupta D. (2014). Performance comparison of illumina and ion torrent next-generation sequencing platforms for 16S rRNA-based bacterial community profiling. *Appl. Environ. Microbiol.* 80 7583–7591. 10.1128/AEM.02206-14 25261520 PMC4249215

[B33] Seethapathy G. S. Ganesh D. Santhosh Kumar J. U. Senthilkumar U. Newmaster S. G. Ragupathy S. (2015). Assessing product adulteration in natural health products for laxative yielding plants, Cassia, Senna, and Chamaecrista, in Southern India using DNA barcoding. *Int. J. Legal Med.* 129 693–700. 10.1007/s00414-014-1120-z 25425095

[B34] Sgamma T. Lockie-Williams C. Kreuzer M. Williams S. Scheyhing U. Koch E. (2017). DNA barcoding for industrial quality assurance. *Planta Med.* 83 1117–1129. 10.1055/s-0043-113448 28662530

[B35] Shanmughanandhan D. Ragupathy S. Newmaster S. G. Mohanasundaram S. Sathishkumar R. (2016). Estimating herbal product authentication and adulteration in India using a vouchered, DNA-based biological reference material library. *Drug Saf.* 39 1211–1227. 10.1007/s40264-016-0459-0 27688026

[B36] Srirama R. Senthilkumar U. Sreejayan N. Ravikanth G. Gurumurthy B. R. Shivanna M. B. (2010). Assessing species admixtures in raw drug trade of Phyllanthus, a hepato-protective plant using molecular tools. *J. Ethnopharmacol.* 130 208–215. 10.1016/j.jep.2010.04.042 20435119

[B37] Srivastava S. Rawat A. K. (2013). Quality evaluation of ayurvedic crude drug daruharidra, its allied species, and commercial samples from herbal drug markets of India. *Evid. Based Complement. Alternat. Med.* 2013:472973. 10.1155/2013/472973 23431340 PMC3566491

[B38] Staats M. Arulandhu A. J. Gravendeel B. Holst-Jensen A. Scholtens I. Peelen T. (2016). Advances in DNA metabarcoding for food and wildlife forensic species identification. *Anal. Bioanal. Chem.* 408 4615–4630. 10.1007/s00216-016-9595-8 27178552 PMC4909793

[B39] Sun Y. Skinner D. Z. Liang G. H. Hulbert S. H. (1994). Phylogenetic analysis of sorghum and related taxa using internal transcribed spacers of nuclear ribosomal DNA. *Theor. Appl. Genet.* 89 26–32. 10.1007/BF00226978 24177765

[B40] Swetha V. P. Parvathy V. A. Sheeja T. E. Sasikumar B. (2014). DNA barcoding for discriminating the economically important *Cinnamomum verum* from its adulterants. *Food Biotechnol.* 28 183–194. 10.1080/08905436.2014.931239

[B41] Swetha V. P. Parvathy V. A. Sheeja T. E. Sasikumar B. (2017). Authentication of *Myristica fragrans* Houtt. using DNA barcoding. *Food Control* 73 1010–1015. 10.1016/j.foodcont.2016.10.004

[B42] Taberlet P. Coissac E. Pompanon F. Brochmann C. Willerslev E. (2012). Towards next generation biodiversity assessment using DNA metabarcoding. *Mol. Ecol.* 21 2045–2050. 10.1111/j.1365-294X.2012.05470.x 22486824

[B43] Techen N. Parveen I. Pan Z. Khan I. A. (2014). DNA barcoding of medicinal plant material for identification. *Curr. Opin. Biotechnol.* 25 103–110. 10.1016/j.copbio.2013.09.010 24484887

[B44] The Plant List (2013). *Version 1.1.* Available at: http://www.theplantlist.org/ [accessed July 25 2018].

[B45] Urumarudappa S. K. Gogna N. Newmaster S. G. Venkatarangaiah K. Subramanyam R. Saroja S. G. (2016). DNA barcoding and NMR spectroscopy-based assessment of species adulteration in the raw herbal trade of *Saraca asoca* (Roxb.) Willd, an important medicinal plant. *Int. J. Legal Med.* 130 1457–1470. 10.1007/s00414-016-1436-y 27627901

[B46] Valiathan M. S. (2006). Ayurveda: putting the house in order. *Curr. Sci.* 90 5–6.

[B47] Vassou S. L. Kusuma G. Parani M. (2015). DNA barcoding for species identification from dried and powdered plant parts: a case study with authentication of the raw drug market samples of *Sida cordifolia*. *Gene* 559 86–93. 10.1016/j.gene.2015.01.025 25596347

[B48] Vassou S. L. Nithaniyal S. Raju B. Parani M. (2016). Creation of reference DNA barcode library and authentication of medicinal plant raw drugs used in Ayurvedic medicine. *BMC Complement. Altern. Med.* 16:186. 10.1186/s12906-016-1086-0 27454470 PMC4959393

[B49] Ved D. K. Goraya G. S. (2007). *Demand and Supply of Medicinal Plants in India.* New Delhi: NMPB.

[B50] Veldman S. Gravendeel B. Otieno J. N. Lammers Y. Duijm E. Nieman A. (2017). High-throughput sequencing of African chikanda cake highlights conservation challenges in orchids. *Biodivers. Conserv.* 26 2029–2046. 10.1007/s10531-017-1343-7

[B51] Wang L. Liu L. F. Wang J. Y. Shi Z. Q. Chang W. Q. Chen M. L. (2017). A strategy to identify and quantify closely related adulterant herbal materials by mass spectrometry-based partial least squares regression. *Anal. Chim. Acta* 977 28–35. 10.1016/j.aca.2017.04.023 28577595

[B52] World Health Organization [WHO] (2013). *WHO traditional medicine strategy: 2014-2023.* Available at: http://www.who.int/medicines/publications/traditional/trm_strategy14_23/en/ [accessed July 27 2017].

[B53] Zhang A. Sun H. Yan G. Wang X. (2017). Recent developments and emerging trends of mass spectrometry for herbal ingredients analysis. *Trends Analyt. Chem.* 94 70–76. 10.1016/j.trac.2017.07.007

[B54] Zhang J. Wider B. Shang H. Li X. Ernst E. (2012). Quality of herbal medicines: challenges and solutions. *Complement. Ther. Med.* 20 100–106. 10.1016/j.ctim.2011.09.004 22305255

