# Supplementary material for: DNA Metabarcoding Authentication of Ayurvedic Herbal Products on the European Market Raises Concerns of Quality and Fidelity
Source: Front Plant Sci. 2019 Feb 5;10:68. doi: 10.3389/fpls.2019.00068 (PMC6370972; doi:10.3389/fpls.2019.00068)
Supplement: Supplementary file 1 [file Data_Sheet_1.PDF]

Supplementary Table S1. Ayurvedic herbal products used in the study.

Herbal product ID	Species on label	Product type	Scientific names of the plant ingredients as indicated on the product label	Plant family	Vernacular names on product label
1	7	Tablets	*Acacia arabica* (Lam.) Willd. (= *Acacia nilotica* (L.) Delile)	Leguminosae	
			Achyranthes aspera L.	Amaranthaceae	
			Bergenia ligulata Engl.	Saxifragaceae	
			Boerhaavia diffusa L.	Nyctaginaceae	
			Crateva nurvala Buch.-Ham	Capparaceae	
			Phyllanthus urinaria L.	Phyllanthaceae	
			Piper cubeba Bojer	Piperaceae	
2	11	Tablets	*Acacia arabica* (Lam.) Willd. (= *Acacia nilotica* (L.) Delile)	Leguminosae	
			Azadirachta indica A.Juss.	Meliaceae	
			Curcuma longa L.	Zingiberaceae	
			Enicostemma littorale Blume	Gentianacae	
			Gymnema sylvestre (Retz.) R.Br. ex Sm.	Apocynaceae	
			Momordica charantia L.	Cucurbitaceae	
			Piper longum L.	Piperaceae	
			Pterocarpus marsupium Roxb.	Leguminosae	
			Syzygium cumini (L.) Skeels	Myrtaceae	
			Tinospora cordifolia (Willd.) Miers	Menispermaceae	
			Trigonella foenum-graecum L.	Leguminosae	
3	4	Tablets	*Acacia arabica* (Lam.) Willd. (= *Acacia nilotica* (L.) Delile)	Leguminosae	
			Asparagus racemosus Willd.	Asparagaceae	
			Chlorophytum arundinaceum Baker	Asparagaceae	
			Withania somnifera (L.) Dunal	Solanaceae	
4	4	Tablets	*Eclipta prostrata* (L.) L.	Compositae	
			Guma acacia [$]		
			Mandur bhasma [$]		
			Phyllanthus urinaria L.	Phyllanthaceae	
			Picrorhiza kurroa Royle ex Benth.	Plantaginaceae	
			Piper longum L.	Piperaceae	
5	1	Capsules	*Withania somnifera* (L.) Dunal	Solanaceae	
6	9	Tablets	*Eclipta alba* (L.) Hassk. (= *Eclipta prostrata* (L.) L.)	Compositae	
			Phyllanthus niruri L.	Phyllanthaceae	

			Rheum emodi L.*	Polygonaceae	
			Capparis spinosa L.*	Capparaceae	
			Tephrosia purpurea (L.) Pers.*	Leguminosae	
			Picrorhiza kurroa Royle ex Benth.*	Plantaginaceae	
			Aloe barbadensis Mill. (= *Aloe vera* (L.) Burm.f.)*	Xanthorrhoeaceae	
			Cichorium intybus L. *	Compositae	
			Boerhaavia diffusa L.*	Nyctaginaceae	
7	6	Capsules	*Allium sativum* L.	Amaryllidaceae	
			Pueraria tuberosa (Willd.) DC.	Leguminosae	
			Trigonella foenum-graecum L.	Leguminosae	
			Withania somnifera (L.) Dunal	Solanaceae	
			Asparagus racemosus Willd.	Asparagaceae	
			Leptadenia reticulata (Retz.) Wight & Arn.	Apocynaceae	
8	2	Capsules	*Albizia lebbeck* (L.) Benth.	Leguminosae	
			Curcuma longa L.	Zingiberaceae	
9	2	Capsules	*Valeriana officinalis* L.	Caprifoliaceae	
			Humulus lupulus L.	Cannabaceae	
10	7	Capsules	*Picrorhiza kurroa* Royle ex Benth.	Plantaginaceae	
			Eclipta alba (L.) Hassk. (= *Eclipta prostrata* (L.) L.)	Compositae	
			Phyllanthus niruri L.	Phyllanthaceae	
			Bacopa monnieri (L.) Wettst.	Plantaginaceae	
			Boerhaavia diffusa L.	Nyctaginaceae	
			Camellia sinensis (L.) Kuntze	Theaceae	
			Piper longum L.	Piperaceae	
11	5	Capsules	*Azadirachta indica* A.Juss.	Meliaceae	
			Rubia cordifolia L.	Rubiaceae	
			Curcuma longa L.	Zingiberaceae	
			Terminalia chebula Retz.	Combretaceae	
			Acacia catechu (L.f.) Willd.	Leguminosae	
12	22	Tablets	*Bacopa monnieri* (L.) Wettst.*	Plantaginaceae	
			Centella asiatica (L.) Urb. *	Apiaceae	
			Withania somnifera (L.) Dunal*	Solanaceae	
			Evolvulus alsinoides (L.) L.*	Convolvulaceae	
			Nardostachys jatamansi (D.Don) DC.*	Caprifoliaceae	
			Valeriana wallichii DC. (= *Valeriana jatamansi* Jones)*	Caprifoliaceae	
			Embelia ribes Burm.f.*	Primulaceae	

			Prunus amygdalus Stokes*	Rosaceae	
			Acorus calamus L.*	Acoraceae	
			Tinospora cordifolia (Willd.) Miers *	Menispermaceae	
			Terminalia chebula Retz.*	Combretaceae	
			Emblica officinalis Gaertn. (= *Phyllanthus emblica* L.)*	Phyllanthaceae	
			Oroxylum indicum (L.) Kurz i*	Bignoniaceae	
			Celastrus paniculatus Willd.*	Celastraceae	
			Bacopa monnieri (L.) Wettst.	Plantaginaceae	
			Withania somnifera (L.) Dunal	Solanaceae	
			Mucuna pruriens (L.) DC.	Leguminosae	
			Elettaria cardamomum (L.) Maton	Zingiberaceae	
			Terminalia arjuna (Roxb. ex DC.) Wight & Arn.	Combretaceae	
			Anethum sowa Roxb. ex Fleming	Apiaceae	
			Ipomoea digitata L. (= *Ipomoea cheirophylla* O'Donell)	Convolvulaceae	
			Zingiber officinale Roscoe	Zingiberaceae	
13	16	Tablets	*Capparis spinosa* L.	Capparaceae	Himsra
			Cichorium intybus L.	Compositae	Kasani
			Solanum nigrum L.*	Solanaceae	Kakamachi
			Terminalia arjuna (Roxb. ex DC.) Wight & Arn.*	Combretaceae	Arjuna
			Cassia occidentalis L. (= *Senna occidentalis* (L.) Link)*	Leguminosae	Kasamarda
			Achillea millefolium L.*	Compositae	Biranjasipha
			Tamarix gallica L*	Tamaricaceae	Jhavuka
			Phyllanthus amarus Schumach. & Thonn.	Phyllanthaceae	
			Boerhaavia diffusa L.	Nyctaginaceae	
			Tinospora cordifolia (Willd.) Miers	Menispermaceae	
			Raphanus sativus L. (= *R. raphanistrum* subsp. *sativus* (L.) Domin)	Brassicaceae	
			Emblica officinalis Gaertn. (= *Phyllanthus emblica* L.)	Phyllanthaceae	
			Plumbago zeylanica L.	Plumbaginaceae	
			Embelia ribes Burm.f.	Primulaceae	
			Terminalia chebula Retz.	Combretaceae	
			Fumaria officinalis L.	Papaveraceae	
14	27	Tablets	*Gymnema sylvestre* (Retz.) R.Br. ex Sm.*	Apocynaceae	
			Pterocarpus marsupium Roxb.*	Leguminosae	
			Glycyrrhiza glabra L.*	Leguminosae	
			Casearia esculenta Roxb.*	Salicaceae	
			Eugenia jambolana Lam. (= *Syzygium cumini* (L.) Skeels)*	Myrtaceae	

			Asparagus racemosus Willd.*	Asparagaceae	
			Boerhaavia diffusa L.*	Nyctaginaceae	
			Sphaeranthus indicus L.*	Compositae	
			Tinospora cordifolia (Willd.) Miers *	Menispermaceae	
			Swertia chirata Buch.-Ham. ex Wall.*	Gentianaceae	
			Tribulus terrestris L.*	Zygophyllaceae	
			Phyllanthus amarus Schumach. & Thonn.*	Phyllanthaceae	
			Gmelina arborea Roxb.*	Lamiaceae	
			Gossypium herbaceum L.*	Malvaceae	
			Berberis aristata DC.*	Berberidaceae	
			Aloe barbadensis Mill. (= *Aloe vera* (L.) Burm.f.)*	Xanthorrhoeaceae	
			Terminalia bellirica (Gaertn.) Roxb.	Combretaceae	
			Emblica officinalis Gaertn. (= *Phyllanthus emblica* L.)	Phyllanthaceae	
			Terminalia chebula Retz.	Combretaceae	
			Momordica charantia L.	Cucurbitaceae	
			Ocimum tenuiflorum L.	Lamiaceae	
			Abutilon indicum (L.) Sweet	Malvaceae	
			Rumex maritimus L.	Polygonaceae	
			Curcuma longa L.	Zingiberaceae	
			Piper nigrum L.	Piperaceae	
			Piper longum L.	Piperaceae	
			Zingiber officinale Roscoe	Zingiberaceae	
15	27	Capsules	*Saraca asoca* (Roxb.) Willd.	Leguminosae	Ashoka
			Aegle marmelos (L.) Corrêa	Rutaceae	Dashamoola #
			Premna integrifolia Willd. (= *Premna serratifolia* L.)	Lamiaceae	Dashamoola #
			Oroxylum indicum (L.) Kurz	Bignoniaceae	Dashamoola #
			Stereospermum suaveolens (Roxb.) DC. (= *S. chelonoides* (L.f.) DC.)	Bignoniaceae	Dashamoola #
			Gmelina arborea Roxb.	Lamiaceae	Dashamoola #
			Solanum indicum L.	Solanaceae	Dashamoola #
			Solanum xanthocarpum Schrad. & H. Wendl. (= *S. virginianum* L.)	Solanaceae	Dashamoola #
			Desmodium gangeticum (L.) DC.	Leguminosae	Dashamoola #
			Uraria picta (Jacq.) DC.	Leguminosae	Dashamoola #
			Tribulus terrestris L.	Zygophyllaceae	Dashamoola #
			Symplocos racemosa Roxb.	Symplocaceae	Lodhra
			Tinospora cordifolia (Willd.) Miers	Menispermaceae	Guduchi
			Solanum nigrum L.	Solanaceae	Kakamachi

			Boerhaavia diffusa L.	Nyctaginaceae	Punarnava
			Asparagus racemosus Willd.	Asparagaceae	Shatavari
			Aloe barbadensis Mill. (= *Aloe vera* (L.) Burm.f.)	Xanthorrhoeaceae	Kumari
			Santalum album L.	Santalaceae	Chandana
			Cyperus rotundus L.	Cyperaceae	Musta
			Justicia adhatoda L.	Acanthaceae	Vasaka
			Terminalia bellirica (Gaertn.) Roxb.	Combretaceae	Triphala #
			Emblica officinalis Gaertn. (= *Phyllanthus emblica* L.)	Phyllanthaceae	Triphala #
			Terminalia chebula Retz.	Combretaceae	Triphala #
			Piper nigrum L.	Piperaceae	Trikatu #
			Piper longum L.	Piperaceae	Trikatu #
			Zingiber officinale Roscoe	Zingiberaceae	Trikatu #
			Bombax ceiba L.	Malvaceae	Shalmali #
16	8	Tablets	*Zingiber officinale* Roscoe	Zingiberaceae	Ginger
			Didymocarpus pedicellata (Ait.) Ait. F.	Asclepiadaceae	Shilapuspha
			Saxifraga ligulata Murray (= *Saxifraga stolonifera* Curtis)	Saxifragaceae	Pasanabheda
			Rubia cordifolia L.	Rubiaceae	Indian madder
			Cyperus scariosus R.Br.	Cyperaceae	Umbrella's edge
			Achyranthes aspera L.	Amaranthaceae	Prickly chaff flower
			Onosma bracteatum Wall	Boraginaceae	Sedge
			Vernonia cinerea (L.) Less. (= *Cyanthillium cinereum* (L.) H.Rob.)	Compositae	Purple fleabane (Sahadevi) #
17	6	Tablets	*Saraca indica* L.	Leguminosae	Ashoka
			Asparagus racemosus Willd.*	Asparagaceae	Shatavari
			Terminalia chebula Retz.	Combretaceae	Haritaki
			Sida cordifolia L.	Malvaceae	Bala
			Glycyrrhiza glabra L.	Leguminosae	Yashtimadhu
			Centella asiatica (L.) Urb.	Apiaceae	Mandukaparni
18	6	Tablets	*Boswellia serrata* Roxb. ex Colebr.*	Burseraceae	Shallaki
			Commiphora wightii (Arn.) Bhandari*	Burseraceae	Guggul
			Alpinia galanga (L.) Willd.*	Zingiberaceae	Java galangal
			Glycyrrhiza glabra L.*	Leguminosae	Licorice
			Tribulus terrestris L.	Zygophyllaceae	Small caltrops
			Tinospora cordifolia (Willd.) Miers	Menispermaceae	Tinospora Gulancha
19	4	Tablets	*Tribulus terrestris* L.	Zygophyllaceae	Small caltrop
			Caesalpinia bonducella (L.) Fleming (= *C. bonduc* (L.) Roxb.)	Leguminosae	Bonduc nut

			Asparagus racemosus Willd.	Asparagaceae	Asparagus
			Crateva nurvala Buch.-Ham	Capparaceae	Three leaved caper
			Akik pishti [$]		Processed agate
20	6	Tablets	*Commiphora wightii* (Arn.) Bhandari	Burseraceae	Indian bedellium
			Tinospora cordifolia (Willd.) Miers	Menispermaceae	Gulancha tinospora
			Rubia cordifolia L.*	Rubiaceae	Indian madder
			Emblica officinalis Gaertn. (= *Phyllanthus emblica* L.)*	Phyllanthaceae	Indian gooseberry
			Moringa pterygosperma Gaertn.*	Moringaceae	Horse-radish tree
			Glycyrrhiza glabra L.*	Leguminosae	Licorice
21	5	Tablets	*Commiphora wightii* (Arn.) Bhandari*	Burseraceae	Guggulu
			Garcinia cambogia (Gaertn.) Desr. (= *G. gummi-gutta* (L.) Roxb.)*	Clusiaceae	
			Gymnema sylvestre (Retz.) R.Br. ex Sm.*	Apocynaceae	Meshashringi
			Terminalia chebula Retz.*	Combretaceae	
			Trigonella foenum-graecum L.*	Leguminosae	Medhika
22	7	Tablets	*Tinospora cordifolia* (Willd.) Miers	Menispermaceae	
			Zingiber officinale Roscoe	Zingiberaceae	
			Mentha arvensis L.	Lamiaceae	
			Moringa pterygosperma Gaertn.	Moringaceae	
			Carica papaya L.	Caricaceae	
			Citrus limon (L.) Osbeck	Rutaceae	
			Kaempferia galanga L.	Zingiberaceae	
23	4	Tablets	*Asteracantha longifolia* Nees (= *Hygrophila auriculata* (Schumach.) Heine)	Acanthaceae	
			Prunus amygdalus Batsch (= *Prunus dulcis* (Mill.) D.A.Webb)	Rosaceae	
			Crocus sativus L.	Iridaceae	
			Tribulus terrestris L.	Zygophyllaceae	
24	19	Extracts	*Commiphora wightii* (Arn.) Bhandari	Burseraceae	
			Vitis vinifera L.	Vitaceae	
			Ocimum tenuiflorum L.	Lamiaceae	
			Hyssopus officinalis L.	Lamiaceae	
			Tinospora cordifolia (Willd.) Miers	Menispermaceae	
			Justicia adhatoda L.	Acanthaceae	
			Myristica fragrans Houtt.	Myristicaceae	
			Glycyrrhiza glabra L.	Leguminosae	
			Onosma bracteatum Wall	Boraginaceae	
			Viola odorata L.	Violaceae	

			Terminalia bellirica (Gaertn.) Roxb.	Combretaceae	Triphala #
			Emblica officinalis Gaertn. (= *Phyllanthus emblica* L.)	Phyllanthaceae	Triphala #
			Terminalia chebula Retz.	Combretaceae	Triphala #
			Piper nigrum L.	Piperaceae	Trikatu #
			Piper longum L.	Piperaceae	Trikatu #
			Zingiber officinale Roscoe	Zingiberaceae	Trikatu #
			Embelia ribes Burm.f.	Primulaceae	
			Solanum xanthocarpum Schrad. & H. Wendl. (= *S. virginianum* L.)	Solanaceae	
			Cinnamomum cassia (L.) J.Presl	Lauraceae	
25	19	Extracts	*Commiphora wightii* (Arn.) Bhandari	Burseraceae	
			Vitis vinifera L.	Vitaceae	
			Ocimum tenuiflorum L.	Lamiaceae	
			Hyssopus officinalis L.	Lamiaceae	
			Tinospora cordifolia (Willd.) Miers	Menispermaceae	
			Justicia adhatoda L.	Acanthaceae	
			Myristica fragrans Houtt.	Myristicaceae	
			Glycyrrhiza glabra L.	Leguminosae	
			Onosma bracteatum Wall	Boraginaceae	
			Viola odorata L.	Violaceae	
			Terminalia bellirica (Gaertn.) Roxb.	Combretaceae	Triphala #
			Emblica officinalis Gaertn. (= *Phyllanthus emblica* L.)	Phyllanthaceae	Triphala #
			Terminalia chebula Retz.	Combretaceae	Triphala #
			Piper nigrum L.	Piperaceae	Trikatu #
			Piper longum L.	Piperaceae	Trikatu #
			Zingiber officinale Roscoe	Zingiberaceae	Trikatu #
			Embelia ribes Burm.f.	Primulaceae	
			Solanum xanthocarpum Schrad. & H. Wendl. (= *S. virginianum* L.)	Solanaceae	
			Cinnamomum cassia (L.) J.Presl	Lauraceae	
26	14	Extracts	*Justicia adhatoda* L.	Acanthaceae	
			Althaea officinalis L.	Malvaceae	
			Glycyrrhiza glabra L.	Leguminosae	
			Ocimum tenuiflorum L.	Lamiaceae	
			Piper longum L.	Piperaceae	
			Ziziphus sativa Gaertn. (= *Ziziphus jujuba* Mill.)	Rhamnaceae	
			Alpinia galanga (L.) Willd.	Zingiberaceae	
			Hyssopus officinalis L.	Lamiaceae	

			Viola odorata L.	Violaceae	
			Zingiber officinale Roscoe	Zingiberaceae	
			Piper nigrum L.	Piperaceae	
			Curcuma longa L.	Zingiberaceae	
			Terminalia bellirica (Gaertn.) Roxb.	Combretaceae	
			Mentha × piperita L.	Lamiaceae	
27	1	Capsules	*Gymnema sylvestre* (Retz.) R.Br. ex Sm.	Apocynaceae	
28	1	Capsules	*Garcinia cambogia* (Gaertn.) Desr. (= *G. gummi-gutta* (L.) Roxb.)	Clusiaceae	
29	1	Capsules	*Andrographis paniculata* (Burm.f.) Nees	Acanthaceae	
30	3	Capsules	*Emblica officinalis* Gaertn. (= *Phyllanthus emblica* L.)	Phyllanthaceae	
			Terminalia chebula Retz.	Combretaceae	
			Terminalia bellirica (Gaertn.) Roxb.	Combretaceae	
31	10	Tablets	*Ocimum tenuiflorum* L.	Lamiaceae	
			Curcuma longa L.	Zingiberaceae	
			Emblica officinalis Gaertn. (= *Phyllanthus emblica* L.)	Phyllanthaceae	
			Terminalia bellirica (Gaertn.) Roxb.	Combretaceae	
			Terminalia chebula Retz.	Combretaceae	
			Glycyrrhiza glabra L.	Leguminosae	
			Zingiber officinale Roscoe	Zingiberaceae	
			Piper longum L.	Piperaceae	
			Piper nigrum L.	Piperaceae	
			Cinnamomum zeylanicum Blume (= *C. verum* J.Presl)	Lauraceae	
32	6	Tablets	*Saxifraga granulata* L.	Saxifragaceae	
			Hemidesmus indicus (L.) R. Br. ex Schult.	Apocynaceae	
			Sphaeranthus indicus L.	Compositae	
			Curcuma longa L.	Zingiberaceae	
			Santalum album L.	Santalaceae	
			Carum copticum (L.) Benth. & Hook.f. ex C.B.Clarke (= *Trachyspermum ammi* (L.) Sprague)	Apiaceae	
			Corallium rubrum $		
33	6	Tablets	*Emblica officinalis* Gaertn. (= *Phyllanthus emblica* L.)	Phyllanthaceae	
			Asparagus racemosus Willd.	Asparagaceae	
			Glycyrrhiza glabra L.	Leguminosae	
			Mucuna pruriens (L.) DC.	Leguminosae	
			Terminalia chebula Retz.	Combretaceae	
			Zingiber officinale Roscoe	Zingiberaceae	

			Mytillus margaretiferous $		
34	8	Tablets	*Glycyrrhiza glabra* L.	Leguminosae	
			Terminalia arjuna (Roxb. ex DC.) Wight & Arn.	Combretaceae	
			Amomum subulatum Roxb.	Zingiberaceae	
			Piper longum L.	Piperaceae	
			Piper nigrum L.	Piperaceae	
			Zingiber officinale Roscoe	Zingiberaceae	
			Cinnamomum zeylanicum Blume (= *C.verum* J.Presl)	Lauraceae	
			Iris × germanica L.	Iridaceae	
35	10	Tablets	*Boswellia serrata* Roxb. ex Colebr.	Burseraceae	
			Commiphora mukul (Hook. ex Stocks) Engl.	Burseraceae	
			Curcuma longa L.	Zingiberaceae	
			Allium sativum L.	Amaryllidaceae	
			Zingiber officinale Roscoe	Zingiberaceae	
			Piper nigrum L.	Piperaceae	
			Piper longum L.	Piperaceae	
			Piper chaba Hunter (= *Piper retrofractum* Vahl)	Piperaceae	
			Apium graveolens L.	Apiaceae	
			Sphaeranthus indicus L.	Compositae	
36	13	Tablets	*Terminalia chebula* Retz.	Combretaceae	
			Andrographis paniculata (Burm.f.) Nees	Acanthaceae	
			Curcuma longa L.	Zingiberaceae	
			Zingiber officinale Roscoe	Zingiberaceae	
			Trigonella foenum-graecum L.	Leguminosae	
			Swertia chirata Buch.-Ham. ex Wall.	Gentianaceae	
			Carum copticum (L.) Benth. & Hook.f. ex C.B.Clarke (= *Trachyspermum ammi* (L.) Sprague)	Apiaceae	
			Piper longum L.	Piperaceae	
			Myristica fragrans Houtt.	Myristicaceae	
			Lotus arabicus L.	Leguminosae	
			Boswellia serrata Roxb. ex Colebr.	Burseraceae	
			Cypraea moneta $		
			Terminalia bellirica (Gaertn.) Roxb.	Combretaceae	
			Emblica officinalis Gaertn. (= *Phyllanthus emblica* L.)	Phyllanthaceae	
37	8	Tablets	*Commiphora mukul* (Hook. ex Stocks) Engl.	Burseraceae	
			Bauhinia variegata L.	Leguminosae	

			Hemidesmus indicus (L.) R. Br. ex Schult.	Apocynaceae	
			Curcuma longa L.	Zingiberaceae	
			Glycyrrhiza glabra L.	Leguminosae	
			Saxifraga granulata L.	Saxifragaceae	
			Hordeum vulgare L.	Poaceae	
			Santalum album L.	Santalaceae	
38	12	Tablets	*Commiphora mukul* (Hook. ex Stocks) Engl.	Burseraceae	
			Hibiscus rosa-sinensis L.	Malvaceae	
			Corallium rubrum $		
			Asparagus racemosus Willd.	Asparagaceae	
			Hemidesmus indicus (L.) R. Br. ex Schult.	Apocynaceae	
			Glycyrrhiza glabra L.	Leguminosae	
			Curcuma longa L.	Zingiberaceae	
			Emblica officinalis Gaertn. (= *Phyllanthus emblica* L.)	Phyllanthaceae	
			Terminalia bellirica (Gaertn.) Roxb.	Combretaceae	
			Terminalia chebula Retz.	Combretaceae	
			Zingiber officinale Roscoe	Zingiberaceae	
			Piper longum L.	Piperaceae	
			Piper nigrum L.	Piperaceae	
			Aloe mucilagines $		
39	6	Tablets	*Bacopa monnieri* (L.) Wettst.	Plantaginaceae	
			Alpinia galanga (L.) Willd.	Zingiberaceae	
			Glycyrrhiza glabra L.	Leguminosae	
			Emblica officinalis Gaertn. (= *Phyllanthus emblica* L.)	Phyllanthaceae	
			Santalum album L.	Santalaceae	
			Myristica fragrans Houtt.	Myristicaceae	
			Corallium rubrum $		
40	4	Capsules	*Terminalia arjuna* (Roxb. ex DC.) Wight & Arn.	Combretaceae	Arjun
			Cissus quadrangularis L.	Vitaceae	Harjor
			Phyllanthus emblica L.	Phyllanthaceae	Amalaki
			Ocimum gratissimum L.	Lamiaceae	Vana Tulsi
41	4	Capsules	*Inula racemosa* Hook.f.	Compositae	Pushkarmool
			Ocimum tenuiflorum L.	Lamiaceae	Krishna Tulsi
			Terminalia bellirica (Gaertn.) Roxb.	Combretaceae	Vibhitaki fruit
			Piper longum L.	Piperaceae	Pipali frukt

42	1	Capsules	*Ocimum tenuiflorum* L.	Lamiaceae	Krishna Tulsi, Rama Tulsi, Vana Tulsi
43	3	Capsules	*Terminalia arjuna* (Roxb. ex DC.) Wight & Arn.	Combretaceae	Arjuna
			Sapindus trifoliatus L.	Sapindaceae	Reetha
			Moringa oleifera Lam.	Moringaceae	Sahijan blad
44	3	Capsules	*Coccinia indica* Wight & Arn. (= *Coccinia grandis* (L.) Voigt)	Cucurbitaceae	Bimbi blad
			Bougainvillea spectabilis Willd.	Nyctaginaceae	Bouginvellea blad
			Vinca rosea L. (= *Catharanthus roseus* (L.) G.Don)	Apocynaceae	Sadabahar blad
45	3	Capsules	*Aegle marmelos* (L.) Corrêa	Rutaceae	Bel blad
			Lepidium sativum L.	Brassicaceae	Chandrashoor frø
			Plantago ovata Forssk.	Plantaginaceae	Psyllium Husk
46	3	Capsules	*Picrorhiza kurroa* Royle ex Benth.	Plantaginaceae	Katuki
			Ocimum tenuiflorum L.	Lamiaceae	Krishna Tulsi
			Ocimum gratissimum L.	Lamiaceae	Vana Tulsi
47	3	Capsules	*Phyllanthus niruri* L.	Phyllanthaceae	Bhumyamalaki hel urt
			Picrorhiza kurroa Royle ex Benth.	Plantaginaceae	Katuki root
			Boerhaavia diffusa L.	Nyctaginaceae	Pumarnava root
48	1	Powder	*Althaea officinalis* L.	Malvaceae	Marshmallow root
49	1	Powder	*Hemidesmus indicus* (L.) R. Br. ex Schult.	Apocynaceae	
50	1	Powder	*Centella asiatica* (L.) Urb.	Apiaceae	
51	10	Powders	*Aegle marmelos* (L.) Corrêa	Rutaceae	Bilva root
			Premna integrifolia Willd. (= *P. serratifolia* L.)	Lamiaceae	Agnimantha root
			Oroxylum indicum (L.) Kurz	Bignoniaceae	Shyonaka root
			Stereospermum suaveolens (Roxb.) DC. (= *S. chelonoides* (L.f.) DC.)	Bignoniaceae	Patala root
			Gmelina arborea Roxb.	Lamiaceae	Kashmari root
			Solanum indicum L.	Solanaceae	Bruhati root
			Solanum xanthocarpum Schrad. & H.Wendl. (= *S. virginianum* L.)	Solanaceae	Kantakari root
			Desmodium gangeticum (L.) DC.	Leguminosae	Shalaparni root
			Uraria picta (Jacq.) DC.	Leguminosae	Prushniparni root
			Tribulus terrestris L.	Zygophyllaceae	Gokshura root
52	1	Powder	*Eclipta alba* (L.) Hassk. (= *Eclipta prostrata* (L.) L.)	Compositae	Bringaraj
53	1	Powder	*Bacopa monnieri* (L.) Wettst.	Plantaginaceae	Brahmi
54	1	Powder	*Boerhaavia diffusa* L.	Nyctaginaceae	Punarnava
55	1	Powder	*Sida cordifolia* L.	Malvaceae	

56	1	Powder	*Rubia cordifolia* L.	Rubiaceae	Manjistha
57	1	Powder	*Gymnema sylvestre* (Retz.) R.Br. ex Sm.	Apocynaceae	Madhunashini
58	1	Powder	*Tinospora cordifolia* (Willd.) Miers	Menispermaceae	Guduchi
59	1	Capsules	*Garcinia indica* (Thouars) Choisy	Clusiaceae	
60	1	Capsules	*Terminalia arjuna* (Roxb. ex DC.) Wight & Arn.	Combretaceae	
61	1	Capsules	*Boerhaavia diffusa* L.	Nyctaginaceae	Punarnava
62	1	Powder	*Bacopa monnieri* (L.) Wettst.	Plantaginaceae	
63	3	Capsules	*Phyllanthus niruri* L.	Phyllanthaceae	Bhumiamalaki
			Tinospora cordifolia (Willd.) Miers	Menispermaceae	Amalaki
			Phyllanthus emblica L.	Phyllanthaceae	Guduchi
64	4	Capsules	*Bacopa monnieri* (L.) Wettst.	Plantaginaceae	Brahmi
			Centella asiatica (L.) Urb.	Apiaceae	Gotu Kola
			Convolvulus pluricaulis Choisy (= *C. prostratus* Forssk.)	Convolvulaceae	Shankpushpi
			Withania somnifera (L.) Dunal	Solanaceae	Ashwagandha
65	4	Capsules	*Cyperus rotundus* L.	Cyperaceae	Motha rhizome
			Withania somnifera (L.) Dunal	Solanaceae	Ashwagandha
			Tinospora cordifolia (Willd.) Miers	Menispermaceae	Guduchi
			Ocimum tenuiflorum L.	Lamiaceae	Rama Tulsi
66	6	Capsules	*Rubia cordifolia* L.	Rubiaceae	Manjit rot
			Pterocarpus santalinus L.f.	Leguminosae	Sandelträ
			Curcuma longa L.	Zingiberaceae	Gurkmeja
			Ocimum tenuiflorum L.	Lamiaceae	Rama Tulsi
			Azadirachta indica A.Juss.	Meliaceae	Neem
			Tinospora cordifolia (Willd.) Miers	Menispermaceae	Guduchi
67	4	Capsules	*Cyperus rotundus* L.	Cyperaceae	Motha
			Azadirachta indica A.Juss.	Meliaceae	Neem
			Curcuma longa L.	Zingiberaceae	Gurkmeja
			Ocimum tenuiflorum L.	Lamiaceae	Rama Tulsi
68	1	Capsules	*Glycyrrhiza glabra* L.	Leguminosae	Lakritsrot / Yashtimadhu
69	1	Capsules	*Saraca indica* L.	Leguminosae	
70	1	Capsules	*Aegle marmelos* (L.) Corrêa	Rutaceae	Bael
71	22	Tablets	Asphalatum $		Shilajit
			Withania somnifera (L.) Dunal	Solanaceae	Ashwagandha
			Gymnema sylvestre (Retz.) R.Br. ex Sm.	Apocynaceae	Gurmaar
			Azadirachta indica A.Juss.	Meliaceae	Nimba

			Terminalia chebula Retz.	Combretaceae	Harar choti
			Tinospora cordifolia (Willd.) Miers	Menispermaceae	Giloy
			Holarrhena antidysenterica (Roth) Wall. ex A.DC.(= *H. pubescens* Wall. ex G.Don)	Apocynaceae	Kutaj
			Tribulus terrestris L.	Zygophyllaceae	Gokhrudana
			Terminalia bellirica (Gaertn.) Roxb.	Combretaceae	Bahera
			Emblica officinalis Gaertn. (= *Phyllanthus emblica* L.)	Phyllanthaceae	Amala
			Aegle marmelos (L.) Corrêa	Rutaceae	Belpatra
			Curcuma zedoaria (Christm.) Roscoe	Zingiberaceae	Kachoor
			Justicia adhatoda L.	Acanthaceae	Vasa
			Ficus benghalensis L.	Moraceae	Badjata
			Acacia arabica (Lam.) Willd. (= *A. nilotica* (L.) Delile)	Leguminosae	Kikarfali
			Strychnos nux-vomica L.	Loganiaceae	Kuchla Shudh
			Centratherum anthelminticum (L.) Gamble (= *Baccharoides anthelmintica* (L.) Moench)	Compositae	Kaali jeeri
			Picrorhiza kurroa Royle ex Benth.	Plantaginaceae	Kutki
			Syzygium cumini (L.) Skeels	Myrtaceae	Jamun guthli
			Swertia chirata Buch.-Ham. ex Wall.	Gentianaceae	Chirayata
			Curcuma longa L.	Zingiberaceae	Haldi
			Trigonella foenum-graecum L.	Leguminosae	Methi
			Salacia chinensis L.	Celastraceae	Saptrangi
72	9	Tablets	*Bacopa monnieri* (L.) Wettst.	Plantaginaceae	
			Convolvulus pluricaulis Choisy (= *C. prostratus* Forssk.)	Convolvulaceae	
			Acorus calamus L.	Acoraceae	
			Onosma bracteatum Wall	Boraginaceae	
			Celastrus paniculatus Willd.	Celastraceae	
			Withania somnifera (L.) Dunal	Solanaceae	
			Tinospora cordifolia (Willd.) Miers	Menispermaceae	
			Praval pishti $		
			Mukta pishti $		
			Nardostachys jatamansi (D.Don) DC.	Caprifoliaceae	
			Rauvolfia serpentina (L.) Benth. ex Kurz	Apocynaceae	
73	14	Tablets	*Oryza sativa* L.	Poaceae	
			Saccharum munja Roxb. (= *Saccharum bengalense* Retz.)	Poaceae	
			Saccharum officinarum L.	Poaceae	
			Echinops echinatus Roxb.	Compositae	

			Tinospora cordifolia (Willd.) Miers	Menispermaceae	
			Premna mucronata Roxb. (= *Premna mollissima* Roth)	Lamiaceae	
			Cassia fistula L.	Leguminosae	
			Sida cordifolia L.	Malvaceae	
			Asparagus racemosus Willd.	Asparagaceae	
			Pueraria tuberosa (Willd.) DC.	Leguminosae	
			Solanum surattense Burm. f.	Solanaceae	
			Solanum indicum L.	Solanaceae	
			Hordeum vulgare L.	Poaceae	
			Picrorhiza kurroa Royle ex Benth.	Plantaginaceae	
74	9	Tablets	*Bacopa monnieri* (L.) Wettst.*	Plantaginaceae	
			Convolvulus pluricaulis Choisy (= *C. prostratus* Forssk.)*	Convolvulaceae	
			Acorus calamus L.*	Acoraceae	
			Lavandula stoechas L.	Lamiaceae	
			Onosma bracteatum Wall	Boraginaceae	
			Celastrus paniculatus Willd.*	Celastraceae	
			Nardostachys jatamansi (D.Don) DC.	Caprifoliaceae	
			Foeniculum vulgare Mill.	Apiaceae	
			Withania somnifera (L.) Dunal	Solanaceae	
			Corallium rubrum$		
			Mytillus margaretiferous $		
75	3	Tablets	*Tinospora cordifolia* (Willd.) Miers	Menispermaceae	Giloy
			Ocimum tenuiflorum L.	Lamiaceae	Tulsi
			Azadirachta indica A.Juss.	Meliaceae	Neem
76	1	Powder	*Withania somnifera* (L.) Dunal	Solanaceae	Ashwagandha pulver
77	1	Powder	*Asparagus racemosus* Willd.	Asparagaceae	Raw Shatavari root
78	1	Powder	*Tribulus terrestris* L.	Zygophyllaceae	Raw Gokshura pulver
79	1	Powder	*Terminalia arjuna* (Roxb. ex DC.) Wight & Arn.	Combretaceae	Raw Arjuna Pulver

Notes. Ayurvedic herbal products 1-26 were purchased from pharmacies and herbal shops in Romania, 27-47 from Norway, and 48-79 purchased via e-commerce from Sweden. $ Non-plant ingredients. * Scientific names that indicate the use of various refined/standardized herbal substances within the product; whereas, all others indicate the use of not extracted plant material, including simply processed and comminuted plant material, within the product. # These vernacular names indicate that these species have more scientific names, and Ayurvedic pharmacopoeia of India was used to choose the correct plant species name. All other plant ingredients listed on the product label, are provided with both the scientific and vernacular name.
