# Supplementary material for: DNA Metabarcoding Authentication of Ayurvedic Herbal Products on the European Market Raises Concerns of Quality and Fidelity
Source: Front Plant Sci. 2019 Feb 5;10:68. doi: 10.3389/fpls.2019.00068 (PMC6370972; doi:10.3389/fpls.2019.00068)
Supplement: Supplementary file 2 [file Data_Sheet_2.PDF]

Supplementary Table S2. Details of the plants species used as ingredients in the herbal products.

Scientific names as indicated on the product label*	Plant family	Habit	Source#	Conservation status#	nrITS sequences in GenBank (NCBI)$
Abutilon indicum (L.) Sweet	Malvaceae	Shrub	Wild	Least Concern	Yes
Acacia arabica (Lam.) Willd. (= *Acacia nilotica* (L.) Delile)	Leguminosae	Tree	Wild	Least Concern	Yes
Acacia catechu (L.f.) Willd.	Leguminosae	Tree	Wild	Least Concern	Yes
Achillea millefolium L.	Compositae	Herb	Wild/Cultivation	Least Concern	Yes
Achyranthes aspera L.	Amaranthaceae	Herb	Wild	Least Concern	Yes
Acorus calamus L.	Acoraceae	Herb	Wild/Cultivation	Least Concern	Yes
Aegle marmelos (L.) Corrêa	Rutaceae	Tree	Wild	Vulnerable	Yes
Albizia lebbeck (L.) Benth.	Leguminosae	Tree	Wild	Least Concern	Genus is represented
Allium sativum L.	Amaryllidaceae	Herb	Cultivation	Least Concern	Yes
Aloe barbadensis Mill. (= *Aloe vera* (L.) Burm.f.)	Xanthorrhoeaceae	Herb	Cultivation	Least Concern	Yes
Alpinia galanga (L.) Willd.	Zingiberaceae	Herb	Wild/Cultivation	Least Concern	Yes
Althaea officinalis L.	Malvaceae	Herb	Wild	Least Concern	Yes
Amomum subulatum Roxb.	Zingiberaceae	Herb	Cultivation	Least Concern	Yes
Andrographis paniculata (Burm.f.) Nees	Acanthaceae	Herb	Wild/Cultivation	Least Concern	Yes
Anethum sowa Roxb. ex Fleming	Apiaceae	Herb	Cultivation	Least Concern	Genus is represented
Apium graveolens L.	Apiaceae	Herb	Cultivation	Least Concern	Yes
Asparagus racemosus Willd.	Asparagaceae	Shrub	Wild/Cultivation	Least Concern	Yes
Asteracantha longifolia Nees (= *Hygrophila auriculata* (Schumach.) Heine)	Acanthaceae	Herb	Wild	Least Concern	Yes
Azadirachta indica A.Juss.	Meliaceae	Tree	Wild/Cultivation	Least Concern	Yes
Bacopa monnieri (L.) Wettst.	Plantaginaceae	Herb	Wild/Cultivation	Least Concern	Yes
Bauhinia variegata L.	Leguminosae	Tree	Wild/Cultivation	Least Concern	Yes
Berberis aristata DC.	Berberidaceae	Tree	Wild	Vulnerable	Yes
Bergenia ligulata Engl.	Saxifragaceae	Herb	Wild	Least Concern	Yes
Boerhaavia diffusa L.	Nyctaginaceae	Herb	Wild	Least Concern	Yes
Bombax ceiba L.	Malvaceae	Tree	Wild	Least Concern	Yes
Boswellia serrata Roxb. ex Colebr.	Burseraceae	Tree	Wild	Vulnerable	Genus is represented

Bougainvillea spectabilis Willd.	Nyctaginaceae	Woody Vine	Wild/Cultivation	Least Concern	Yes
Caesalpinia bonducella (L.) Fleming (= *Caesalpinia bonduc* (L.) Roxb.)	Leguminosae	Herb	Wild/Cultivation	Least Concern	Yes
Camellia sinensis (L.) Kuntze	Theaceae	Shrub	Cultivation	Least Concern	Yes
Capparis spinosa L.	Capparaceae	Shrub	Wild/Cultivation	Least Concern	Yes
Carica papaya L.	Caricaceae	Tree	Cultivation	Least Concern	Yes
Carum copticum (L.) Benth. & Hook.f. ex C.B.Clarke (= *Trachyspermum ammi* (L.) Sprague)	Apiaceae	Herb	Cultivation	Least Concern	Yes
Casearia esculenta Roxb.	Salicaceae	Shrub	Wild	Least Concern	Genus is represented
Cassia fistula L.	Leguminosae	Tree	Wild/Cultivation	Least Concern	Yes
Cassia occidentalis L. (=of *Senna occidentalis* (L.) Link)	Leguminosae	Herb	Wild	Least Concern	Yes
Celastrus paniculatus Willd.	Celastraceae	Woody Liana	Wild/Cultivation	Vulnerable	Yes
Centella asiatica (L.) Urb.	Apiaceae	Herb	Wild/Cultivation	Least Concern	Yes
Centratherum anthelminticum (L.) Gamble (= *Baccharoides anthelmintica* (L.) Moench)	Compositae	Herb	Wild/Cultivation	Least Concern	Yes
Chlorophytum arundinaceum Baker	Asparagaceae	Herb	Wild	Endangered	Yes
Cichorium intybus L.	Compositae	Herb	Wild/Cultivation	Least Concern	Yes
Cinnamomum cassia (L.) J.Presl	Lauraceae	Tree	Import	Least Concern	Yes
Cinnamomum zeylanicum Blume (= *Cinnamomum verum* J.Presl)	Lauraceae	Tree	Wild/Cultivation	Least Concern	Yes
Cissus quadrangularis L.	Vitaceae	Vine	Wild/Cultivation	Least Concern	Yes
Citrus limon (L.) Osbeck	Rutaceae	Tree	Cultivation	Least Concern	Yes
Coccinia indica Wight & Arn. (= *Coccinia grandis* (L.) Voigt)	Cucurbitaceae	Vine	Cultivation	Least Concern	Yes
Commiphora mukul (Hook. ex Stocks) Engl.	Burseraceae	Shrub	Wild	Least Concern	Yes
Commiphora wightii (Arn.) Bhandari	Burseraceae	Shrub	Wild	Endangered	Yes
Convolvulus pluricaulis Choisy (= *Convolvulus prostratus* Forssk.)	Convolvulaceae	Herb	Wild	Least Concern	Yes
Crateva nurvala Buch.-Ham	Capparaceae	Tree	Wild/Cultivation	Least Concern	Genus is represented
Crocus sativus L.	Iridaceae	Herb	Cultivation	Least Concern	Yes
Curcuma longa L.	Zingiberaceae	Herb	Cultivation	Least Concern	Yes
Curcuma zedoaria (Christm.) Roscoe	Zingiberaceae	Herb	Wild/Cultivation	Least Concern	Yes

Cyperus rotundus L.	Cyperaceae	Herb	Wild	Least Concern	Yes
Cyperus scariosus R.Br.	Cyperaceae	Herb	Wild	Least Concern	Genus is represented
Desmodium gangeticum (L.) DC.	Leguminosae	Herb	Wild	Least Concern	Yes
Didymocarpus pedicellata (Ait.) Ait. F.	Asclepiadaceae	Herb	Wild	Endangered	Genus is represented
Echinops echinatus Roxb.	Compositae	Herb	Wild	Least Concern	Yes
Eclipta alba (L.) Hassk. (= *Eclipta prostrata* (L.) L.)	Compositae	Herb	Wild	Least Concern	Yes
Elettaria cardamomum (L.) Maton	Zingiberaceae	Herb	Cultivation	Least Concern	Yes
Embelia ribes Burm.f.	Primulaceae	Woody Climber	Wild	Vulnerable	Yes
Enicostemma littorale Blume	Gentianacae	Herb	Wild	Least Concern	No (other genera are represented)
Evolvulus alsinoides (L.) L.	Convolvulaceae	Herb	Wild	Least Concern	Yes
Ficus benghalensis L.	Moraceae	Tree	Wild	Least Concern	Yes
Foeniculum vulgare Mill.	Apiaceae	Herb	Cultivation	Least Concern	Yes
Fumaria officinalis L.	Papaveraceae	Herb	Wild/Cultivation	Least Concern	Yes
Garcinia cambogia (Gaertn.) Desr. (= *Garcinia gummi-gutta* (L.) Roxb.)	Clusiaceae	Tree	Wild/Cultivation	Least Concern	Yes
Garcinia indica (Thouars) Choisy	Clusiaceae	Tree	Wild/Cultivation	Least Concern	Yes
Glycyrrhiza glabra L.	Leguminosae	Herb	Cultivation	Least Concern	Yes
Gmelina arborea Roxb.	Lamiaceae	Tree	Wild	Least Concern	Yes
Gossypium herbaceum L.	Malvaceae	Shrub	Cultivation	Least Concern	Yes
Gymnema sylvestre (Retz.) R.Br. ex Sm.	Apocynaceae	Herb	Wild	Vulnerable	Yes
Hemidesmus indicus (L.) R. Br. ex Schult.	Apocynaceae	Herb	Wild	Least Concern	Yes
Hibiscus rosa-sinensis L.	Malvaceae	Shrub	Cultivation	Least Concern	Yes
Holarrhena antidysenterica (Roth) Wall. ex A.DC.(= *Holarrhena pubescens* Wall. ex G.Don)	Apocynaceae	Tree	Wild	Least Concern	Yes
Hordeum vulgare L.	Poaceae	Herb	Cultivation	Least Concern	Yes
Humulus lupulus L.	Cannabaceae	Climber	Cultivation	Least Concern	Yes
Hyssopus officinalis L.	Lamiaceae	Herb	Wild	Vulnerable	Yes
Inula racemosa Hook.f.	Compositae	Herb	Wild	Least Concern	Yes
Ipomoea digitata L. (= *Ipomoea cheirophylla* O'Donell)	Convolvulaceae	Herb	Wild	Least Concern	Genus is represented
Iris germanica L.	Iridaceae	Herb	Import/Cultivation	Least Concern	Yes
Justicia adhatoda L.	Acanthaceae	Shrub	Wild/Cultivation	Least Concern	Yes

Kaempferia galanga L.	Zingiberaceae	Herb	Cultivation	Least Concern	Yes
Lavandula stoechas L.	Lamiaceae	Shrub	Import	Least Concern	Yes
Lepidium sativum L.	Brassicaceae	Herb	Cultivation	Least Concern	Yes
Leptadenia reticulata (Retz.) Wight & Arn.	Apocynaceae	Shrub	Wild	Endangered	Yes
Lotus arabicus L.	Leguminosae	Herb	Wild	Least Concern	Yes
Mentha piperita L.	Lamiaceae	Herb	Cultivation	Least Concern	Yes
Mentha arvensis L.	Lamiaceae	Herb	Wild/Cultivation	Least Concern	Yes
Momordica charantia L.	Cucurbitaceae	Vine	Cultivation	Least Concern	Yes
Moringa oleifera Lam.	Moringaceae	Tree	Cultivation	Least Concern	Yes
Moringa pterygosperma Gaertn.	Moringaceae	Tree	Cultivation	Least Concern	Yes
Mucuna pruriens (L.) DC.	Leguminosae	Climber	Wild	Endangered	Yes
Myristica fragrans Houtt.	Myristicaceae	Tree	Wild/Cultivation	Least Concern	Yes
Nardostachys jatamansi (D.Don) DC.	Caprifoliaceae	Herb	Wild	Vulnerable	Yes
Ocimum gratissimum L.	Lamiaceae	Herb	Wild/Cultivation	Least Concern	Yes
Ocimum tenuiflorum L.	Lamiaceae	Herb	Cultivation	Least Concern	Yes
Onosma bracteatum Wall	Boraginaceae	Herb	Wild	Least Concern	Genus is represented
Oroxylum indicum (L.) Kurz	Bignoniaceae	Tree	Wild	Vulnerable	Yes
Oryza sativa L.	Poaceae	Herb	Cultivation	Least Concern	Yes
Phyllanthus amarus Schumach. & Thonn.	Phyllanthaceae	Herb	Wild	Least Concern	Yes
Phyllanthus emblica L.	Phyllanthaceae	Tree	Wild/Cultivation	Vulnerable	Yes
Phyllanthus niruri L.	Phyllanthaceae	Herb	Wild	Least Concern	Yes
Phyllanthus urinaria L.	Phyllanthaceae	Herb	Wild	Least Concern	Yes
Picrorhiza kurroa Royle ex Benth.	Plantaginaceae	Herb	Wild	Endangered	Yes
Piper chaba Hunter (= *P. retrofractum* Vahl)	Piperaceae	Vine	Cultivation	Least Concern	Yes
Piper cubeba Bojer	Piperaceae	Vine	Cultivation	Least Concern	Yes
Piper longum L.	Piperaceae	Vine	Cultivation	Least Concern	Yes
Piper nigrum L.	Piperaceae	Vine	Cultivation	Least Concern	Yes
Plantago ovata Forssk.	Plantaginaceae	Herb	Cultivation	Least Concern	Yes
Plumbago zeylanica L.	Plumbaginaceae	Climber	Wild	Vulnerable	Yes
Premna integrifolia Willd. (= *P. serratifolia* L.)	Lamiaceae	Shrub	Wild	Least Concern	Yes
Premna mucronata Roxb. (= *P. mollissima* Roth)	Lamiaceae	Shrub	Wild	Least Concern	Yes
Prunus amygdalus Batsch (= *P. dulcis* (Mill.) D.A.Webb)	Rosaceae	Tree	Wild	Least Concern	Yes
Pterocarpus marsupium Roxb.	Leguminosae	Tree	Wild	Endangered	Yes

Pterocarpus santalinus L.f.	Leguminosae	Tree	Wild	Endangered	Yes
Pueraria tuberosa (Willd.) DC.	Leguminosae	Climber	Wild	Vulnerable	Yes
Raphanus sativus L. (= *Raphanus raphanistrum* subsp. *sativus* (L.) Domin)	Brassicaceae	Herb	Cultivation	Least Concern	Yes
Rauvolfia serpentina (L.) Benth. ex Kurz	Apocynaceae	Shrub	Wild	Endangered	Yes
Rheum emodi L.	Polygonaceae	Herb	Wild	Endangered	Yes
Rubia cordifolia L.	Rubiaceae	Climber	Wild	Vulnerable	Yes
Rumex maritimus L.	Polygonaceae	Herb	Wild	Least Concern	Genus is represented
Saccharum munja Roxb. (= *Saccharum bengalense* Retz.)	Poaceae	Shrub	Wild/Cultivation	Least Concern	Yes
Saccharum officinarum L.	Poaceae	Shrub	Cultivation	Least Concern	Yes
Salacia chinensis L.	Celastraceae	Climber	Wild	Least Concern	Yes
Santalum album L.	Santalaceae	Tree	Wild	Endangered	Yes
Sapindus trifoliatus L.	Sapindaceae	Tree	Wild	Least Concern	Yes
Saraca asoca (Roxb.) Willd.	Leguminosae	Tree	Wild	Endangered	Yes
Saraca indica L.	Leguminosae	Tree	Wild	Least Concern	Yes
Saxifraga granulata L.	Saxifragaceae	Herb	Wild	Least Concern	Yes
Saxifraga ligulata Murray (= *Saxifraga stolonifera* Curtis)	Saxifragaceae	Herb	Wild	Least Concern	Yes
Sida cordifolia L.	Malvaceae	Herb	Wild	Least Concern	Yes
Solanum indicum L.	Solanaceae	Herb	Wild	Least Concern	Yes
Solanum nigrum L.	Solanaceae	Herb	Wild	Least Concern	Yes
Solanum surattense Burm. f.	Solanaceae	Herb	Wild	Least Concern	Yes
Solanum xanthocarpum Schrad. & H. Wendl. (= *Solanum virginianum* L.)	Solanaceae	Herb	Wild	Least Concern	Yes
Sphaeranthus indicus L.	Compositae	Herb	Wild	Least Concern	Yes
Stereospermum suaveolens (Roxb.) DC. (= *Stereospermum chelonoides* (L.f.) DC.)	Bignoniaceae	Tree	Wild	Vulnerable	Yes
Strychnos nux-vomica L.	Loganiaceae	Tree	Wild	Vulnerable	Yes
Swertia chirata Buch.-Ham. ex Wall.	Gentianaceae	Herb	Wild	Endangered	Yes
Symplocos racemosa Roxb.	Symplocaceae	Shrub	Wild	Vulnerable	Yes
Syzygium cumini (L.) Skeels	Myrtaceae	Tree	Wild/Cultivation	Least Concern	Yes
Tamarix gallica L	Tamaricaceae	Shrub	Wild	Least Concern	Yes
Tephrosia purpurea (L.) Pers.	Leguminosae	Herb	Wild/Cultivation	Least Concern	Yes
Terminalia arjuna (Roxb. ex DC.) Wight & Arn.	Combretaceae	Tree	Wild	Least Concern	Yes

Terminalia bellirica (Gaertn.) Roxb.	Combretaceae	Tree	Wild	Least Concern	Yes
Terminalia chebula Retz.	Combretaceae	Tree	Wild	Least Concern	Yes
Tinospora cordifolia (Willd.) Miers	Menispermaceae	Climber	Wild/Cultivation	Least Concern	Yes
Tribulus terrestris L.	Zygophyllaceae	Herb	Wild	Least Concern	Yes
Trigonella foenum-graecum L.	Leguminosae	Herb	Cultivation	Least Concern	Yes
Uraria picta (Jacq.) DC.	Leguminosae	Herb	Wild	Endangered	Yes
Valeriana officinalis L.	Caprifoliaceae	Herb	Wild	Least Concern	Yes
Valeriana wallichii DC. (= *Valeriana jatamansi* Jones)	Caprifoliaceae	Herb	Wild	Vulnerable	Yes
Vernonia cinerea (L.) Less. (= *Cyanthillium cinereum* (L.) H.Rob.)	Compositae	Herb	Wild	Least Concern	Yes
Vinca rosea L. (= *Catharanthus roseus* (L.) G.Don)	Apocynaceae	Herb	Cultivation	Least Concern	Yes
Viola odorata L.	Violaceae	Herb	Cultivation	Least Concern	Yes
Vitis vinifera L.	Vitaceae	Climber	Cultivation	Least Concern	Yes
Withania somnifera (L.) Dunal	Solanaceae	Shrub	Wild/Cultivation	Least Concern	Yes
Zingiber officinale Roscoe	Zingiberaceae	Herb	Cultivation	Least Concern	Yes
Ziziphus sativa Gaertn. (= *Ziziphus jujuba* Mill.)	Rhamnaceae	Tree	Wild/Cultivation	Least Concern	Yes

Notes. *Scientific names as indicated on the product label, whereas scientific names within the parenthesis are the accepted names according to the Plant List (http://www.theplantlist.org/). $Plant source and conservation status was categorized based on the database ENVIS centre for medicinal plants, National Medicinal Plants Board, India (http://envis.frlht.org/), and from the reports by published by National Medicinal Plants Board, Ministry of AYUSH, Government of India (Demand and supply of medicinal plants in India, 2007; and Medicinal Plants in India: An Assessment of their Demand and Supply, 2017). # The availability of nrITS sequences in Genbank was checked on July 27, 2017.
