# Supplementary material for: DNA Metabarcoding Authentication of Ayurvedic Herbal Products on the European Market Raises Concerns of Quality and Fidelity
Source: Front Plant Sci. 2019 Feb 5;10:68. doi: 10.3389/fpls.2019.00068 (PMC6370972; doi:10.3389/fpls.2019.00068)
Supplement: Supplementary file 3 [file Data_Sheet_3.PDF]

Supplementary Table S3. Details of total number sequencing reads per products.

Herbal product ID		1	2	3	4	5	6	7	8	9	10	12	13	14	15	16	17	18	19	23	24	25	26	27	30	31	32	34	36	40	52	55	58	60	61	63	66	68	69	70	73	74	75	77	79		
**Name of the species detected in the herbal products using DNA metabarcoding**	**Family**	**Total number of nrITS1 and nrITS2 reads (Replicate 1+2+3)**																																												**No. of occurrences**	**Occurrence in the 44 products (%)**
Abelmoschus esculentus (L.) Moench	Malvaceae	0	0	0	0	0	0	0	0	0	0	0	0	0	0	0	0	0	0	0	0	0	0	0	0	0	0	0	0	0	0	0	0	0	0	0	0	0	0	0	126	0	0	0	0	1	2 %
Abutilon indicum (L.) Sweet	Malvaceae	0	0	0	0	0	0	0	0	0	0	0	0	0	0	0	0	0	117	0	0	0	0	0	0	0	0	0	0	0	0	0	0	0	0	0	0	0	0	0	0	0	0	0	0	1	2 %
Acanthospermum hispidum DC.	Compositae	0	0	0	0	0	0	0	0	0	0	0	0	0	0	205	0	0	0	0	0	0	0	0	0	0	0	0	0	0	0	0	0	0	0	0	0	0	0	0	0	0	0	0	0	1	2 %
Agrimonia eupatoria L.	Rosaceae	0	0	0	0	0	0	0	0	0	0	0	0	0	50	0	0	0	0	0	0	0	0	0	0	0	0	0	0	0	0	0	0	0	0	0	0	0	0	0	0	0	0	0	0	1	2 %
Alcea rosea L.	Malvaceae	0	0	0	0	0	0	0	0	0	0	0	0	0	0	0	0	0	0	0	0	0	0	0	0	0	274	0	0	0	0	0	0	0	0	0	0	0	0	0	0	0	0	0	0	1	2 %
Allocasuarina zephyrea L.A.S.Johnson	Casuarinaceae	0	0	0	0	0	0	0	0	0	0	0	0	0	0	0	0	0	0	0	0	0	1685	0	0	0	0	0	0	0	0	0	0	0	0	0	0	0	0	0	0	0	0	0	0	1	2 %
Allophylus bojerianus (Cambess.) Blume	Sapindaceae	0	0	0	0	0	0	0	0	0	0	0	0	0	0	0	0	0	0	0	0	0	0	0	0	0	0	0	0	0	0	0	0	0	0	0	0	0	44	0	0	0	0	0	0	1	2 %
Althaea officinalis L.	Malvaceae	0	0	0	0	0	0	0	0	0	0	0	0	0	0	0	2632	2684	0	0	0	0	0	0	10418	0	0	0	0	0	0	0	0	0	111	0	0	384	2665	0	67	0	0	0	800	8	18 %
Amaranthus viridis L.	Amaranthaceae	0	0	0	0	0	0	0	0	0	0	0	0	0	0	0	0	0	0	0	0	0	0	0	0	0	0	0	0	0	12	0	0	0	0	0	0	0	0	0	0	0	0	0	0	1	2 %
Ammi trifoliatum (H.C.Watson) Trel.	Apiaceae	0	0	0	0	0	0	0	0	0	0	0	0	0	0	0	0	0	0	0	0	0	0	11	0	0	0	0	0	0	0	0	0	0	0	0	0	0	0	0	0	0	0	0	0	1	2 %
Anchusa italica Retz.	Boraginaceae	0	0	0	0	0	0	0	0	0	230	0	0	0	0	0	0	0	0	0	0	0	0	0	0	0	0	0	0	0	0	0	0	0	0	0	0	0	0	0	0	0	0	0	0	1	2 %
Apera spica-venti (L.) P.Beauv.	Poaceae	0	0	0	0	0	0	0	0	0	0	0	0	0	0	0	0	0	0	0	0	0	93	0	0	0	0	0	0	0	0	0	0	0	0	0	0	0	0	0	0	0	0	0	0	1	2 %
Arachis correntina (Burkart) Krapov. & W.C.Greg.	Leguminosae	0	0	0	0	0	0	0	0	0	0	0	0	0	0	0	0	0	0	0	0	0	0	0	0	0	0	0	0	0	0	0	0	0	0	0	0	0	0	0	12	0	0	0	0	1	2 %
Asparagus racemosus Willd.	Asparagaceae	0	0	0	0	0	0	23	0	0	0	0	0	0	0	0	0	0	0	0	0	0	0	0	0	0	0	0	0	0	0	0	0	0	0	0	0	0	0	0	0	0	0	2457	0	2	5 %
Azadirachta indica A.Juss.	Meliaceae	0	0	51	0	0	0	0	0	0	0	0	0	0	0	0	0	0	0	0	0	0	0	0	0	0	0	0	0	0	0	0	0	0	0	0	120	0	0	0	0	0	155	0	0	3	7 %
Baccharoides anthelmintica (L.) Moench	Asteraceae	0	0	0	0	0	0	0	0	0	0	0	0	0	0	0	0	0	0	0	0	0	0	0	0	0	0	0	0	0	0	0	0	0	2769	0	0	0	2903	109795	69783	0	0	0	0	4	9 %
Behnia reticulata (Thunb.) Didr.	Asparagaceae	0	0	0	0	0	0	0	0	0	0	0	0	0	0	0	0	0	0	0	0	0	0	0	0	0	0	0	0	0	0	0	0	0	0	0	0	0	0	0	0	0	0	4004	0	1	2 %
Berberis asiatica Roxb. ex DC.	Berberidaceae	0	0	0	0	0	0	0	0	0	0	0	0	0	0	0	0	0	0	0	0	0	0	0	0	0	85	0	0	0	0	0	0	0	0	0	0	0	0	0	0	0	0	0	0	1	2 %
Bergenia ligulata Engl.	Saxifragaceae	0	0	0	0	0	0	0	0	0	0	0	0	0	0	2657	0	0	0	0	0	0	0	0	0	0	0	0	0	0	0	0	0	0	0	0	0	0	0	0	0	0	0	0	0	1	2 %
Boerhavia diffusa L.	Nyctaginaceae	0	0	0	0	0	0	0	0	0	0	0	0	0	0	0	0	0	0	0	0	0	0	17	0	0	0	225	0	169	0	0	0	0	1444	0	0	0	0	203	320	21	0	0	0	7	16 %
Brassica nigra (L.) K.Koch	Brassicaceae	0	0	17	0	0	0	0	0	0	0	0	0	0	0	0	0	0	0	0	0	0	0	0	0	0	0	0	0	0	0	0	0	0	0	0	0	0	0	0	0	0	0	0	0	1	2 %
Brassica oleracea L.	Brassicaceae	0	0	0	0	0	0	0	0	0	0	0	0	21278	0	0	0	0	0	0	0	0	0	43	4487	0	0	0	0	0	0	0	0	0	0	0	0	0	0	0	0	0	0	0	0	3	7 %
Calluna vulgaris (L.) Hull	Ericaceae	0	0	0	0	0	0	503	0	0	0	0	0	0	0	0	0	0	0	0	0	0	0	0	0	0	0	0	0	0	0	0	0	0	0	0	0	0	0	0	0	0	0	0	0	1	2 %
Cannabis sativa L.	Cannabaceae	0	0	0	0	0	0	0	0	43786	0	0	0	0	0	0	0	0	0	0	0	0	0	0	0	0	0	0	0	0	0	0	0	0	0	0	0	0	0	0	0	0	0	0	0	1	2 %
Cassia sp.	Leguminosae	31	0	0	0	0	0	0	0	18	0	0	0	0	0	0	0	0	0	0	0	0	0	0	0	0	0	0	0	0	0	0	0	0	0	0	0	0	0	0	0	0	0	0	0	2	5 %
Celastrus paniculatus Willd.	Celastraceae	0	0	0	0	0	0	0	0	0	0	0	0	0	0	0	0	0	0	0	0	0	0	0	0	0	0	0	0	0	0	0	0	0	0	0	0	0	0	0	0	164	0	0	0	1	2 %
Cenchrus americanus (L.) Morrone	Poaceae	0	0	0	0	0	0	0	0	0	0	0	0	0	0	0	0	0	0	0	0	0	0	0	0	0	0	0	0	0	0	0	0	0	0	0	0	0	0	0	6229	0	0	0	0	1	2 %
Centaurea cyanus L.	Compositae	0	0	0	0	0	0	0	0	0	0	0	0	0	0	0	0	0	0	61	0	0	0	0	0	0	0	0	0	0	0	0	0	0	0	0	0	0	0	0	0	0	0	0	0	1	2 %
Centella asiatica (L.) Urb.	Apiaceae	0	0	0	0	0	0	0	0	0	0	0	0	0	0	0	32	0	0	0	0	0	0	38	0	0	692	0	0	0	0	0	0	0	0	0	0	0	0	0	0	362	0	0	0	4	9 %
Chaerophyllum reflexum Aitch.	Apiaceae	0	0	0	0	0	0	0	0	0	0	0	0	0	0	0	0	0	0	0	0	0	0	49	0	0	0	0	0	0	0	0	0	0	0	0	0	0	0	0	0	0	0	0	0	1	2 %
Chamaecrista olesiphylla (Vogel) H.S.Irwin & Barneby	Leguminosae	248	0	0	0	0	0	0	0	0	0	0	0	0	0	0	0	0	0	0	0	0	0	64	0	0	0	0	0	0	0	0	0	0	0	0	0	0	0	0	0	0	0	0	0	2	5 %
Cichorium intybus L.	Compositae	0	0	592	0	0	819	37	0	476	0	0	2334	0	0	0	0	0	0	0	0	0	0	0	0	0	0	0	0	0	0	0	0	0	0	0	0	89	105	0	0	0	0	0	0	7	16 %
Cinnamomum cassia (L.) J.Presl	Lauraceae	0	0	0	0	0	0	0	0	0	0	0	0	0	0	0	0	0	0	0	0	0	0	0	0	0	0	2525	0	0	0	0	0	0	0	0	0	0	0	0	0	0	0	0	0	1	2 %
Cinnamomum iners Reinw. ex Blume	Lauraceae	0	0	0	0	0	59	0	0	0	0	0	0	0	0	0	0	0	0	0	0	0	0	0	0	0	0	0	0	0	0	0	0	0	0	0	0	0	0	0	0	0	0	0	0	1	2 %
Cissus quadrangularis L.	Vitaceae	0	0	0	0	0	0	0	0	0	0	0	0	0	0	0	0	0	0	0	0	0	0	0	0	0	0	0	0	54	0	0	0	0	0	0	0	0	0	0	0	0	0	0	0	1	2 %
Citrullus lanatus (Thunb.) Matsum. & Nakai	Cucurbitaceae	0	0	0	0	0	0	0	0	0	0	0	0	0	0	0	0	0	0	0	0	0	0	0	0	0	0	0	0	0	0	0	0	0	0	0	0	0	0	0	18	0	0	0	0	1	2 %
Citrus clementina hort.	Rutaceae	0	0	0	0	0	0	0	0	0	0	0	0	0	0	0	0	0	0	50	0	0	0	0	0	0	0	0	0	0	0	0	0	0	0	0	0	0	0	0	0	0	0	0	0	1	2 %
Coccinia adoensis (Hochst. ex A. Rich.) Cogn.	Cucurbitaceae	0	0	0	0	0	0	0	0	0	0	0	0	0	0	0	0	0	0	0	0	0	0	0	0	0	0	0	0	0	0	0	43	0	0	0	0	0	0	0	0	0	0	0	0	1	2 %
Coccinia grandis (L.) Voigt	Cucurbitaceae	0	0	0	0	0	0	0	0	0	0	0	0	0	0	0	0	0	0	0	0	0	0	0	0	0	0	0	0	0	0	0	588	0	0	66	0	0	0	0	0	0	0	0	0	2	5 %
Coccinia sessilifolia (Sond.) Cogn.	Cucurbitaceae	0	0	0	0	0	0	11	0	0	0	0	0	0	0	0	0	0	0	0	0	0	0	69	0	0	0	0	0	0	0	0	0	0	0	0	0	0	0	0	0	0	0	0	0	2	5 %
Combretum nigricans Lepr. ex Guill. & Perr.	Combretaceae	0	0	0	0	0	0	0	0	0	0	0	0	0	0	0	0	0	0	0	0	0	0	0	0	0	0	0	0	0	0	0	0	0	0	0	0	0	0	0	148	0	0	0	0	1	2 %
Convolvulus microphyllus Sieber ex Spreng.	Convolvulaceae	0	0	0	0	0	0	0	0	0	1612	0	0	0	0	0	0	0	0	0	0	0	0	0	0	0	17	771	0	0	0	0	0	0	0	0	0	0	0	0	0	0	0	0	0	3	7 %
Convolvulus prostratus Forssk.	Convolvulaceae	64	0	0	0	0	0	0	0	0	24183	0	675	0	0	0	0	0	0	0	0	0	0	10	0	18	39	719	0	0	0	0	0	0	0	0	0	0	0	0	526	42942	0	12	0	10	23 %
Convolvulus subhirsutus Regel & Schmalh.	Convolvulaceae	0	0	0	0	0	0	0	0	0	0	0	0	0	0	0	0	0	0	0	23	0	0	0	0	0	0	292	0	0	0	0	0	0	0	0	0	0	0	0	0	0	0	0	0	2	5 %
Corchorus olitorius L.	Malvaceae	0	0	0	0	0	0	0	0	0	0	0	0	0	0	0	0	0	0	0	0	0	0	0	0	0	39	0	0	0	0	0	0	0	0	0	0	0	0	0	0	0	0	0	0	1	2 %
Coriandrum sativum L.	Apiaceae	75	0	0	0	0	87	0	0	0	0	0	0	504	0	0	0	0	0	0	14	0	0	131	0	0	60	28	0	0	0	0	0	0	0	0	0	225	89	0	26	0	0	0	0	10	23 %
Cucumis melo L.	Cucurbitaceae	0	0	0	0	0	0	0	0	0	0	0	0	0	0	455	0	0	0	74	0	0	0	0	0	0	0	0	0	0	0	0	0	0	0	0	0	0	0	0	0	43	0	0	0	3	7 %
Cucumis sativus L.	Cucurbitaceae	0	0	0	0	0	0	0	0	13	0	0	0	0	23	0	0	754	0	20	23	0	0	0	0	0	0	0	0	0	0	0	0	0	17316	0	0	0	0	100	0	0	0	0	0	7	16 %
Cullen corylifolium (L.) Medik.	Leguminosae	0	0	34	0	0	0	0	0	0	0	0	0	0	0	0	0	0	0	0	0	0	0	0	0	0	0	0	0	0	0	0	0	0	0	0	0	0	0	0	12	0	0	0	0	2	5 %
Cuminum cyminum L.	Apiaceae	400	0	0	0	0	59	0	0	0	23	0	0	0	0	0	0	0	0	0	0	36	0	220	0	0	1049	43	0	0	0	0	0	0	88	0	0	0	0	0	0	0	0	0	0	8	18 %
Cyamopsis tetragonoloba (L.) Taub.	Leguminosae	0	0	0	0	0	0	0	0	0	0	0	0	0	0	0	0	0	0	0	0	0	0	0	0	0	0	0	0	0	0	0	0	0	0	0	0	0	0	0	0	0	0	0	41	1	2 %
Cynanchum staubii Bosser	Apocynaceae	0	0	0	0	0	0	0	0	0	0	0	0	0	0	0	0	0	0	0	0	0	0	56	0	0	0	0	0	0	0	0	0	0	0	0	0	0	0	0	0	0	0	0	0	1	2 %
Cynodon dactylon (L.) Pers.	Poaceae	0	0	0	0	0	0	0	0	0	0	0	0	0	0	0	0	68	0	0	0	0	0	0	0	0	0	0	0	0	0	0	0	0	0	0	0	0	0	0	0	0	0	0	0	1	2 %
Damrongia cyanantha Triboun	Gesneriaceae	0	0	0	0	0	0	0	0	0	0	0	0	0	0	0	0	0	0	0	0	0	0	0	0	0	0	0	0	431	0	0	0	0	0	0	0	0	0	0	0	0	0	0	0	1	2 %
Doniophyton weddellii Katinas & Stuessy	Compositae	0	0	0	0	0	0	0	0	0	0	0	0	0	0	0	0	0	0	0	0	0	0	0	0	0	0	0	0	0	0	0	0	0	1865	0	0	0	0	0	0	0	0	0	0	1	2 %
Draba nivalis Lilj.	Brassicaceae	0	0	0	0	0	0	0	0	0	0	0	0	0	0	0	5060	0	0	0	0	0	0	0	0	0	0	0	0	0	0	0	0	0	4329	0	0	0	0	0	0	0	0	0	0	2	5 %
Drymocallis rupestris (L.) Soj k	Rosaceae	0	0	0	0	0	0	0	0	0	0	0	0	0	0	0	899	4591	135	0	0	0	0	0	0	0	0	0	0	0	0	0	0	0	0	0	0	0	0	0	0	0	0	0	0	3	7 %
Drypetes hoaensis Gagnep.	Putranjivaceae	0	0	0	0	0	0	0	0	0	0	0	0	0	0	0	0	0	0	0	0	0	0	0	0	0	0	0	0	904	0	0	0	0	0	0	0	0	0	0	0	0	0	0	0	1	2 %
Dyschoriste siphonantha (Nees) Kuntze	Acanthaceae	0	0	0	0	0	0	0	0	0	0	0	0	0	0	0	0	0	0	0	0	0	0	0	0	0	0	0	0	0	0	0	0	0	0	0	0	286	0	0	0	0	0	0	0	1	2 %
Echinacea purpurea (L.) Moench	Compositae	0	0	0	0	0	0	0	0	83	0	0	150	0	0	0	0	0	0	0	0	0	0	0	0	0	0	0	0	0	0	0	0	0	0	0	0	0	0	0	0	0	0	0	0	2	5 %
Echinochloa colona (L.) Link	Poaceae	0	0	0	0	0	0	0	0	0	0	0	0	0	0	0	0	0	0	0	0	0	0	0	0	0	0	0	0	0	0	0	0	0	0	1313	0	0	0	0	0	0	0	0	0	1	2 %
Echinochloa pyramidalis (Lam.) Hitchc. & Chase	Poaceae	0	0	0	0	0	0	0	0	0	0	0	0	0	0	0	0	0	0	0	0	0	0	0	0	0	0	0	0	0	0	0	0	0	0	72	0	0	0	0	0	0	0	0	0	1	2 %
Echium vulgare L.	Boraginaceae	0	0	0	0	0	25	0	0	0	0	0	0	0	0	0	0	0	0	0	0	0	0	0	0	0	0	0	0	0	0	0	0	0	0	0	0	0	0	0	0	0	0	0	0	1	2 %
Eclipta alba (L.) Hassk. *(syn. Eclipta prostrata L.)*	Compositae	0	0	0	0	0	0	0	0	0	0	0	110	0	0	0	0	0	0	0	0	0	0	0	0	0	0	0	0	0	86853	0	0	77	18404	0	0	16	150	0	0	0	0	0	0	6	14 %
Eleutherococcus sessiliflorus (Rupr. & Maxim.) S.Y.Hu	Araliaceae	0	0	0	0	0	0	0	0	0	0	0	0	0	0	0	0	0	0	0	0	0	0	0	0	0	0	0	0	0	0	0	0	0	0	0	0	0	0	205	0	62	0	0	0	2	5 %
Embelia tsjeriam-cottam (Roem. & Schult.) A.DC.	Primulaceae	0	0	0	0	0	37	0	0	0	0	0	0	0	0	0	0	0	0	0	0	0	0	0	0	0	0	0	0	0	0	0	0	0	0	0	0	0	0	0	0	0	0	0	0	1	2 %
Euodia hortensis J.R. Forst. & G. Forst.	Rutaceae	0	0	0	0	0	0	0	0	0	0	0	0	0	0	0	0	0	0	0	0	0	0	0	0	0	0	0	0	0	0	0	0	0	1407	0	0	0	0	0	0	0	0	0	0	1	2 %
Evolvulus alsinoides (L.) L.	Convolvulaceae	0	0	0	0	0	0	0	0	0	0	0	0	0	0	0	0	0	0	0	0	0	0	0	0	0	0	0	0	0	0	0	0	0	0	0	0	0	1265	0	0	0	0	0	0	1	2 %
Fagopyrum esculentum Moench	Polygonaceae	0	0	0	0	0	0	0	0	0	0	0	0	0	0	0	0	0	0	0	0	0	0	25	0	0	0	0	0	0	0	0	0	0	0	0	0	0	0	0	0	0	0	0	0	1	2 %
Festuca gigantea (L.) Vill.	Poaceae	0	0	0	0	0	0	0	0	0	0	0	0	0	0	0	0	0	62	0	0	0	0	0	0	0	0	0	0	0	0	0	0	0	0	0	0	0	0	0	0	0	0	0	0	1	2 %
Foeniculum vulgare Mill.	Apiaceae	15	0	57	0	0	2645	0	0	0	221	0	0	0	0	0	0	0	0	0	0	0	0	410	0	0	0	0	0	0	0	0	0	0	0	0	0	0	1286	0	352	0	0	0	0	7	16 %
Fraxinus albicans Buckley	Oleaceae	0	0	0	0	0	0	0	0	0	0	0	0	0	0	0	0	0	0	426	0	0	0	0	0	0	0	0	0	0	0	0	0	0	0	0	0	0	0	0	0	0	0	0	0	1	2 %
Fraxinus pennsylvanica Marshall	Oleaceae	0	0	0	0	0	0	0	0	0	0	0	0	0	0	0	0	412	0	0	0	0	0	0	0	0	0	0	0	0	0	0	0	0	0	0	0	0	0	0	0	0	0	0	0	1	2 %
Glycyrrhiza glabra L.	Leguminosae	0	0	0	0	0	0	0	0	0	0	0	0	0	0	0	0	0	0	0	0	0	0	0	0	204	0	665	0	0	0	0	0	0	0	0	0	0	0	0	0	0	0	0	0	2	5 %
Gossypium hirsutum L.	Malvaceae	0	0	0	0	0	0	0	0	0	0	0	0	0	0	0	0	0	0	0	0	0	0	0	0	0	0	0	0	0	0	0	0	0	0	0	0	0	12	0	401	0	0	0	0	2	5 %
Greeniopsis sibuyanensis Elmer	Rubiaceae	0	0	0	0	0	0	0	0	0	21	0	0	0	0	0	0	0	0	0	0	0	0	0	0	0	0	0	0	0	0	0	0	0	0	0	0	0	0	0	0	0	0	0	0	1	2 %
Gymnema sylvestre (Retz.) R.Br. ex Sm.	Apocynaceae	0	0	0	0	0	0	0	0	0	0	0	0	0	0	0	0	0	0	0	0	0	0	76	0	0	0	0	0	0	0	0	0	0	0	0	0	12	0	0	0	0	0	0	0	2	5 %
Helwingia chinensis Batalin	Helwingiaceae	0	0	0	0	0	0	0	0	0	0	0	0	0	0	0	0	0	0	0	0	0	0	0	0	0	0	0	0	0	0	0	0	0	0	0	0	0	0	0	380	0	0	0	0	1	2 %
Hibiscus sabdariffa L.	Malvaceae	0	0	0	0	0	0	0	0	0	0	0	0	0	0	0	0	0	0	0	0	0	0	0	0	0	0	0	0	0	0	0	0	0	0	0	0	0	488	0	312	0	0	0	0	2	5 %
Hibiscus syriacus L.	Malvaceae	76	0	0	0	0	0	0	0	0	0	0	0	0	0	0	0	0	0	0	0	0	0	0	0	0	0	0	0	0	0	0	0	0	103	0	0	0	0	0	0	0	0	0	0	2	5 %
Hieracium fuscum Vill.	Compositae	0	0	0	0	0	0	0	0	0	0	0	0	0	0	0	0	0	0	0	0	0	0	0	0	0	0	0	0	0	0	0	0	0	0	0	0	0	0	2022	0	0	0	0	0	1	2 %
Hieronyma alchorneoides Allemão	Phyllanthaceae	0	0	0	0	0	0	0	0	0	0	0	0	0	0	0	0	0	0	0	0	0	0	0	0	0	0	0	0	0	0	0	0	0	121	0	0	0	0	0	0	0	0	0	0	1	2 %
Homalium ceylanicum (Gardner) Benth.	Salicaceae	0	0	0	0	0	0	0	0	0	0	0	0	0	0	0	0	0	0	0	0	0	0	0	0	0	0	0	0	0	0	0	0	0	0	0	0	0	0	0	12	0	0	0	0	1	2 %
Hordeum vulgare L.	Poaceae	0	0	0	0	0	0	0	0	0	0	0	0	0	0	0	0	0	0	0	0	0	0	0	0	0	0	0	0	0	0	0	0	0	0	0	0	0	0	0	59	0	0	0	0	1	2 %
Humulus lupulus L.	Cannabaceae	0	0	0	0	0	0	0	0	0	0	0	0	0	0	0	0	0	0	0	0	0	0	0	0	0	0	0	0	0	0	0	0	13440	0	0	0	0	0	0	0	0	0	0	0	1	2 %
Hygrophila auriculata (Schumach.) Heine	Acanthaceae	0	0	0	0	0	0	0	0	0	0	0	0	0	0	0	0	0	0	54	0	0	0	0	0	0	0	0	0	0	0	0	0	0	0	0	0	0	0	0	0	0	0	0	0	1	2 %
Hygrophila schulli M.R.Almeida & S.M.Almeida	Acanthaceae	0	0	82	0	0	0	0	0	0	0	0	0	0	0	0	0	0	0	0	0	0	0	0	0	0	0	0	0	0	0	0	0	0	0	0	0	0	0	0	0	0	0	0	0	1	2 %
Hyoscyamus niger L.	Solanaceae	0	0	0	0	0	0	0	0	0	0	0	0	0	0	0	0	0	0	282	0	0	0	0	0	0	0	0	0	0	0	0	0	0	0	0	0	0	0	0	0	0	0	0	0	1	2 %
Hypochaeris maculata L.	Compositae	0	0	0	0	0	0	0	0	0	0	0	0	0	0	0	0	0	0	0	0	0	0	0	0	0	0	0	0	0	0	0	0	0	0	0	0	0	0	107	107	0	0	0	0	2	5 %
Inula racemosa Hook.f.	Compositae	0	0	0	0	0	0	0	0	0	0	0	0	0	0	0	0	0	0	0	0	0	0	0	0	0	0	290	0	0	0	0	0	0	0	0	0	0	0	0	0	0	0	0	0	1	2 %
Ipomoea nil (L.) Roth	Convolvulaceae	0	39	451	0	0	0	0	0	0	0	0	0	0	0	0	0	0	0	0	0	0	0	0	0	0	0	0	0	0	0	0	0	0	0	0	0	0	0	0	0	0	0	0	0	2	5 %
Ipomoea parasitica (Kunth) G. Don	Convolvulaceae	6088	14746	450	180	0	43	969	0	0	0	0	0	0	0	0	0	0	0	0	0	0	0	17280	0	0	0	0	0	0	0	0	0	0	0	0	0	188	327	0	2442	0	0	0	0	10	23 %
Jatropha curcas L.	Euphorbiaceae	0	0	0	0	0	33	0	0	0	0	0	0	0	0	0	0	0	0	0	0	0	0	0	0	0	0	0	0	0	0	0	0	0	0	0	0	0	30	0	37	0	0	0	0	3	7 %
Justicia adhatoda L.	Acanthaceae	0	0	0	0	0	0	0	0	0	0	0	0	0	0	0	0	0	0	0	0	0	0	0	0	0	0	185	0	0	0	23	0	0	0	0	0	4299	33046	0	0	0	0	0	0	4	9 %
Lactuca sativa L.	Compositae	0	0	0	0	0	0	0	0	0	11	0	62	0	0	0	0	0	0	0	0	0	0	0	0	0	0	0	0	0	0	0	0	0	0	0	0	0	0	3458	3458	0	0	0	0	4	9 %
Lawsonia inermis L.	Lythraceae	0	0	0	0	0	0	0	0	0	0	0	0	0	0	0	0	0	0	0	0	0	0	0	0	0	0	0	0	0	0	0	0	0	0	0	0	0	0	0	0	0	0	72	0	1	2 %
Lophocereus marginatus (DC.) S.Arias & Terrazas	Cactaceae	0	0	0	0	0	0	0	0	0	0	0	0	0	0	0	0	0	0	0	0	0	0	0	0	0	0	0	0	0	0	0	0	0	0	0	0	0	0	0	0	0	0	0	57	1	2 %
Loxocarpus alata R.Br.	Gesneriaceae	0	0	0	0	0	0	0	0	0	0	0	0	0	0	0	0	0	0	0	0	0	0	0	0	0	0	0	0	19	0	0	0	0	0	0	0	0	0	0	0	0	0	0	0	1	2 %
Luffa echinata Roxb.	Cucurbitaceae	0	0	0	0	0	0	0	0	0	0	0	0	0	0	0	132	0	0	0	0	0	0	0	0	0	0	0	0	0	0	0	0	0	0	0	0	0	0	0	0	0	0	0	0	1	2 %
Melissa officinalis L.	Lamiaceae	0	0	0	0	0	0	0	0	0	0	0	0	0	0	0	0	621	0	7481	0	0	0	0	0	0	0	0	0	0	0	10	0	0	0	0	0	0	0	0	0	0	0	0	0	3	7 %
Mentha arvensis L.	Lamiaceae	0	0	0	0	0	0	0	0	0	0	0	0	0	0	0	0	0	0	0	0	0	0	0	0	0	0	0	0	0	0	0	0	0	0	0	0	0	21	0	0	0	0	0	0	1	2 %
Mentha canadensis L.	Lamiaceae	0	0	0	0	0	0	0	0	0	33	0	0	0	0	0	0	0	0	0	0	0	0	0	0	0	0	0	0	0	0	0	0	0	0	0	0	0	0	0	59	0	0	0	0	2	5 %
Mentha spicata L.	Lamiaceae	0	0	0	0	0	0	0	0	0	0	0	0	0	0	0	0	0	0	0	68	0	0	0	0	0	0	104	0	0	0	0	0	0	0	0	0	0	0	0	0	0	0	23	0	3	7 %
Microberlinia brazzavillensis A.Chev.	Leguminosae	0	0	0	0	0	0	0	0	0	0	0	0	0	0	0	0	0	0	0	0	0	0	0	0	0	0	0	0	0	0	0	0	0	0	0	0	0	0	0	14	0	0	0	0	1	2 %
Momordica charantia L.	Cucurbitaceae	0	0	121	0	0	53	0	0	0	0	0	0	0	0	0	0	0	95	0	0	0	0	15	0	0	0	0	0	0	0	202	0	0	343	0	0	0	0	0	0	0	0	0	0	6	14 %
Murraya koenigii (L.) Spreng.	Rutaceae	0	11	0	0	0	0	0	0	0	0	0	0	0	0	0	0	0	0	0	0	0	0	0	0	0	0	0	0	0	0	0	0	0	0	0	0	0	0	0	0	0	0	0	0	1	2 %
Musa acuminata Colla	Musaceae	0	0	0	0	0	0	0	0	0	0	0	0	0	0	0	0	0	0	0	0	0	457	0	0	0	0	0	0	0	0	0	0	0	0	0	0	0	0	0	0	0	0	0	0	1	2 %
Musa laterita Cheesman	Musaceae	0	0	0	0	0	50	0	0	0	0	0	0	0	0	0	0	0	0	0	0	0	0	0	0	0	0	0	0	0	0	0	0	0	0	0	0	0	0	0	0	0	0	0	0	1	2 %
Nardostachys jatamansi (D.Don) DC.	Caprifoliaceae	0	0	0	0	0	0	0	0	0	0	0	0	0	0	0	0	0	0	0	0	0	0	0	0	0	16	0	0	0	0	0	0	0	0	0	0	0	0	0	0	0	0	0	0	1	2 %
Nelumbo nucifera Gaertn.	Nelumbonaceae	0	0	0	0	0	0	0	0	0	0	0	0	0	0	0	517	0	0	0	0	0	0	0	0	0	5058	0	0	0	0	0	0	0	0	0	0	0	0	0	0	0	0	0	0	2	5 %
Nuxia oppositifolia (Hochst.) Benth.	Stilbaceae	0	0	0	0	0	0	0	0	0	0	0	0	0	0	0	0	0	0	0	0	0	0	0	0	0	0	0	0	0	0	0	0	0	0	0	0	0	0	0	520	0	0	0	0	1	2 %
Nyctanthes arbor-tristis L.	Oleaceae	0	0	0	0	0	0	0	0	0	0	0	0	0	0	0	0	0	0	0	0	0	0	0	0	0	0	132	0	0	0	0	0	0	0	0	0	0	0	0	0	0	0	0	0	1	2 %
Ocimum basilicum L.	Lamiaceae	0	0	0	0	0	0	0	0	371	0	0	0	0	0	0	0	0	0	0	0	0	0	0	0	0	0	0	0	2913	0	0	0	0	17	0	1292	477	61	0	40	0	1063	74	317	10	23 %
Ocimum tenuiflorum L.	Lamiaceae	0	0	0	0	0	0	0	0	133	0	0	0	0	0	0	0	0	0	0	0	0	0	0	0	176	0	0	0	592	0	0	0	0	0	34	292	0	328	0	204	0	793	0	0	8	18 %
Oddoniodendron normandii Aubrev.	Leguminosae	0	0	0	0	0	0	0	0	0	0	0	0	0	0	0	0	0	0	0	0	0	0	0	0	0	0	0	0	0	0	0	0	0	0	0	0	137	0	0	0	0	0	0	0	1	2 %
Oenothera drummondii Hook.	Onagraceae	0	0	0	0	0	0	0	0	0	0	0	0	0	0	18	0	0	0	0	0	0	0	0	0	0	0	0	0	0	0	0	0	0	0	0	0	0	0	0	0	0	0	0	0	1	2 %
Oenothera laciniata Hill	Onagraceae	0	0	0	0	0	0	0	0	0	0	0	0	0	0	570	0	0	0	0	0	0	0	0	0	0	0	0	0	0	0	0	0	0	0	0	0	0	0	0	0	0	0	0	0	1	2 %
Olea europaea L.	Oleaceae	0	0	0	0	0	0	0	0	0	0	0	0	0	0	0	0	0	0	0	0	0	0	0	0	0	0	0	455	0	0	0	0	0	0	0	0	0	0	0	0	0	0	0	0	1	2 %
Oryza sativa L.	Poaceae	0	0	0	0	0	0	0	0	0	0	0	0	0	0	0	0	0	0	0	0	0	0	0	0	0	0	0	0	0	0	0	0	0	0	0	0	0	0	0	179	0	0	0	0	1	2 %
Parthenium hysterophorus L.	Compositae	0	0	0	0	0	49	0	0	0	0	0	0	0	0	0	0	0	0	0	0	0	0	0	0	0	0	0	0	0	0	0	0	0	0	0	0	0	0	0	0	0	0	0	0	1	2 %
Pedicularis flammea L.	Orobanchaceae	0	0	0	0	0	0	0	0	0	0	0	0	0	0	0	0	83	0	0	0	0	0	0	0	0	0	0	0	0	0	0	0	0	0	0	0	0	0	0	0	0	0	0	0	1	2 %
Perilla setoyensis G.Honda	Lamiaceae	0	0	0	0	0	0	0	142	0	0	0	0	0	0	0	0	0	0	0	0	0	0	0	0	71	0	0	0	142	0	0	0	0	0	0	23	0	14	0	0	0	87	0	0	6	14 %
Phyllanthus emblica L.	Phyllanthaceae	0	0	0	0	0	0	0	0	0	0	0	0	0	0	0	0	0	0	0	0	0	0	18	0	0	0	0	0	0	0	0	0	0	0	0	0	0	0	0	0	0	0	0	0	1	2 %
Phyllanthus maderaspatensis L.	Phyllanthaceae	0	0	0	0	0	0	0	0	0	0	0	89	0	0	0	0	0	0	0	0	0	0	0	0	0	0	0	0	0	0	0	0	0	0	772	0	0	0	0	0	12	0	0	0	3	7 %

Phyllanthus tenellus Roxb.	Phyllanthaceae	0	0	0	0	0	0	0	0	0	0	0	0	0	0	0	0	0	0	0	0	0	0	0	0	0	0	0	0	0	0	0	0	0	0	2742	0	0	0	0	0	0	0	0	0	1	2 %
Piper longum L.	Piperaceae	0	0	0	0	0	0	0	0	0	0	0	0	0	0	0	126	0	0	0	0	0	0	0	0	3879	5156	4289	3117	0	0	0	0	0	0	0	0	104	0	0	0	0	0	0	0	6	14 %
Piper nigrum L.	Piperaceae	0	0	0	0	0	0	0	0	0	0	0	0	32123	0	0	0	0	0	0	0	0	0	0	0	132	75	1175	14	0	0	0	0	0	0	0	0	0	0	0	0	0	0	0	0	5	11 %
Pistacia atlantica Desf.	Anacardiaceae	0	0	0	0	0	0	0	0	0	0	0	0	0	0	0	0	0	0	0	0	0	0	0	0	0	0	12	0	0	0	0	0	0	0	0	0	0	0	0	0	0	0	0	0	1	2 %
Pisum sativum L.	Leguminosae	0	0	0	0	0	0	0	0	0	0	0	0	0	0	0	0	0	0	0	0	0	0	0	0	0	0	0	0	0	0	0	0	0	0	0	0	0	0	0	0	0	0	0	14	1	2 %
Plantago afra L.	Plantaginaceae	14	0	0	0	0	0	0	0	0	0	0	0	0	0	0	0	0	0	0	0	0	0	0	0	0	0	0	0	0	0	0	0	0	0	0	0	0	0	0	0	0	0	0	0	1	2 %
Plantago major L.	Plantaginaceae	0	0	0	0	0	0	0	0	0	0	0	0	0	0	0	0	0	0	0	0	0	0	0	0	0	0	107	0	0	0	0	0	0	0	0	0	0	0	0	0	0	0	0	0	1	2 %
Plantago ovata Forssk.	Plantaginaceae	0	0	12	27	0	0	0	0	101	0	0	0	0	0	0	0	0	50	0	0	0	0	0	0	0	0	0	0	0	0	0	0	0	0	0	0	0	0	228	0	0	0	0	0	5	11 %
Pleuropetalum darwinii Hook. f.	Amaranthaceae	0	0	0	0	0	0	0	0	0	0	0	0	0	0	0	0	0	0	0	0	0	0	0	0	0	0	0	0	0	0	0	0	0	0	0	0	0	0	0	12	0	0	0	0	1	2 %
Plukenetia madagascariensis Leandri	Euphorbiaceae	0	0	0	0	0	0	0	0	0	0	0	0	0	0	0	0	0	0	0	0	0	0	0	0	0	0	0	0	0	0	0	0	0	11	0	0	0	0	0	0	0	0	0	0	1	2 %
Polypogon monspeliensis (L.) Desf.	Poaceae	0	0	0	0	0	0	0	0	0	0	0	0	0	0	0	0	0	0	0	0	0	0	0	0	0	0	0	0	0	0	132	0	0	0	0	0	0	0	0	0	0	0	0	0	1	2 %
Pseudoselago serrata (P.J. Bergius) Hilliard	Scrophulariaceae	0	0	0	0	0	0	0	0	0	0	0	0	0	0	0	0	0	0	0	0	0	0	0	0	0	0	0	0	0	0	0	0	0	0	0	0	0	0	0	138	0	0	15	0	2	5 %
Punica granatum L.	Lythraceae	0	0	0	0	0	51	0	0	0	1591	0	0	0	0	0	0	0	0	0	0	0	0	0	0	0	0	0	0	0	0	0	0	0	0	0	0	0	0	0	0	0	0	0	0	2	5 %
Pyrus calleryana Decne.	Rosaceae)	0	0	0	0	0	0	0	0	0	0	0	0	0	0	0	0	0	0	0	14	0	0	0	0	0	29	0	0	0	0	0	0	0	0	0	0	0	0	0	0	0	0	0	0	2	5 %
Raphanus sativus L.	Brassicaceae	0	0	52	0	0	0	0	0	0	0	0	0	0	0	0	0	0	0	0	0	0	0	0	0	0	0	0	34	0	0	0	0	0	0	0	0	0	0	0	0	0	0	0	0	2	5 %
Rauvolfia serpentina (L.) Benth. ex Kurz	Apocynaceae	0	0	0	0	0	0	0	0	0	0	0	0	0	0	0	0	0	0	0	0	0	0	0	0	0	0	0	0	0	0	0	0	0	0	0	0	0	0	0	84	0	0	0	0	1	2 %
Robinia pseudoacacia L.	Leguminosae	0	0	0	0	0	0	0	0	0	0	0	0	0	0	0	0	0	0	0	53	0	0	0	0	0	0	0	0	0	0	0	0	0	0	0	0	0	0	0	0	0	0	0	0	1	2 %
Rosa rugosa Thunb.	Rosaceae	0	0	0	18	0	0	0	0	0	0	0	0	0	0	0	0	0	0	0	0	0	0	0	0	0	51	10	0	0	0	0	0	0	0	0	0	0	0	0	0	0	0	0	0	3	7 %
Salvia plebeia R.Br.	Lamiaceae	0	0	0	0	0	0	0	0	0	0	0	0	0	0	0	0	0	0	24	0	0	0	0	0	0	0	0	0	0	0	0	0	0	0	0	0	543	85	0	0	0	0	0	0	3	7 %
Salvia rosmarinus Schleid.	Lamiaceae	0	0	0	0	0	0	0	0	0	0	0	0	0	0	0	0	0	0	0	0	0	0	0	0	0	0	0	0	0	0	0	0	0	143	0	0	0	0	0	0	0	0	0	0	1	2 %
Saraca asoca (Roxb.) Willd.	Leguminosae	0	0	0	0	0	0	0	0	0	0	0	0	0	0	0	0	0	0	0	0	0	0	0	0	0	0	0	0	0	0	0	0	0	0	0	0	15578	785	0	0	0	0	0	0	2	5 %
Secale cereale L.	Poaceae	0	0	0	0	0	0	0	0	0	0	0	0	0	0	0	0	0	0	0	0	0	0	0	0	0	0	0	0	0	0	0	0	0	0	0	0	0	0	0	0	0	0	82	0	1	2 %
Sedum japonicum Siebold ex Miq.	Crassulaceae	0	0	33	0	0	0	0	0	0	0	0	0	0	0	0	0	0	0	0	0	0	0	0	0	0	0	0	0	0	0	0	0	0	0	0	0	0	0	0	0	0	0	0	0	1	2 %
Senna alexandrina Mill.	Leguminosae	4000	0	0	0	0	2968	0	0	0	4887	0	0	0	0	0	157	0	0	85	0	0	0	4484	0	0	422	0	0	0	0	0	0	0	6633	0	0	0	50	0	1570	0	0	0	0	10	23 %
Senna auriculata (L.) Roxb.	Leguminosae	0	0	0	0	0	0	0	0	0	0	0	0	0	0	0	0	0	0	0	0	0	0	0	0	0	559	0	0	0	0	0	0	0	0	0	0	0	0	0	0	0	0	0	0	1	2 %
Senna occidentalis (L.) Link	Leguminosae	0	0	0	0	0	0	18395	0	0	0	0	0	0	0	0	0	0	0	0	0	0	0	0	0	0	0	44	0	0	0	0	0	0	0	0	0	0	0	0	0	0	0	0	0	2	5 %
Senna tora (L.) Roxb.	Leguminosae	0	0	0	0	0	0	0	0	0	696	0	0	0	0	0	0	0	0	0	0	0	0	0	0	0	0	0	0	0	0	0	0	0	0	0	0	0	0	0	162	0	0	291	0	3	7 %
Sesamum indicum L.	Pedaliaceae	0	0	0	0	0	0	0	0	0	0	0	0	0	0	0	0	0	0	0	0	0	0	0	0	0	25	69	0	0	0	0	0	0	0	0	0	0	0	0	92	0	0	0	0	3	7 %
Sida acuta Burm.f.	Malvaceae	0	0	0	0	0	0	0	0	0	0	0	0	0	0	0	0	0	0	0	0	0	0	0	0	37	0	117	0	0	0	5789	0	0	394	0	0	0	0	0	0	0	16	222	0	6	14 %
Sida cordifolia L.	Malvaceae	0	0	0	0	0	0	0	0	0	0	0	0	0	0	0	0	0	0	0	0	0	0	0	0	0	0	0	0	0	0	47	0	0	0	0	0	0	0	0	150	0	0	0	0	2	5 %
Silene uralensis (Rupr.) Bocquet	Caryophyllaceae	0	0	0	0	0	0	0	0	0	0	0	0	0	0	0	0	0	0	0	0	0	0	0	0	0	0	0	0	0	0	0	0	0	0	0	0	0	0	0	13	0	0	0	0	1	2 %
Solanum lycopersicum L.	Solanaceae	0	0	0	0	0	0	0	0	0	30	0	0	189	17	0	0	154	0	0	0	171	0	0	0	0	0	0	0	0	0	0	0	120	0	0	0	32	0	0	0	231	0	0	0	8	18 %
Solanum nigrum L.	Solanaceae	0	0	0	0	0	0	32	0	0	0	0	0	0	0	0	0	0	0	0	0	0	0	0	0	0	0	0	0	0	0	0	0	0	0	0	0	0	0	0	0	0	0	0	0	1	2 %
Solanum virginianum L.	Solanaceae	0	0	0	0	0	0	0	0	0	0	0	0	0	0	0	0	0	0	0	0	0	0	0	0	0	0	0	0	0	0	124	0	0	0	0	0	0	0	0	0	0	0	0	0	1	2 %
Sorghum bicolor (L.) Moench	Poaceae	0	0	0	0	0	0	0	0	0	0	0	0	0	0	0	0	0	0	0	0	0	0	0	0	0	0	0	0	0	0	0	0	0	0	0	0	0	0	0	2342	0	0	0	0	1	2 %
Spondias tuberosa Arruda	Anacardiaceae	0	0	0	0	0	0	0	0	0	0	0	0	0	0	0	0	0	0	0	0	0	0	0	0	0	0	0	0	0	0	0	0	0	0	0	15	0	0	0	0	0	10	0	0	2	5 %
Stenocereus chrysocarpus Sánchez-Mej.	Cactaceae	0	0	0	0	0	0	0	0	0	0	0	0	0	0	0	0	0	0	0	0	0	0	0	0	0	0	0	0	0	0	0	0	0	0	0	0	0	0	0	0	0	0	0	19	1	2 %
Strychnos nux-vomica L.	Loganiaceae	0	0	0	0	0	0	0	0	0	30	0	0	0	0	0	0	0	0	0	0	0	0	0	0	0	0	0	0	0	0	0	0	0	0	0	0	0	0	0	0	0	0	0	0	1	2 %
Swertia chirayita (Roxb.) Buch.-Ham. ex C.B.Clarke	Gentianaceae	0	0	0	0	0	0	0	0	0	0	0	0	0	0	0	0	0	0	0	0	0	0	0	0	0	0	0	0	0	0	0	0	0	0	0	0	0	11	304	0	0	0	0	0	2	5 %
Tamarindus indica L.	Leguminosae	0	0	0	0	0	0	0	0	0	0	0	0	0	0	0	0	0	0	0	0	0	0	0	0	0	0	0	0	0	0	0	0	0	0	0	0	0	0	0	21	0	0	0	0	1	2 %
Taraxacum officinale (L.) Weber ex F.H.Wigg.	Compositae	0	0	0	0	0	0	0	0	0	0	0	0	0	0	0	0	0	0	0	0	0	0	0	0	0	0	0	0	0	0	0	0	0	0	0	0	0	2801	772	0	0	0	0	0	2	5 %
Terminalia arjuna (Roxb. ex DC.) Wight & Arn.	Combretaceae	0	0	0	0	0	0	0	0	0	0	0	0	0	0	0	0	0	0	0	0	0	0	0	0	11	0	478	0	0	0	0	0	0	0	0	0	0	0	0	0	0	0	0	0	2	5 %
Terminalia bellirica (Gaertn.) Roxb.	Combretaceae	0	0	0	0	0	0	0	0	0	0	0	0	0	0	0	0	0	0	0	0	0	0	12	4239	2161	0	0	0	0	0	0	0	0	0	0	0	0	0	0	0	0	0	0	0	3	7 %
Terminalia chebula Retz.	Combretaceae	0	0	0	0	0	0	0	0	0	0	0	0	0	0	0	0	0	0	0	0	0	0	0	0	0	0	0	0	0	0	10	0	0	0	0	0	0	0	0	0	0	0	0	0	1	2 %
Thiloa glaucocarpa (Mart.) Eichler	Combretaceae	0	0	0	0	0	0	0	0	0	0	0	0	0	0	0	0	0	0	0	0	0	0	0	0	0	0	0	0	0	0	0	0	0	0	0	0	0	0	0	177	0	0	0	0	1	2 %
Thysanolaena latifolia (Roxb. ex Hornem.) Honda	Poaceae	0	0	0	0	0	0	0	0	0	0	0	0	0	0	0	0	0	8805	0	0	0	0	0	0	0	0	0	0	0	0	0	0	0	0	0	0	0	0	0	0	0	0	0	0	1	2 %
Tinospora cordifolia (Willd.) Miers	Menispermaceae	0	0	0	0	0	0	0	0	0	0	0	0	0	0	0	0	0	0	0	0	0	0	0	0	0	0	0	0	0	0	0	672	0	0	298	134	0	10	0	0	0	0	0	0	4	9 %
Trachyspermum ammi (L.) Sprague	Apiaceae	0	0	0	0	0	175	12	0	0	62	0	0	0	0	0	0	0	0	0	0	0	0	10	0	0	0	0	13758	0	0	0	0	0	0	0	0	0	0	0	0	0	0	0	0	5	11 %
Trianthema portulacastrum L.	Aizoaceae	0	0	0	0	0	0	0	0	0	0	0	0	0	0	0	418	0	0	0	68	0	0	0	0	0	0	0	0	0	0	422	0	0	27175	0	0	353	486	1135	0	0	0	0	0	7	16 %
Tribulus terrestris L.	Zygophyllaceae	0	0	0	0	0	188	0	0	0	164	16	0	0	0	24385	27	0	0	0	0	0	0	0	0	15	2599	3006	28	0	0	7870	0	0	0	0	0	57	0	0	0	0	0	0	31	12	27 %
Trigastrotheca molluginea F.Muell.	Molluginaceae	0	0	0	0	0	0	0	0	0	0	0	0	0	0	0	0	0	0	0	0	0	0	0	0	0	0	0	0	0	0	0	0	0	0	39	0	0	0	0	0	0	0	0	0	1	2 %
Trigonella foenum-graecum L.	Leguminosae	0	1108	0	0	0	12	0	0	0	0	0	0	0	0	0	498	0	0	0	0	0	0	0	0	0	0	0	4733	0	0	0	0	0	0	0	0	0	0	0	0	0	0	0	0	4	9 %
Triticum aestivum L.	Poaceae	0	0	0	0	0	10	0	0	0	0	25	0	0	18	0	0	0	0	0	0	0	0	0	0	0	0	0	0	0	0	0	0	0	0	0	0	0	15	0	0	0	0	139	0	5	11 %
Veronica chamaedrys L.	Plantaginaceae	0	0	0	0	0	0	0	0	0	0	0	0	1501	0	0	0	0	0	0	0	0	889	0	0	0	0	0	0	0	0	0	0	0	0	0	0	0	0	0	0	0	0	0	0	2	5 %
Veronica plebeia R. Br.	Plantaginaceae	0	0	0	0	0	0	0	0	0	0	0	0	0	0	0	0	0	0	0	0	0	0	0	0	0	120	0	0	0	0	0	0	0	0	0	0	0	0	0	0	0	0	0	0	1	2 %
Vigna mungo (L.) Hepper	Leguminosae	0	0	0	0	0	0	0	0	0	0	0	0	0	0	0	0	0	0	0	0	0	0	0	0	0	0	0	0	0	0	28	0	0	0	0	0	0	0	0	0	0	12	0	0	2	5 %
Vigna radiata (L.) R.Wilczek	Leguminosae	0	0	0	0	0	0	0	0	0	0	0	0	0	0	0	0	0	0	0	0	0	0	0	0	0	0	696	0	0	0	0	33	0	0	0	0	0	0	0	0	0	0	0	0	2	5 %
Vigna unguiculata (L.) Walp.	Leguminosae	0	0	0	0	0	0	0	0	0	0	0	0	0	0	0	0	0	0	0	0	0	0	0	0	0	0	0	0	0	0	0	0	0	0	0	0	0	0	0	13	0	0	0	0	1	2 %
Vitex agnus-castus L.	Lamiaceae	0	0	0	0	0	0	0	0	0	0	0	0	0	0	0	0	0	0	0	0	0	0	0	0	0	0	0	0	14	0	0	0	0	0	0	0	0	0	0	0	0	0	0	0	1	2 %
Withania somnifera (L.) Dunal	Solanaceae	177	0	2318	145	42	647	325	0	0	3354	0	0	0	0	0	0	574	0	31	0	0	0	147	0	0	525	593	0	0	0	0	64	0	0	0	0	74	0	0	693	127	0	17	0	17	39 %
Xenostegia tridentata (L.) D.F. Austin & Staples	Convolvulaceae	0	0	0	0	0	0	0	0	0	0	0	0	0	0	0	0	0	0	0	0	0	0	0	0	0	0	0	0	0	0	0	0	0	0	0	0	0	0	0	55	0	0	0	0	1	2 %
Xeronema callistemon W.R.B.Oliv.	Xeronemataceae	0	0	0	0	0	0	0	0	0	0	0	0	0	0	0	0	0	0	0	0	0	0	0	0	0	0	0	0	0	0	0	0	0	0	0	0	0	0	0	0	0	0	0	284	1	2 %
Zea mays L.	Poaceae	0	0	10	0	0	56	0	0	0	0	0	0	0	0	0	0	0	0	0	0	0	0	0	0	0	0	0	0	0	0	0	0	0	0	0	0	0	0	0	48	0	0	0	0	3	7 %
Zingiber officinale Roscoe	Zingiberaceae	0	0	0	0	0	0	0	0	0	0	29	0	0	0	0	0	0	0	0	0	0	0	0	0	0	0	72	42	0	0	0	0	0	0	0	0	0	0	0	0	0	0	0	0	3	7 %
**Total number of reads**		**11188**	**15904**	**4280**	**370**	**42**	**8066**	**20307**	**142**	**44981**	**37148**	**70**	**3420**	**55595**	**108**	**28290**	**10498**	**9941**	**9264**	**8588**	**263**	**207**	**3124**	**23185**	**19144**	**6704**	**16890**	**16647**	**22181**	**5238**	**86865**	**14657**	**1400**	**13637**	**82673**	**5336**	**1876**	**22854**	**47077**	**118329**	**91413**	**43964**	**2136**	**7408**	**1563**		
**Total number of species within the product**		**11**	**4**	**14**	**4**	**1**	**20**	**9**	**1**	**8**	**16**	**3**	**6**	**5**	**4**	**6**	**11**	**9**	**6**	**11**	**7**	**2**	**4**	**21**	**3**	**10**	**20**	**25**	**8**	**9**	**2**	**11**	**5**	**3**	**18**	**8**	**6**	**17**	**25**	**11**	**42**	**9**	**7**	**12**	**8**	**188**	
