# Supplementary figures and images for: DNA Metabarcoding Authentication of Ayurvedic Herbal Products on the European Market Raises Concerns of Quality and Fidelity
Source: Front Plant Sci. 2019 Feb 5;10:68. doi: 10.3389/fpls.2019.00068 (PMC6370972; doi:10.3389/fpls.2019.00068)

| Herbal product ID                                                                                                                                                                                                                                                                                                                                                                                                                                                                                                                                                                                                                                                                                 | 1 | 2 | 3 | 4 |
|---------------------------------------------------------------------------------------------------------------------------------------------------------------------------------------------------------------------------------------------------------------------------------------------------------------------------------------------------------------------------------------------------------------------------------------------------------------------------------------------------------------------------------------------------------------------------------------------------------------------------------------------------------------------------------------------------|---|---|---|---|
| <p> 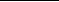 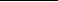 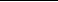 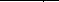 </p> <p> 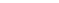 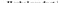 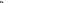 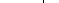 </p> |   |   |   |   |

[illegible]

Supplement: Supplementary file 4 [file Data_Sheet_4.PDF]
