# Supplementary material for: DNA Metabarcoding Authentication of Ayurvedic Herbal Products on the European Market Raises Concerns of Quality and Fidelity
Source: Front Plant Sci. 2019 Feb 5;10:68. doi: 10.3389/fpls.2019.00068 (PMC6370972; doi:10.3389/fpls.2019.00068)
Supplement: Supplementary file 5 [file Data_Sheet_5.PDF]

Supplementary Table S5. Details of sum occurrences of a species in Ayurvedic herbal products used to generate heatmap.

List of plant species	Expected-detected	Expected-Not detected	Not expected-detected	Tablet 1	Tablet 2	Tablet 3	Capsule 5	Tablet 6	Capsule 7	Capsule 8	Capsule 9	Capsule 10	Tablet 12	Tablet 13	Tablet 14	Tablet 16	Tablet 17	Tablet 18	Tablet 19	Tablet 23	Capsule 27	Capsule 30	Tablet 31	Tablet 32	Tablet 34	Tablet 36	Capsule 40	Powder 52	Powder 55	Powder 58	Capsule 60	Capsule 61	Capsule 63	Capsule 66	Capsule 68	Capsule 69	Capsule 70	Tablet 73	Tablet 74	Tablet 75	Powder 77	Powder 79
Abelmoschus esculentus (L.) Moench	0	0	1	0	0	0	0	0	0	0	0	0	0	0	0	0	0	0	0	0	0	0	0	0	0	0	0	0	0	0	0	0	0	0	0	0	0	3	0	0	0	0
Abutilon indicum (L.) Sweet	0	1	0	0	0	0	0	0	0	0	0	0	0	0	2	0	0	0	0	0	0	0	0	0	0	0	0	0	0	0	0	0	0	0	0	0	0	0	0	0	0	0
Acacia arabica (Lam.) Willd.	0	3	0	2	2	2	0	0	0	0	0	0	0	0	0	0	0	0	0	0	0	0	0	0	0	0	0	0	0	0	0	0	0	0	0	0	0	0	0	0	0	0
Achillea millefolium L.	0	1	0	0	0	0	0	0	0	0	0	0	0	2	0	0	0	0	0	0	0	0	0	0	0	0	0	0	0	0	0	0	0	0	0	0	0	0	0	0	0	0
Achyranthes aspera L.	0	2	0	2	0	0	0	0	0	0	0	0	0	0	0	2	0	0	0	0	0	0	0	0	0	0	0	0	0	0	0	0	0	0	0	0	0	0	0	0	0	0
Acorus calamus L.	0	2	0	0	0	0	0	0	0	0	0	0	2	0	0	0	0	0	0	0	0	0	0	0	0	0	0	0	0	0	0	0	0	0	0	0	0	2	0	0	0	0
Aegle marmelos (L.) Corrêa	0	1	0	0	0	0	0	0	0	0	0	0	0	0	0	0	0	0	0	0	0	0	0	0	0	0	0	0	0	0	0	0	0	0	0	0	2	0	0	0	0	0
Albizia lebbeck (L.) Benth.	0	1	0	0	0	0	0	0	0	2	0	0	0	0	0	0	0	0	0	0	0	0	0	0	0	0	0	0	0	0	0	0	0	0	0	0	0	0	0	0	0	0
Allium sativum L.	0	1	0	0	0	0	0	0	2	0	0	0	0	0	0	0	0	0	0	0	0	0	0	0	0	0	0	0	0	0	0	0	0	0	0	0	0	0	0	0	0	0
Aloe vera (L.) Burm.f.	0	2	0	0	0	0	0	2	0	0	0	0	0	0	2	0	0	0	0	0	0	0	0	0	0	0	0	0	0	0	0	0	0	0	0	0	0	0	0	0	0	0
Alpinia galanga (L.) Willd.	0	1	0	0	0	0	0	0	0	0	0	0	0	0	0	0	0	2	0	0	0	0	0	0	0	0	0	0	0	0	0	0	0	0	0	0	0	0	0	0	0	0
Althaea officinalis L.	0	0	3	0	0	0	0	0	0	0	0	0	0	0	0	0	0	3	0	0	0	0	0	0	0	0	0	0	0	0	0	0	0	0	3	3	0	0	0	0	0	0
Amomum subulatum Roxb.	0	1	0	0	0	0	0	0	0	0	0	0	0	0	0	0	0	0	0	0	0	0	0	0	2	0	0	0	0	0	0	0	0	0	0	0	0	0	0	0	0	0
Anchusa italica Retz.	0	0	1	0	0	0	0	0	0	0	0	3	0	0	0	0	0	0	0	0	0	0	0	0	0	0	0	0	0	0	0	0	0	0	0	0	0	0	0	0	0	0
Andrographis paniculata (Burm.f.) Nees	0	1	0	0	0	0	0	0	0	0	0	0	0	0	0	0	0	0	0	0	0	0	0	0	0	2	0	0	0	0	0	0	0	0	0	0	0	0	0	0	0	0
Anethum sowa Roxb. ex Fleming	0	1	0	0	0	0	0	0	0	0	0	0	2	0	0	0	0	0	0	0	0	0	0	0	0	0	0	0	0	0	0	0	0	0	0	0	0	0	0	0	0	0
Asparagus racemosus Willd.	3	5	0	0	0	2	0	0	1	2	0	2	0	2	0	0	0	0	0	2	0	0	0	1	0	0	0	0	0	0	0	0	0	0	0	0	0	0	0	0	1	0
Azadirachta indica A.Juss.	2	1	0	0	2	0	0	0	0	0	0	0	0	0	0	0	0	0	0	0	0	0	0	0	0	0	0	0	0	0	0	0	0	1	0	0	0	0	0	1	0	0
Baccharoides anthelmintica (L.) Moench	0	0	3	0	0	0	0	0	0	0	0	0	0	0	0	0	0	0	0	0	0	0	0	0	0	0	0	0	0	0	0	0	0	0	3	3	3	0	0	0	0	0
Bacopa monnieri (L.) Wettst.	0	3	0	0	0	0	0	0	0	0	0	2	2	0	0	0	0	0	0	0	0	0	0	0	0	0	0	0	0	0	0	0	0	0	0	0	0	0	2	0	0	0
Behnia reticulata (Thunb.) Didr.	0	0	1	0	0	0	0	0	0	0	0	0	0	0	0	0	0	0	0	0	0	0	0	0	0	0	0	0	0	0	0	0	0	0	0	0	0	0	0	0	3	0
Berberis aristata DC.	0	1	0	0	0	0	0	0	0	0	0	0	0	0	2	0	0	0	0	0	0	0	0	0	0	0	0	0	0	0	0	0	0	0	0	0	0	0	0	0	0	0
Bergenia ligulata Engl.	0	1	1	2	0	0	0	0	0	0	0	0	0	0	0	3	0	0	0	0	0	0	0	0	0	0	0	0	0	0	0	0	0	0	0	0	0	0	0	0	0	0
Boerhaavia diffusa L.	1	5	0	2	0	0	0	2	0	0	0	2	0	2	2	0	0	0	0	0	0	0	0	0	0	0	0	0	0	0	0	1	0	0	0	0	0	0	0	0	0	0
Boswellia serrata Roxb. ex Colebr.	0	2	0	0	0	0	0	0	0	0	0	0	0	0	0	0	0	2	0	0	0	0	0	0	0	2	0	0	0	0	0	0	0	0	0	0	0	0	0	0	0	0
Brassica oleracea L.	0	0	2	0	0	0	0	0	0	0	0	0	0	0	3	0	0	0	0	0	3	0	0	0	0	0	0	0	0	0	0	0	0	0	0	0	0	0	0	0	0	0
Caesalpinia bonduc (L.) Roxb.	0	1	0	0	0	0	0	0	0	0	0	0	0	0	0	0	0	0	2	0	0	0	0	0	0	0	0	0	0	0	0	0	0	0	0	0	0	0	0	0	0	0
Calluna vulgaris (L.) Hull	0	0	1	0	0	0	0	0	3	0	0	0	0	0	0	0	0	0	0	0	0	0	0	0	0	0	0	0	0	0	0	0	0	0	0	0	0	0	0	0	0	0
Camellia sinensis (L.) Kuntze	0	1	0	0	0	0	0	0	0	0	0	2	0	0	0	0	0	0	0	0	0	0	0	0	0	0	0	0	0	0	0	0	0	0	0	0	0	0	0	0	0	0
Cannabis sativa L.	0	0	1	0	0	0	0	0	0	3	0	0	0	0	0	0	0	0	0	0	0	0	0	0	0	0	0	0	0	0	0	0	0	0	0	0	0	0	0	0	0	0
Capparis spinosa L.	0	2	0	0	0	0	0	2	0	0	0	0	0	2	0	0	0	0	0	0	0	0	0	0	0	0	0	0	0	0	0	0	0	0	0	0	0	0	0	0	0	0
Casearia esculenta Roxb.	0	1	0	0	0	0	0	0	0	0	0	0	0	0	2	0	0	0	0	0	0	0	0	0	0	0	0	0	0	0	0	0	0	0	0	0	0	0	0	0	0	0
Cassia fistula L.	0	1	0	0	0	0	0	0	0	0	0	0	0	0	0	0	0	0	0	0	0	0	0	0	0	0	0	0	0	0	0	0	0	0	0	0	0	2	0	0	0	0
Celastrus paniculatus Willd.	1	1	0	0	0	0	0	0	0	0	0	0	2	0	0	0	0	0	0	0	0	0	0	0	0	0	0	0	0	0	0	0	0	0	0	0	0	0	1	0	0	0
Cenchrus americanus (L.) Morrone	0	0	1	0	0	0	0	0	0	0	0	0	0	0	0	0	0	0	0	0	0	0	0	0	0	0	0	0	0	0	0	0	0	0	0	0	0	3	0	0	0	0
Centaurea cyanus L.	0	0	1	0	0	0	0	0	0	0	0	0	0	0	0	0	0	0	0	3	0	0	0	0	0	0	0	0	0	0	0	0	0	0	0	0	0	0	0	0	0	0
Centella asiatica (L.) Urb.	1	1	2	0	0	0	0	0	0	0	0	0	2	0	0	0	1	0	0	0	0	0	0	3	0	0	0	0	0	0	0	0	0	0	0	0	0	0	3	0	0	0
Chaerophyllum reflexum Aitch.	0	0	1	0	0	0	0	0	0	0	0	0	0	0	0	0	0	0	0	0	3	0	0	0	0	0	0	0	0	0	0	0	0	0	0	0	0	0	0	0	0	0
Chamaecrista olesiphylla (Vogel) H.S.Irwin & Barneby	0	0	1	3	0	0	0	0	0	0	0	0	0	0	0	0	0	0	0	0	0	0	0	0	0	0	0	0	0	0	0	0	0	0	0	0	0	0	0	0	0	0
Chlorophytum arundinaceum Baker	0	1	0	0	0	2	0	0	0	0	0	0	0	0	0	0	0	0	0	0	0	0	0	0	0	0	0	0	0	0	0	0	0	0	0	0	0	0	0	0	0	0
Cichorium intybus L.	2	0	2	0	0	3	0	1	0	0	3	0	0	1	0	0	0	0	0	0	0	0	0	0	0	0	0	0	0	0	0	0	0	0	0	0	0	0	0	0	0	0
Cinnamomum cassia (L.) J.Presl	0	0	1	0	0	0	0	0	0	0	0	0	0	0	0	0	0	0	0	0	0	0	0	0	3	0	0	0	0	0	0	0	0	0	0	0	0	0	0	0	0	0
Cinnamomum verum J.Presl	0	2	0	0	0	0	0	0	0	0	0	0	0	0	0	0	0	0	0	0	0	0	2	0	2	0	0	0	0	0	0	0	0	0	0	0	0	0	0	0	0	0
Cissus quadrangularis L.	1	0	0	0	0	0	0	0	0	0	0	0	0	0	0	0	0	0	0	0	0	0	0	0	0	0	1	0	0	0	0	0	0	0	0	0	0	0	0	0	0	0
Coccinia grandis (L.) Voigt	0	0	2	0	0	0	0	0	0	0	0	0	0	0	0	0	0	0	0	0	0	0	0	0	0	0	0	0	0	3	0	0	3	0	0	0	0	0	0	0	0	0
Coccinia sessilifolia (Sond.) Cogn.	0	0	1	0	0	0	0	0	0	0	0	0	0	0	0	0	0	0	0	0	3	0	0	0	0	0	0	0	0	0	0	0	0	0	0	0	0	0	0	0	0	0
Commiphora wightii (Arn.) Bhandari	0	1	0	0	0	0	0	0	0	0	0	0	0	0	0	0	0	2	0	0	0	0	0	0	0	0	0	0	0	0	0	0	0	0	0	0	0	0	0	0	0	0
Convolvulus microphyllus Sieber ex Spreng.	0	0	1	0	0	0	0	0	0	0	0	0	0	0	0	0	0	0	0	0	0	0	0	0	3	0	0	0	0	0	0	0	0	0	0	0	0	0	0	0	0	0
Convolvulus prostratus Forssk.	1	0	3	3	0	0	0	0	0	0	0	0	0	0	0	0	0	0	0	0	0	0	0	0	3	0	0	0	0	0	0	0	0	0	0	0	0	3	1	0	0	0
Corchorus olitorius L.	0	0	1	0	0	0	0	0	0	0	0	0	0	0	0	0	0	0	0	0	0	0	0	3	0	0	0	0	0	0	0	0	0	0	0	0	0	0	0	0	0	0
Coriandrum sativum L.	0	0	3	0	0	0	0	3	0	0	0	0	0	0	0	0	0	0	0	0	0	0	0	3	0	0	0	0	0	0	0	0	0	0	0	3	0	0	0	0	0	0
Crateva nurvala Buch.-Ham	0	2	0	2	0	0	0	0	0	0	0	0	0	0	0	0	0	0	2	0	0	0	0	0	0	0	0	0	0	0	0	0	0	0	0	0	0	0	0	0	0	0
Crocus sativus L.	0	1	0	0	0	0	0	0	0	0	0	0	0	0	0	0	0	0	0	2	0	0	0	0	0	0	0	0	0	0	0	0	0	0	0	0	0	0	0	0	0	0
Cullen corylifolium (L.) Medik.	0	0	1	0	0	3	0	0	0	0	0	0	0	0	0	0	0	0	0	0	0	0	0	0	0	0	0	0	0	0	0	0	0	0	0	0	0	0	0	0	0	0
Cuminum cyminum L.	0	0	6	3	0	0	0	3	0	0	0	0	0	0	0	0	0	0	0	0	3	0	0	3	3	0	0	0	0	0	0	3	0	0	0	0	0	0	0	0	0	0
Curcuma longa L.	0	7	0	0	2	0	0	0	0	2	0	0	0	0	2	0	0	0	0	0	0	0	2	2	0	2	0	0	0	0	0	0	0	2	0	0	0	0	0	0	0	0
Cyperus scariosus R.Br.	0	1	0	0	0	0	0	0	0	0	0	0	0	0	0	2	0	0	0	0	0	0	0	0	0	0	0	0	0	0	0	0	0	0	0	0	0	0	0	0	0	0
Damrongia cyanantha Triboun	0	0	1	0	0	0	0	0	0	0	0	0	0	0	0	0	0	0	0	0	0	0	0	0	0	0	3	0	0	0	0	0	0	0	0	0	0	0	0	0	0	0
Didymocarpus pedicellata (Ait.) Ait. F.	0	1	0	0	0	0	0	0	0	0	0	0	0	0	0	2	0	0	0	0	0	0	0	0	0	0	0	0	0	0	0	0	0	0	0	0	0	0	0	0	0	0
Doniophyton weddellii Katinas & Stuessy	0	0	1	0	0	0	0	0	0	0	0	0	0	0	0	0	0	0	0	0	0	0	0	0	0	0	0	0	0	0	0	3	0	0	0	0	0	0	0	0	0	0
Draba nivalis Lilj.	0	0	1	0	0	0	0	0	0	0	0	0	0	0	0	0	0	3	0	0	0	0	0	0	0	0	0	0	0	0	0	0	0	0	0	0	0	0	0	0	0	0
Drymocallis rupestris (L.) Soj k	0	0	1	0	0	0	0	0	0	0	0	0	0	0	0	0	3	0	0	0	0	0	0	0	0	0	0	0	0	0	0	0	0	0	0	0	0	0	0	0	0	0
Drypetes hoaensis Gagnep.	0	0	1	0	0	0	0	0	0	0	0	0	0	0	0	0	0	0	0	0	0	0	0	0	0	0	3	0	0	0	0	0	0	0	0	0	0	0	0	0	0	0
Dyschoriste siphonantha (Nees) Kuntze	0	0	1	0	0	0	0	0	0	0	0	0	0	0	0	0	0	0	0	0	0	0	0	0	0	0	0	0	0	0	0	0	0	0	3	0	0	0	0	0	0	0
Echinochloa colona (L.) Link	0	0	1	0	0	0	0	0	0	0	0	0	0	0	0	0	0	0	0	0	0	0	0	0	0	0	0	0	0	0	0	0	3	0	0	0	0	0	0	0	0	0
Echinops echinatus Roxb.	0	1	0	0	0	0	0	0	0	0	0	0	0	0	0	0	0	0	0	0	0	0	0	0	0	0	0	0	0	0	0	0	0	0	0	0	0	2	0	0	0	0
Eclipta prostrata (L.) L.	1	2	1	0	0	0	0	2	0	0	0	2	0	0	0	0	0	0	0	0	0	0	0	0	0	0	0	1	0	0	0	3	0	0	0	0	0	0	0	0	0	0
Elettaria cardamomum (L.) Maton	0	1	0	0	0	0	0	0	0	0	0	0	2	0	0	0	0	0	0	0	0	0	0	0	0	0	0	0	0	0	0	0	0	0	0	0	0	0	0	0	0	0
Embelia ribes Burm.f.	0	2	0	0	0	0	0	0	0	0	0	0	2	2	0	0	0	0	0	0	0	0	0	0	0	0	0	0	0	0	0	0	0	0	0	0	0	0	0	0	0	0
Enicostemma littorale Blume	0	1	0	0	2	0	0	0	0	0	0	0	0	0	0	0	0	0	0	0	0	0	0	0	0	0	0	0	0	0	0	0	0	0	0	0	0	0	0	0	0	0
Evolvulus alsinoides (L.) L.	0	1	1	0	0	0	0	0	0	0	0	0	2	0	0	0	0	0	0	0	0	0	0	0	0	0	0	0	0	0	0	0	0	0	0	3	0	0	0	0	0	0
Foeniculum vulgare Mill.	0	1	4	0	0	3	0	3	0	0	0	0	0	0	0	0	0	0	0	0	3	0	0	0	0	0	0	0	0	0	0	0	0	0	0	3	0	0	2	0	0	0
Fraxinus albicans Buckley	0	0	1	0	0	0	0	0	0	0	0	0	0	0	0	0	0	0	0	3	0	0	0	0	0	0	0	0	0	0	0	0	0	0	0	0	0	0	0	0	0	0
Fumaria officinalis L.	0	1	0	0	0	0	0	0	0	0	0	0	0	2	0	0	0	0	0	0	0	0	0	0	0	0	0	0	0	0	0	0	0	0	0	0	0	0	0	0	0	0
Glycyrrhiza glabra L.	2	4	0	0	0	0	0	0	0	0	0	0	0	0	2	0	2	2	0	0	0	0	1	0	1	0	0	0	0	0	0	0	0	0	2	0	0	0	0	0	0	0
Gmelina arborea Roxb.	0	1	0	0	0	0	0	0	0	0	0	0	0	0	2	0	0	0	0	0	0	0	0	0	0	0	0	0	0	0	0	0	0	0	0	0	0	0	0	0	0	0
Gossypium herbaceum L.	0	1	0	0	0	0	0	0	0	0	0	0	0	0	2	0	0	0	0	0	0	0	0	0	0	0	0	0	0	0	0	0	0	0	0	0	0	0	0	0	0	0
Gossypium hirsutum L.	0	0	1	0	0	0	0	0	0	0	0	0	0	0	0	0	0	0	0	0	0	0	0	0	0	0	0	0	0	0	0	0	0	0	0	0	0	3	0	0	0	0
Gymnema sylvestre (Retz.) R.Br. ex Sm.	1	2	0	0	2	0	0	0	0	0	0	0	0	0	2	0	0	0	0	0	1	0	0	0	0	0	0	0	0	0	0	0	0	0	0	0	0	0	0	0	0	0
Hemidesmus indicus (L.) R. Br. ex Schult.	0	1	0	0	0	0	0	0	0	0	0	0	0	0	0	0	0	0	0	0	0	0	0	2	0	0	0	0	0	0	0	0	0	0	0	0	0	0	0	0	0	0
Hibiscus sabdariffa L.	0	0	2	0	0	0	0	0	0	0	0	0	0	0	0	0	0	0	0	0	0	0	0	0	0	0	0	0	0	0	0	0	0	0	0	3	0	0	3	0	0	0
Hibiscus syriacus L.	0	0	1	3	0	0	0	0	0	0	0	0	0	0	0	0	0	0	0	0	0	0	0	0	0	0	0	0	0	0	0	0	0	0	0	0	0	0	0	0	0	0
Hordeum vulgare L.	1	0	0	0	0	0	0	0	0	0	0	0	0	0	0	0	0	0	0	0	0	0	0	0	0	0	0	0	0	0	0	0	0	0	0	0	0	1	0	0	0	0
Humulus lupulus L.	0	1	0	0	0	0	0	0	0	0	2	0	0	0	0	0	0	0	0	0	0	0	0	0	0	0	0	0	0	0	0	0	0	0	0	0	0	0	0	0	0	0
Hygrophila auriculata (Schumach.) Heine	1	0	0	0	0	0	0	0	0	0	0	0	0	0	0	0	0	0	0	1	0	0	0	0	0	0	0	0	0	0	0	0	0	0	0	0	0	0	0	0	0	0
Hyoscyamus niger L.	0	0	1	0	0	0	0	0	0	0	0	0	0	0	0	0	0	0	0	3	0	0	0	0	0	0	0	0	0	0	0	0	0	0	0	0	0	0	0	0	0	0
Inula racemosa Hook.f.	0	0	1	0	0	0	0	0	0	0	0	0	0	0	0	0	0	0	0	0	0	0	0	0	3	0	0	0	0	0	0	0	0	0	0	0	0	0	0	0	0	0
Ipomoea digitata L.	0	1	0	0	0	0	0	0	0	0	0	0	2	0	0	0	0	0	0	0	0	0	0	0	0	0	0	0	0	0	0	0	0	0	0	0	0	0	0	0	0	0
Ipomoea parasitica (Kunth) G. Don	0	0	7	3	3	3	0	0	3	0	0	0	0	0	0	0	0	0	0	0	3	0	0	0	0	0	0	0	0	0	0	0	0	0	0	3	0	3	0	0	0	0
Iris germanica L.	0	1	0	0	0	0	0	0	0	0	0	0	0	0	0	0	0	0	0	0	0	0	0	0	2	0	0	0	0	0	0	0	0	0	0	0	0	0	0	0	0	0
Justicia adhatoda L.	0	0	2	0	0	0	0	0	0	0	0	0	0	0	0	0	0	0	0	0	0	0	0	0	0	0	0	0	0	0	0	0	0	0	3	3	0	0	0	0	0	0
Lavandula stoechas L.	0	1	0	0	0	0	0	0	0	0	0	0	0	0	0	0	0	0	0	0	0	0	0	0	0	0	0	0	0	0	0	0	0	0	0	0	0	0	2	0	0	0
Lawsonia inermis L.	0	0	1	0	0	0	0	0	0	0	0	0	0	0	0	0	0	0	0	0	0	0	0	0	0	0	0	0	0	0	0	0	0	0	0	0	0	0	0	0	3	0
Leptadenia reticulata (Retz.) Wight & Arn.	0	1	0	0	0	0	0	0	2	0	0	0	0	0	0	0	0	0	0	0	0	0	0	0	0	0	0	0	0	0	0	0	0	0	0	0	0	0	0	0	0	0
Lotus arabicus L.	0	1	0	0	0	0	0	0	0	0	0	0	0	0	0	0	0	0	0	0	0	0	0	0	0	2	0	0	0	0	0	0	0	0	0	0	0	0	0	0	0	0
Melissa officinalis L.	0	0	1	0	0	0	0	0	0	0	0	0	0	0	0	0	0	0	0	3	0	0	0	0	0	0	0	0	0	0	0	0	0	0	0	0	0	0	0	0	0	0
Momordica charantia L.	0	2	1	0	2	3	0	0	0	0	0	0	0	0	2	0	0	0	0	0	0	0	0	0	0	0	0	0	0	0	0	0	0	0	0	0	0	0	0	0	0	0
Mucuna pruriens (L.) DC.	0	1	0	0	0	0	0	0	0	0	0	0	2	0	0	0	0	0	0	0	0	0	0	0	0	0	0	0	0	0	0	0	0	0	0	0	0	0	0	0	0	0
Myristica fragrans Houtt.	0	1	0	0	0	0	0	0	0	0	0	0	0	0	0	0	0	0	0	0	0	0	0	0	0	2	0	0	0	0	0	0	0	0	0	0	0	0	0	0	0	0
Nardostachys jatamansi (D.Don) DC.	0	2	0	0	0	0	0	0	0	0	0	0	2	0	0	0	0	0	0	0	0	0	0	0	0	0	0	0	0	0	0	0	0	0	0	0	0	0	2	0	0	0
Nelumbo nucifera Gaertn.	0	0	1	0	0	0	0	0	0	0	0	0	0	0	0	0	0	0	0	0	0	0	0	3	0	0	0	0	0	0	0	0	0	0	0	0	0	0	0	0	0	0
Nyctanthes arbor-tristis L.	0	0	1	0	0	0	0	0	0	0	0	0	0	0	0	0	0	0	0	0	0	0	0	0	3	0	0	0	0	0	0	0	0	0	0	0	0	0	0	0	0	0
Ocimum basilicum L.	0	0	5	0	0	0	0	0	0	0	3	0	0	0	0	0	0	0	0	0	0	0	0	0	0	0	3	0	0	0	0	0	0	3	3	0	0	0	0	0	0	3
Ocimum gratissimum L.	0	1	0	0	0	0	0	0	0	0	0	0	0	0	0	0	0	0	0	0	0	0	0	0	0	0	2	0	0	0	0	0	0	0	0	0	0	0	0	0	0	0
Ocimum tenuiflorum L.	3	1	3	0	0	0	0	0	0	0	0	0	0	0	2	0	0	0	0	0	0	0	1	0	0	0	3	0	0	0	0	0	0	1	0	3	0	3	0	1	0	0
Olea europaea L.	0	0	1	0	0	0	0	0	0	0	0	0	0	0	0	0	0	0	0	0	0	0	0	0	0	3	0	0	0	0	0	0	0	0	0	0	0	0	0	0	0	0
Onosma bracteatum Wall	0	2	0	0	0	0	0	0	0	0	0	0	0	0	0	2	0	0	0	0	0	0	0	0	0	0	0	0	0	0	0	0	0	0	0	0	0	0	2	0	0	0
Oroxylum indicum (L.) Kurz i	0	1	0	0	0	0	0	0	0	0	0	0	2	0	0	0	0	0	0	0	0	0	0	0	0	0	0	0	0	0	0	0	0	0	0	0	0	0	0	0	0	0
Oryza sativa L.	1	0	0	0	0	0	0	0	0	0	0	0	0	0	0	0	0	0	0	0	0	0	0	0	0	0	0	0	0	0	0	0	0	0	0	0	0	1	0	0	0	0
Perilla setoyensis G.Honda	0	0	4	0	0	0	0	0	0	3	0	0	0	0	0	0	0	0	0	0	0	0	3	0	0	0	3	0	0	0	0	0	0	3	0	0	0	0	0	0	0	0
Phyllanthus amarus Schumach. & Thonn.	0	2	0	0	0	0	0	0	0	0	0	0	0	2	2	0	0	0	0	0	0	0	0	0	0	0	0	0	0	0	0	0	0	0	0	0	0	0	0	0	0	0
Phyllanthus emblica L.	0	8	0	0	0	0	0	0	0	0	0	0	2	2	2	0	0	0	0	0	0	2	2	0	0	2	2	0	0	0	0	0	2	0	0	0	0	0	0	0	0	0
Phyllanthus maderaspatensis L.	0	0	1	0	0	0	0	0	0	0	0	0	0	0	0	0	0	0	0	0	0	0	0	0	0	0	0	0	0	0	0	0	3	0	0	0	0	0	0	0	0	0
Phyllanthus niruri L.	0	3	0	0	0	0	0	2	0	0	0	2	0	0	0	0	0	0	0	0	0	0	0	0	0	0	0	0	0	0	0	0	2	0	0	0	0	0	0	0	0	0
Phyllanthus tenellus Roxb.	0	0	1	0	0	0	0	0	0	0	0	0	0	0	0	0	0	0	0	0	0	0	0	0	0	0	0	0	0	0	0	0	3	0	0	0	0	0	0	0	0	0
Phyllanthus urinaria L.	0	1	0	2	0	0	0	0	0	0	0	0	0	0	0	0	0	0	0	0	0	0	0	0	0	0	0	0	0	0	0	0	0	0	0	0	0	0	0	0	0	0
Picrorhiza kurroa Royle ex Benth.	0	3	0	0	0	0	0	2	0	0	0	2	0	0	0	0	0	0	0	0	0	0	0	0	0	0	0	0	0	0	0	0	0	0	0	0	0	2	0	0	0	0
Piper cubeba Bojer	0	1	0	2	0	0	0	0	0	0	0	0	0	0	0	0	0	0	0	0	0	0	0	0	0	0	0	0	0	0	0	0	0	0	0	0	0	0	0	0	0	0
Piper longum L.	3	3	2	0	2	0	0	0	0	0	0	2	0	0	2	0	0	0	0	0	0	0	1	3	1	1	0	0	0	0	0	0	0	0	3	0	0	0	0	0	0	0
Piper nigrum L.	3	0	1	0	0	0	0	0	0	0	0	0	0	0	1	0	0	0	0	0	0	0	1	3	1	0	0	0	0	0	0	0	0	0	0	0	0	0	0	0	0	0
Plantago major L.	0	0	1	0	0	0	0	0	0	0	0	0	0	0	0	0	0	0	0	0	0	0	0	0	0	0	3	0	0	0	0	0	0	0	0	0	0	0	0	0	0	0
Plantago ovata Forssk.	0	0	1	0	0	0	0	0	0	0	0	0	0	0	0	0	0	0	0	0	0	0	0	0	0	0	0	0	0	0	0	0	0	0	0	0	3	0	0	0	0	0
Plumbago zeylanica L.	0	1	0	0	0	0	0	0	0	0	0	0	0	2	0	0	0	0	0	0	0	0	0	0	0	0	0	0	0	0	0	0	0	0	0	0	0	0	0	0	0	0
Premna mollissima Roth	0	1	0	0	0	0	0	0	0	0	0	0	0	0	0	0	0	0	0	0	0	0	0	0	0	0	0	0	0	0	0	0	0	0	0	0	0	2	0	0	0	0
Prunus amygdalus Stokes	0	2	0	0	0	0	0	0	0	0	0	0	2	0	0	0	0	0	0	2	0	0	0	0	0	0	0	0	0	0	0	0	0	0	0	0	0	0	0	0	0	0
Pterocarpus marsupium Roxb.	0	2	0	0	2	0	0	0	0	0	0	0	0	0	2	0	0	0	0	0	0	0	0	0	0	0	0	0	0	0	0	0	0	0	0	0	0	0	0	0	0	0
Pterocarpus santalinus L.f.	0	1	0	0	0	0	0	0	0	0	0	0	0	0	0	0	0	0	0	0	0	0	0	0	0	0	0	0	0	0	0	0	0	2	0	0	0	0	0	0	0	0
Pueraria tuberosa (Willd.) DC.	0	2	0	0	0	2	0	0	0	0	0	0	0	0	0	0	0	0	0	0	0	0	0	0	0	0	0	0	0	0	0	0	0	0	0	0	0	2	0	0	0	0
Punica granatum L.	0	0	1	0	0	0	0	0	0	0	0	3	0	0	0	0	0	0	0	0	0	0	0	0	0	0	0	0	0	0	0	0	0	0	0	0	0	0	0	0	0	0
Raphanus sativus L	0	1	2	0	0	3	0	0	0	0	0	0	0	2	0	0	0	0	0	0	0	0	0	0	0	3	0	0	0	0	0	0	0	0	0	0	0	0	0	0	0	0
Rheum emodi L.	0	1	0	0	0	0	0	2	0	0	0	0	0	0	0	0	0	0	0	0	0	0	0	0	0	0	0	0	0	0	0	0	0	0	0	0	0	0	0	0	0	0
Rosa rugosa Thunb.	0	0	1	0	0	0	0	0	0	0	0	0	0	0	0	0	0	0	0	0	0	0	0	3	0	0	0	0	0	0	0	0	0	0	0	0	0	0	0	0	0	0
Rubia cordifolia L.	0	2	0	0	0	0	0	0	0	0	0	0	0	0	0	2	0	0	0	0	0	0	0	0	0	0	0	0	0	0	0	0	0	2	0	0	0	0	0	0	0	0
Rumex maritimus L.	0	1	0	0	0	0	0	0	0	0	0	0	0	0	2	0	0	0	0	0	0	0	0	0	0	0	0	0	0	0	0	0	0	0	0	0	0	0	0	0	0	0
Saccharum bengalense Retz.	0	1	0	0	0	0	0	0	0	0	0	0	0	0	0	0	0	0	0	0	0	0	0	0	0	0	0	0	0	0	0	0	0	0	0	0	0	2	0	0	0	0
Saccharum officinarum L.	0	1	0	0	0	0	0	0	0	0	0	0	0	0	0	0	0	0	0	0	0	0	0	0	0	0	0	0	0	0	0	0	0	0	0	0	0	2	0	0	0	0
Salvia plebeia R.Br.	0	0	3	0	0	0	0	0	0	0	0	0	0	0	0	0	0	0	0	3	0	0	0	0	0	0	0	0	0	0	0	0	0	0	3	3	0	0	0	0	0	0
Santalum album L.	0	1	0	0	0	0	0	0	0	0	0	0	0	0	0	0	0	0	0	0	0	0	0	2	0	0	0	0	0	0	0	0	0	0	0	0	0	0	0	0	0	0
Saraca asoca (Roxb.) Willd.	1	1	1	0	0	0	0	0	0	0	0	0	0	2	0	0	0	0	0	0	0	0	0	0	0	0	0	0	0	0	0	0	0	0	3	1	0	0	0	0	0	0
Saxifraga granulata L.	0	1	0	0	0	0	0	0	0	0	0	0	0	0	0	0	0	0	0	0	0	0	0	2	0	0	0	0	0	0	0	0	0	0	0	0	0	0	0	0	0	0
Saxifraga ligulata Murray	0	1	0	0	0	0	0	0	0	0	0	0	0	0	0	2	0	0	0	0	0	0	0	0	0	0	0	0	0	0	0	0	0	0	0	0	0	0	0	0	0	0
Secale cereale L.	0	0	1	0	0	0	0	0	0	0	0	0	0	0	0	0	0	0	0	0	0	0	0	0	0	0	0	0	0	0	0	0	0	0	0	0	0	0	0	0	3	0
Sedum japonicum Siebold ex Miq.	0	0	1	0	0	3	0	0	0	0	0	0	0	0	0	0	0	0	0	0	0	0	0	0	0	0	0	0	0	0	0	0	0	0	0	0	0	0	0	0	0	0
Senna alexandrina Mill.	0	0	6	3	0	0	0	3	0	0	0	3	0	0	0	0	0	0	0	0	3	0	0	3	0	0	0	0	0	0	0	0	0	0	0	0	0	3	0	0	0	0
Senna auriculata (L.) Roxb.	0	0	1	0	0	0	0	0	0	0	0	0	0	0	0	0	0	0	0	0	0	0	0	3	0	0	0	0	0	0	0	0	0	0	0	0	0	0	0	0	0	0
Senna occidentalis L.	0	1	1	0	0	0	0	0	3	0	0	0	0	2	0	0	0	0	0	0	0	0	0	0	0	0	0	0	0	0	0	0	0	0	0	0	0	0	0	0	0	0
Senna tora (L.) Roxb.	0	0	2	0	0	0	0	0	0	0	0	3	0	0	0	0	0	0	0	0	0	0	0	0	0	0	0	0	0	0	0	0	0	0	0	0	0	0	0	0	3	0
Sesamum indicum L.	0	0	1	0	0	0	0	0	0	0	0	0	0	0	0	0	0	0	0	0	0	0	0	0	0	0	0	0	0	0	0	0	0	0	0	0	0	3	0	0	0	0
Sida acuta Burm.f.	0	0	3	0	0	0	0	0	0	0	0	0	0	0	0	0	0	0	0	0	0	0	3	0	3	0	0	0	3	0	0	0	0	0	0	0	0	0	0	0	0	0
Sida cordifolia L.	2	1	0	0	0	0	0	0	0	0	0	0	0	0	0	0	2	0	0	0	0	0	0	0	0	0	0	0	1	0	0	0	0	0	0	0	0	1	0	0	0	0

Solanum indicum L.	0	1	0	0	0	0	0	0	0	0	0	0	0	0	0	0	0	0	0	0	0	0	0	0	0	0	0	0	0	0	0	0	0	0	0	0	0	2	0	0	0	0
Solanum lycopersicum L.	0	0	3	0	0	0	0	0	0	0	0	0	0	0	3	0	0	0	0	0	0	0	0	0	0	0	0	0	0	0	3	0	0	0	0	0	0	0	3	0	0	0
Solanum nigrum L.	0	1	0	0	0	0	0	0	0	0	0	0	0	2	0	0	0	0	0	0	0	0	0	0	0	0	0	0	0	0	0	0	0	0	0	0	0	0	0	0	0	0
Solanum surattense Burm. f.	0	1	0	0	0	0	0	0	0	0	0	0	0	0	0	0	0	0	0	0	0	0	0	0	0	0	0	0	0	0	0	0	0	0	0	0	0	2	0	0	0	0
Sorghum bicolor (L.) Moench	0	0	1	0	0	0	0	0	0	0	0	0	0	0	0	0	0	0	0	0	0	0	0	0	0	0	0	0	0	0	0	0	0	0	0	0	0	3	0	0	0	0
Sphaeranthus indicus L.	0	2	0	0	0	0	0	0	0	0	0	0	0	0	2	0	0	0	0	0	0	0	0	2	0	0	0	0	0	0	0	0	0	0	0	0	0	0	0	0	0	0
Swertia chirata Buch.-Ham. ex Wall.	0	2	0	0	0	0	0	0	0	0	0	0	0	0	2	0	0	0	0	0	0	0	0	0	0	2	0	0	0	0	0	0	0	0	0	0	0	0	0	0	0	0
Syzygium cumini (L.) Skeels	0	2	0	0	2	0	0	0	0	0	0	0	0	0	2	0	0	0	0	0	0	0	0	0	0	0	0	0	0	0	0	0	0	0	0	0	0	0	0	0	0	0
Tamarix gallica L	0	1	0	0	0	0	0	0	0	0	0	0	0	2	0	0	0	0	0	0	0	0	0	0	0	0	0	0	0	0	0	0	0	0	0	0	0	0	0	0	0	0
Taraxacum officinale (L.) Weber ex F.H.Wigg.	0	0	1	0	0	0	0	0	0	0	0	0	0	0	0	0	0	0	0	0	0	0	0	0	0	0	0	0	0	0	0	0	0	0	0	3	0	0	0	0	0	0
Tephrosia purpurea (L.) Pers.	0	1	0	0	0	0	0	2	0	0	0	0	0	0	0	0	0	0	0	0	0	0	0	0	0	0	0	0	0	0	0	0	0	0	0	0	0	0	0	0	0	0
Terminalia arjuna (Roxb. ex DC.) Wight & Arn.	1	5	0	0	0	0	0	0	0	0	0	0	2	2	0	0	0	0	0	0	0	0	0	0	1	0	2	0	0	0	2	0	0	0	0	0	0	0	0	0	0	2
Terminalia bellirica (Gaertn.) Roxb.	2	2	0	0	0	0	0	0	0	0	0	0	0	0	2	0	0	0	0	0	0	1	1	0	0	2	0	0	0	0	0	0	0	0	0	0	0	0	0	0	0	0
Terminalia chebula Retz.	0	7	0	0	0	0	0	0	0	0	0	0	2	2	2	0	2	0	0	0	0	2	2	0	0	2	0	0	0	0	0	0	0	0	0	0	0	0	0	0	0	0
Thysanolaena latifolia (Roxb. ex Hornem.) Honda	0	0	1	0	0	0	0	0	0	0	0	0	0	0	0	0	0	0	3	0	0	0	0	0	0	0	0	0	0	0	0	0	0	0	0	0	0	0	0	0	0	0
Tinospora cordifolia (Willd.) Miers	3	7	0	0	2	0	0	0	0	0	0	0	2	2	2	0	0	2	0	0	0	0	0	0	0	0	0	0	0	1	0	0	1	1	0	0	0	2	0	2	0	0
Trachyspermum ammi (L.) Sprague	1	1	0	0	0	0	0	0	0	0	0	0	0	0	0	0	0	0	0	0	0	0	0	2	0	1	0	0	0	0	0	0	0	0	0	0	0	0	0	0	0	0
Trianthema portulacastrum L.	0	0	3	0	0	0	0	0	0	0	0	0	0	0	0	0	0	0	0	0	0	0	0	0	0	0	0	0	3	0	0	3	0	0	0	0	3	0	0	0	0	0
Tribulus terrestris L.	0	4	6	0	0	0	0	0	0	0	0	3	0	0	2	3	0	2	2	2	0	0	0	3	3	3	0	0	3	0	0	0	0	0	0	0	0	0	0	0	0	0
Trigastrotheca molluginea F.Muell.	0	0	1	0	0	0	0	0	0	0	0	0	0	0	0	0	0	0	0	0	0	0	0	0	0	0	0	0	0	0	0	0	3	0	0	0	0	0	0	0	0	0
Trigonella foenum-graecum L.	2	1	1	0	1	3	0	0	2	0	0	0	0	0	0	0	0	0	0	0	0	0	0	0	0	1	0	0	0	0	0	0	0	0	0	0	0	0	0	0	0	0
Triticum aestivum L.	0	0	1	0	0	0	0	0	0	0	0	0	0	0	0	0	0	0	0	0	0	0	0	0	0	0	0	0	0	0	0	0	0	0	0	0	0	0	0	0	3	0
Valeriana officinalis L.	0	1	0	0	0	0	0	0	0	0	2	0	0	0	0	0	0	0	0	0	0	0	0	0	0	0	0	0	0	0	0	0	0	0	0	0	0	0	0	0	0	0
Valeriana wallichii DC.	0	1	0	0	0	0	0	0	0	0	0	0	2	0	0	0	0	0	0	0	0	0	0	0	0	0	0	0	0	0	0	0	0	0	0	0	0	0	0	0	0	0
Vernonia cinerea (L.) Less.	0	1	0	0	0	0	0	0	0	0	0	0	0	0	0	2	0	0	0	0	0	0	0	0	0	0	0	0	0	0	0	0	0	0	0	0	0	0	0	0	0	0
Vigna radiata (L.) R.Wilczek	0	0	1	0	0	0	0	0	0	0	0	0	0	0	0	0	0	0	0	0	0	0	0	0	3	0	0	0	0	0	0	0	0	0	0	0	0	0	0	0	0	0
Withania somnifera (L.) Dunal	4	1	6	0	0	1	1	3	1	0	0	3	2	0	0	0	0	3	0	3	0	0	0	3	0	0	0	0	0	0	0	0	0	0	0	0	0	3	1	0	0	0
Zingiber officinale Roscoe	3	3	0	0	0	0	0	0	0	0	0	0	1	0	2	2	0	0	0	0	0	0	2	0	1	1	0	0	0	0	0	0	0	0	0	0	0	0	0	0	0	0

**Total (presence of MOTU per category)**	**26**	**107**	**81**
**Total (sum of occurences of MOTU per category)**	**47**	**184**	**144**
