# Supplementary material for: DNA Metabarcoding Authentication of Ayurvedic Herbal Products on the European Market Raises Concerns of Quality and Fidelity
Source: Front Plant Sci. 2019 Feb 5;10:68. doi: 10.3389/fpls.2019.00068 (PMC6370972; doi:10.3389/fpls.2019.00068)
Supplement: Supplementary file 6 [file Data_Sheet_6.PDF]

Supplementary Table S6. Details of genomic DNA concentration, nrITS amplicon concentration and success rate of sequence yield in herbal products after filtering the raw sequences between nrITS1 and nrITS2, and among replicates, and number of MOTUs in nrITS1 and nrITS after NCBI-BLAST identification

Herbal products code	Herbal product type	Total genomic DNA concentration measured using Qubit 2.0 Fluorometer (ng/µl)	nrITS1 - Amplicon concentration of triplicates using qPCR*			nrITS2 - Amplicon concentration of triplicates using qPCR*			nrITS1 - Sequence yield in triplicates after filtering			nrITS2 - Sequence yield in triplicates after filtering			nrITS1 - No. of MOTU's in triplicates afte NCBI-BLAST identification			nrITS2 - No. of MOTU's in triplicates afte NCBI-BLAST identification		
			nrITS1 amplicon concentration of replicate 1 (ng/µl)	nrITS1 amplicon concentration of replicate 2 (ng/µl)	nrITS1 amplicon concentration of replicate 3 (ng/µl)	nrITS2 amplicon concentration of replicate 1 (ng/µl)	nrITS2 amplicon concentration of replicate 2 (ng/µl)	nrITS2 amplicon concentration of replicate 3 (ng/µl)	No. of reads in replicate1 (MOTU's)	No. of reads in replicate2 (MOTU's)	No. of reads in replicate3 (MOTU's)	No. of reads in replicate1 (MOTU's)	No. of reads in replicate2 (MOTU's)	No. of reads in replicate3 (MOTU's)	No. of MOTU's in replicate1 (MOTU's)	No. of MOTU's in replicate2 (MOTU's)	No. of MOTU's in replicate3 (MOTU's)	No. of MOTU's in replicate1 (MOTU's)	No. of MOTU's in replicate2 (MOTU's)	No. of MOTU's in replicate3 (MOTU's)
1	Tablets	2,23	4,1	6,94	0,65	9,83	3,23	32,53	0	1237	3262	3126	0	3265	0	5	6	4	0	7
2	Tablets	3,3	6,1	5,66	0,85	6,02	10,52	27,68	0	1198	9656	452	979	3619	0	4	2	1	1	1
3	Tablets	1,24	0	10,79	63,45	5,56	15,77	42,95	0	629	1033	0	2470	148	0	10	11	0	3	3
4	Tablets	2,75	2,97	0,51	4,2	4,27	4,96	14,41	0	0	0	0	352	18	0	0	0	0	3	1
5	Capsules	0,85	2,15	0,27	5,92	3,84	0,01	18,68	0	0	0	0	18	20	0	0	0	0	1	1
6	Tablets	1,55	5,13	1,69	2,45	3,46	3,96	10,1	1503	2533	1370	0	680	1980	2	5	2	0	7	15
7	Capsules	24,6	5,35	4,39	3,3	3,63	8,55	5,87	2076	1997	1547	6291	750	7680	4	4	3	2	7	7
8	Capsules	0,7	4,66	5,04	6,11	1,35	0	0,01	123	19	0	0	0	0	1	1	0	0	0	0
9	Capsules	0,88	0,02	0,74	4,53	4,29	5,38	15,57	31862	10017	1907	0	281	914	1	1	1	0	2	7
10	Capsules	0,87	3,44	0,3	12,63	4,68	4,46	4,59	1297	1540	24523	140	1630	7793	1	4	2	1	8	13
11	Capsules	0,82	0	0	0	0,05	0	0	0	0	0	0	0	0	0	0	0	0	0	0
12	Tablets	1,95	1,7	0,21	52,18	1,8	2,56	4,61	0	0	0	0	14	56	0	0	0	0	1	3
13	Tablets	1,9	1,42	0,4	6,15	9,87	4,06	7,76	0	1499	781	0	319	821	0	1	3	0	1	5
14	Tablets	2,54	1,99	0,89	1,68	6,81	7,26	29,03	14461	6414	34720	0	0	0	4	3	2	0	0	0
15	Capsules	1,9	0,86	0,36	3,09	4,02	4,01	0,01	0	0	0	0	35	73	0	0	0	0	2	2
16	Tablets	1,27	4,84	3,18	1,16	3,87	0,52	0,69	14319	9989	3982	0	0	0	2	2	6	0	0	0
17	Tablets	2,45	1,85	1,17	20,55	2,66	3,36	11,16	8270	819	910	0	198	301	4	1	3	0	3	4
18	Tablets	6,09	8	3,78	0,58	3,34	3,1	8,97	589	6054	1336	0	1284	678	2	3	2	0	4	2
19	Tablets	1,3	0,22	0	26,16	2,16	6,87	4,52	347	0	0	0	3102	5815	3	0	0	0	1	3
20	Tablets	0,84	0	0	0	0,01	0	0	0	0	0	0	0	0	0	0	0	0	0	0
21	Tablets	1,89	5,5	1,81	0,17	15,19	3,78	21,29	0	0	0	0	0	0	0	0	0	0	0	0
22	Tablets	1,2	0,01	0	0	3,74	0,65	1,29	0	0	0	0	0	0	0	0	0	0	0	0
23	Tablets	3,04	3,07	2,84	3,03	9,45	4,05	15,76	5862	1934	102	0	91	679	3	23	2	0	4	6
24	Extracts	0,17	3,78	1,6	1,27	15,01	4,57	3,38	53	0	0	0	14	91	0	0	0	0	1	2
25	Extracts	0,13	3,27	1,46	4,16	34,67	8,59	18,33	50	0	171	0	0	36	1	0	1	0	0	1
26	Extracts	1,1	3,09	4,05	1,67	11,17	12,32	41,32	0	889	2142	0	0	93	0	1	2	0	0	1
27	Capsules	14,6	5,02	4,49	0,46	8,18	11,32	16,75	4744	1934	11127	1310	652	3428	11	4	10	3	5	6
28	Capsules	0,16	6,05	6,26	0,56	30,22	0	28,14	0	0	0	0	0	0	0	0	0	0	0	0
29	Capsules	4,88	0	0	7,29	5,92	1,36	7,1	0	0	0	0	0	0	0	0	0	0	0	0
30	Capsules	0,27	3,85	0,33	2	2,43	0,83	27,87	2850	6146	10418	0	0	0	1	2	1	0	0	0
31	Tablets	4,56	6,79	0,9	4,04	1,16	1,33	2,15	689	2354	3485	152	0	24	7	5	7	1	0	1
32	Tablets	3,12	36,55	8,8	1,99	9,28	4,79	47,4	3744	2950	3781	3247	2166	3602	4	5	4	3	15	11
33	Tablets	5,4	7,8	8,23	0,36	52,2	17,14	75,42	0	0	0	0	0	0	0	0	0	0	0	0
34	Tablets	18,6	8,48	11,15	1,43	28,62	3,73	12,14	5764	1437	467	2455	4355	2169	10	8	4	6	8	14
35	Tablets	10,7	4,59	2,65	0,01	2,37	11,62	38,1	0	0	0	0	0	0	0	0	0	0	0	0
36	Tablets	23,3	7,67	10,53	0,74	10,05	2,56	6,65	4821	2568	1063	13630	29	70	7	6	5	1	1	1
37	Tablets	5,19	6,23	3,78	3,72	4,94	3,99	0	0	0	0	0	0	0	0	0	0	0	0	0
38	Tablets	3,13	0	0	0	0	0	0	0	0	0	0	0	0	0	0	0	0	0	0
39	Tablets	6,07	3,59	3,07	0,07	11,49	13,02	33,58	0	0	0	0	0	0	0	0	0	0	0	0
40	Capsules	5,33	2,7	1,37	1,59	4,59	4,02	2,4	193	21	61	3007	967	1105	3	2	2	4	6	9
41	Capsules	15,6	0,03	1,21	7,37	1,86	0,42	0	0	0	0	0	0	0	0	0	0	0	0	0
42	Capsules	43,6	0	0	0	0,14	0,11	0,9	0	0	0	0	0	0	0	0	0	0	0	0
43	Capsules	0,22	0	0,02	0	19,62	4,11	16,05	0	0	0	0	0	0	0	0	0	0	0	0
44	Capsules	31,2	7,56	28,99	1,64	8,04	22,89	53,43	0	0	0	0	0	0	0	0	0	0	0	0
45	Capsules	6	7,42	4,37	0,96	31,28	4,86	0	0	0	0	0	0	0	0	0	0	0	0	0
46	Capsules	13,9	0	0	0	0	0	0	0	0	0	0	0	0	0	0	0	0	0	0
47	Capsules	31,8	14,68	4,47	0,51	28,84	7,76	0	0	0	0	0	0	0	0	0	0	0	0	0
48	Powders	19,4	0	0	0	0	0	0	0	0	0	0	0	0	0	0	0	0	0	0
49	Powders	19,4	0	0	12,72	1,06	0,33	0,91	0	0	0	0	0	0	0	0	0	0	0	0
50	Powders	4,83	0	0	0	2,7	1,28	0	0	0	0	0	0	0	0	0	0	0	0	0
51	Powders	19,2	29,98	0,01	0,61	24,41	0	54,42	0	0	0	0	0	0	0	0	0	0	0	0
52	Powders	42,5	7,92	0,59	2,95	3,52	3,28	9,7	539	0	12	1389	6616	78299	1	0	1	1	1	2
53	Powders	190	7,27	1,96	1,11	6,59	1,93	5,26	0	0	0	0	0	0	0	0	0	0	0	0
54	Powders	21	82,98	24,36	0,2	39,08	45,5	0	0	0	0	0	0	0	0	0	0	0	0	0
55	Powders	210	14,05	0,48	1,34	1,9	1,58	3,55	4115	1814	366	1994	15	178	6	4	3	5	1	4
56	Powders	3,31	32,61	2,09	6,76	0,26	4,52	49,64	0	0	0	0	0	0	0	0	0	0	0	0
57	Powders	50	5,36	5,62	0,78	0,13	1,35	0,09	0	0	0	0	0	0	0	0	0	0	0	0
58	Powders	22,3	28,88	0,48	0,35	0,14	0,32	0,06	423	47	180	128	421	181	2	1	2	1	2	3
59	Capsules	0,14	4,43	2,95	1,12	0,15	0,33	0,14	0	0	0	0	0	0	0	0	0	0	0	0
60	Capsules	0,07	1,46	50,22	48,45	0,49	1,64	0,24	13440	0	0	0	46	151	1	0	0	0	1	2
61	Capsules	5,38	0,89	20,47	16,42	2,07	3,2	0,83	39152	12493	28925	102	885	1115	7	8	6	1	2	5
62	Powders	0,05	3,67	12,31	4,6	0,69	0,1	0	0	0	0	0	0	0	0	0	0	0	0	0
63	Capsules	0,17	5,48	0,98	0,12	0,33	0,55	Not Applicable	1117	30	244	0	38	3547	4	1	2	0	6	7
64	Capsules	24,5	56,27	1,58	0,59	0,35	2,7	1,8	0	0	0	0	0	0	0	0	0	0	0	0
65	Capsules	11,8	6,35	6,17	2,69	2,1	7,42	2,17	0	0	0	0	0	0	0	0	0	0	0	0
66	Capsules	15,6	0,65	15,07	12,27	1,36	7,52	1,33	42	32	62	119	812	809	2	2	2	1	3	4
67	Capsules	22,4	0,09	14,08	5,47	2,62	14,1	30,7	0	0	0	0	0	0	0	0	0	0	0	0
68	Capsules	3,19	20,55	1,51	3,81	0,15	1,93	0,18	1157	6181	13066	263	670	1549	4	5	4	1	10	7
69	Capsules	3,35	10,12	1,9	1,12	0,57	6,38	4,21	1681	5117	6291	9577	8674	15737	6	12	15	4	3	5
70	Capsules	3,9	16,12	1,38	3,29	0,52	1,57	0,36	2022	40823	73348	778	0	1358	1	4	3	4	0	3
71	Tablets	6,85	12,63	7,88	2,58	0,16	1,47	0,13	0	0	0	0	0	0	0	0	0	0	0	0
72	Tablets	10,8	2,49	15,54	23,59	1,44	2,4	2,03	0	0	0	0	0	0	0	0	0	0	0	0
73	Tablets	7,39	2,81	1,51	3,16	1,14	2,47	6,73	2859	3030	73348	6511	4424	1241	12	14	3	8	17	18
74	Tablets	17,5	3,63	2,88	1,6	1,19	3,57	0,16	46	205	33205	1192	5048	4268	2	1	5	1	4	6
75	Tablets	16,9	0,68	13,02	3,49	1,71	3,12	1,05	0	0	174	569	738	655	0	0	5	1	2	3
76	Powders	54	7,85	0,88	1,11	68,22	1,97	0,95	0	0	0	0	0	0	0	0	0	0	0	0
77	Powders	6,3	68,41	0,52	0,54	0,45	0,49	Not Applicable	1283	2796	267	597	1646	829	2	3	3	1	5	8
78	Powders	53	12,42	0,59	0,39	0,13	0,4	0,3	0	0	0	0	0	0	0	0	0	0	0	0
79	Powders	0,05	5,68	4,98	4,71	0,45	1	0,32	60	1068	14	0	148	273	2	3	1	0	3	1
Negative Control 1		0	0	0	0	0	0	0	0	0	0	0	0	0	0	0	0	0	0	0
Negative Control 2		0	0	0	0	0	0	0	0	0	0	0	0	0	0	0	0	0	0	0
Negative Control 3		0	0	0	0	0	0	0	0	0	0	0	0	0	0	0	0	0	0	0

Notes. *Values that correspond to '0' in amplicon concentration represents that the qPCR measured as '0' which implies that it has no amplification. In such cases, the complete volume from PCR well were taken for normalization and sequencing. The sample represented as 'Not Applicable' is that qPCR results have not given any values for those samples, in such cases Gel Images were compared and the samples were normalized based on the intensity of the DNA band in the Gel.
