# Supplementary material for: DNA Metabarcoding Authentication of Ayurvedic Herbal Products on the European Market Raises Concerns of Quality and Fidelity
Source: Front Plant Sci. 2019 Feb 5;10:68. doi: 10.3389/fpls.2019.00068 (PMC6370972; doi:10.3389/fpls.2019.00068)
Supplement: Supplementary file 7 [file Data_Sheet_7.PDF]

Supplementary Table S7. Results of the DNA metabarcode analysis of herbal products

Herbal product number	Product type	Number of species as per label	Scientific names of the plant ingredients as indicated on the product label	Availability of nrITS sequences in GenBank	Species detected by DNA metabarcoding	Number of reads (nrITS1)			Number of reads (nrITS2)			Number of MOTU's (nrITS1)			Number of MOTU's (nrITS2)			Species identified in the product by DNA metabarcoding (Critieria of the identiy: a species was considered and validated as being present within the product only if it was detected in at least 2 out of the 3 replicates)#			Does nrITS sequence of the plant species in the product label found by DNA metabarcoding?	Total number of plant species detected within the product by DNA metabarcoding	Total number of species listed on the product label detected by DNA metabarcoding	Fidality rate per herbal product
						Replicate 1	Replicate 2	Replicate 3	Replicate 1	Replicate 2	Replicate 3	Replicate 1	Replicate 2	Replicate 3	Replicate 1	Replicate 2	Replicate 3	Detected species by applying our quality criteria selection#	nrITS1 Detection in replicates 1+2+3	nrITS2 Detection in replicates 1+2+3				
1	Tablets	7	Acacia arabica (Lam.) Willd. (= Acacia nilotica (L.) Delile)	Yes		0	0	0	0	0	0	0	5	6	4	0	7				No	6	0	0 %
			Achyranthes aspera L.	Yes		0	0	0	0	0	0										No			
			Bergenia ligulata Engl.	Yes		0	0	0	0	0	0										No			
			Boerhaavia diffusa L.	Yes		0	0	0	0	0	0										No			
			Crateva nurvala Buch.-Ham	Genus is represented		0	0	0	0	0	0										No			
			Phyllanthus urinaria L.	Yes		0	0	0	0	0	0										No			
			Piper cubeba Bojer	Yes		0	0	0	0	0	0										No			
					Cassia sp.	0	0	0	0	0	31													
					Chamaecrista olesiphylla (Vogel) H.S.Irwin & Barneby	0	0	0	117	0	131							Chamaecrista olesiphylla (Vogel) H.S.Irwin & Barneby	0	117+0+131				
					Convolvulus prostratus Forssk.	0	20	44	0	0	0							Convolvulus prostratus Forssk.	0+20+44	0				
					Coriandrum sativum L.	0	19	0	0	0	58													
					Cuminum cyminum L.	0	0	0	335	0	65							Cuminum cyminum L.	0	335+0+65				
					Foeniculum vulgare Mill.	0	0	15	0	0	0													
					Hibiscus syriacus L.	0	65	11	0	0	0							Hibiscus syriacus L.	0+65+11	0				
					Ipomoea parasitica (Kunth) G. Don	0	707	2972	963	0	1446							Ipomoea parasitica (Kunth) G. Don	0+707+2972	963+0+1446				
					Plantago afra L.	0	0	14	0	0	0													
					Senna alexandrina Mill.	0	426	206	1711	0	1357							Senna alexandrina Mill.	0+426+206	1711+0+1357				
					Withania somnifera (L.) Dunal	0	0	0	0	0	177													
2	Tablets	11	Azadirachta indica A.Juss.	Yes		0	0	0	0	0	0	0	4	2	1	1	1				No	2	1	9 %
			Acacia arabica (Lam.) Willd. (= Acacia nilotica (L.) Delile)	Yes		0	0	0	0	0	0										No			
			Curcuma longa L.	Yes		0	0	0	0	0	0										No			
			Enicostemma littorale Blume	No (other genus are represented)		0	0	0	0	0	0										No			
			Gymnema sylvestre (Retz.) R.Br. ex Sm.	Yes		0	0	0	0	0	0										No			
			Momordica charantia L.	Yes		0	0	0	0	0	0										No			
			Piper longum L.	Yes		0	0	0	0	0	0										No			
			Pterocarpus marsupium Roxb.	ITS2 and Genus is represented		0	0	0	0	0	0										No			
			Syzygium cumini (L.) Skeels	Yes		0	0	0	0	0	0										No			
			Tinospora cordifolia (Willd.) Miers	Yes		0	0	0	0	0	0										No			
			Trigonella foenum-graecum L.	Yes		0	168	940	0	0	0							Trigonella foenum-graecum L.	0+168+940	0	Yes			
					Ipomoea nil (L.) Roth	0	39	0	0	0	0													
					Ipomoea parasitica (Kunth) G. Don	0	980	8716	452	979	3619							Ipomoea parasitica (Kunth) G. Don	0+980+8716	452+979+3619				
					Murraya koenigii (L.) Spreng.	0	11	0	0	0	0													
3	Tablets	4	Acacia arabica (Lam.) Willd. (= Acacia nilotica (L.) Delile)	Yes		0	0	0	0	0	0	0	10	11	0	3	3				No	9	1	25 %
			Asparagus racemosus Willd.	Yes		0	0	0	0	0	0										No			
			Chlorophytum arundinaceum Baker	Yes		0	0	0	0	0	0										No			
			Withania somnifera (L.) Dunal	Yes		0	0	0	0	2294	24							Withania somnifera (L.) Dunal	0	0+2294+24	Yes			
					Azadirachta indica A.Juss.	0	51	0	0	0	0													
					Brassica nigra (L.) K.Koch	0	0	17	0	0	0													
					Cichorium intybus L.	0	188	157	0	156	91							Cichorium intybus L.	0+188+157	0+156+91				
					Cullen corylifolium (L.) Medik.	0	23	11	0	0	0							Cullen corylifolium (L.) Medik.	0+23+11	0				
					Foeniculum vulgare Mill.	0	14	43	0	0	0							Foeniculum vulgare Mill.	0+14+43	0				
					Hygrophila schulli M.R.Almeida & S.M.Almeida	0	0	82	0	0	0													
					Ipomoea parasitica (Kunth) G. Don	0	183	214	0	20	33							Ipomoea parasitica (Kunth) G. Don	0+183+214	0+20+33				
					Momordica charantia L.	0	50	71	0	0	0							Momordica charantia L.	0+50+71	0				
					Plantago ovata Forssk.	0	12	0	0	0	0													
					Raphanus sativus L	0	33	19	0	0	0							Raphanus sativus L	0+33+19	0				
					Sedum japonicum Siebold ex Miq.	0	10	23	0	0	0							Sedum japonicum Siebold ex Miq.	0+10+23	0				
					Trigonella foenum-graecum L.	0	65	386	0	0	0							Trigonella foenum-graecum L.	0+65+386	0				
					Zea mays L.	0	0	10	0	0	0													
5	Capsules	1	Withania somnifera (L.) Dunal	Yes		0	0	0	0	18	24	0	0	0	0	0	1	Withania somnifera (L.) Dunal	0	0+18+24	Yes	1	1	100 %
6	Tablets	9	Aloe barbadensis Mill. (= Aloe vera (L.) Burm.f.)*	Yes		0	0	0	0	0	0	2	5	2	0	7	15				No	7	1	11 %
			Boerhaavia diffusa L.*	Yes		0	0	0	0	0	0										No			
			Capparis spinosa L.*	Yes		0	0	0	0	0	0										No			
			Cichorium intybus L.*	Yes		0	0	0	0	199	620							Cichorium intybus L.	0	0+199+620	Yes			
			Eclipta alba (L.) Hassk. (= Eclipta prostrata (L.) L.)	Yes		0	0	0	0	0	0										No			
			Phyllanthus niruri L.	Yes		0	0	0	0	0	0										No			
			Picrorhiza kurroa Royle ex Benth.*	Yes		0	0	0	0	0	0										No			
			Rheum emodi L.*	Yes		0	0	0	0	0	0										No			
			Tephrosia purpurea (L.) Pers.*	Yes		0	0	0	0	0	0										No			
					Cinnamomum iners Reinw. ex Blume	0	0	0	0	0	59													
					Coriandrum sativum L.	0	0	0	0	29	58							Coriandrum sativum L.	0	0+29+58				
					Cuminum cyminum L.	0	0	0	0	18	41							Cuminum cyminum L.	0	0+18+41				
					Echium vulgare L.	0	0	0	0	0	25													
					Embelia tsjeriam-cottam (Roem. & Schult.) A.DC.	0	37	0	0	0	0													
					Foeniculum vulgare Mill.	0	2265	380	0	0	0							Foeniculum vulgare Mill.	0+2265+380	0				
					Ipomoea parasitica (Kunth) G. Don	0	0	0	0	0	43													
					Jatropha curcas L.	0	0	0	0	0	33													
					Momordica charantia L.	0	0	0	0	0	53													
					Musa laterita Cheesman	50	0	0	0	0	0													
					Parthenium hysterophorus L.	0	0	0	0	0	49													
					Punica granatum L.	0	0	0	0	0	51													
					Senna alexandrina Mill.	1453	94	990	0	184	247							Senna alexandrina Mill.	1453+94+990	0+184+247				
					Trachyspermum ammi (L.) Sprague	0	125	0	0	0	50													
					Tribulus terrestris L.	0	0	0	0	15	173							Tribulus terrestris L.	0	0+15+173				
					Trigonella foenum-graecum L.	0	12	0	0	0	0													
					Triticum aestivum L.	0	0	0	0	10	0													
					Withania somnifera (L.) Dunal	0	0	0	0	225	422							Withania somnifera (L.) Dunal	0	0+225+422				
					Zea mays L.	0	0	0	0	0	56													
7	Capsules	6	Allium sativum L.	Yes		0	0	0	0	0	0	4	4	3	2	3	7				No	5	2	33 %
			Asparagus racemosus Willd.	Yes		0	0	0	0	13	10							Asparagus racemosus Willd.	0	0+13+10	Yes			
			Leptadenia reticulata (Retz.) Wight & Arn.	Yes		0	0	0	0	0	0										No			
			Pueraria tuberosa (Willd.) DC.	Yes		0	0	0	0	0	0										No			
			Trigonella foenum-graecum L.	Yes		0	0	0	0	0	0										No			
			Withania somnifera (L.) Dunal	Yes		0	0	14	0	50	261							Withania somnifera (L.) Dunal	0	0+50+261	Yes			
					Calluna vulgaris (L.) Hull	91	128	284	0	0	0							Calluna vulgaris (L.) Hull	91+128+284	0				
					Cichorium intybus L.	0	0	0	0	0	37													
					Coccinia sessilifolia (Sond.) Cogn.	0	11	0	0	0	0													
					Ipomoea parasitica (Kunth) G. Don	386	350	0	219	0	14							Ipomoea parasitica (Kunth) G. Don	386+350+0	219+0+14				
					Plukenetia loretensis Ule	34	0	0	0	0	0													
					Senna occidentalis (L.) Link	1565	1508	1249	6072	687	7314							Senna occidentalis (L.) Link	1565+1508+1249	6072+687+7314				
					Solanum nigrum L.	0	0	0	0	0	32													
					Trachyspermum ammi (L.) Sprague	0	0	0	0	0	12													
8	Capsules	2	Albizia lebbeck (L.) Benth.	Genus is represented		0	0	0	0	0	0	1	1	0	0	0	0				No	1	0	0 %
			Curcuma longa L.	Yes		0	0	0	0	0	0										No			
					Perilla setoyensis G.Honda	123	19	0	0	0	0							Perilla setoyensis G.Honda	123+19+0	0				
9	Capsules	2	Humulus lupulus L.	Yes		0	0	0	0	0	0	1	1	1	0	2	7				No	3	0	0 %
			Valeriana officinalis L.	Yes		0	0	0	0	0	0										No			
					Cannabis sativa L.	31862	10017	1907	0	0	0							Cannabis sativa L.	1862+10017+190	0				
					Cassia sp.	0	0	0	0	0	18													
					Cichorium intybus L.	0	0	0	0	74	402							Cichorium intybus L.	0	0+74+402				
					Cucumis sativus L.	0	0	0	0	0	13													
					Echinacea purpurea (L.) Moench	0	0	0	0	0	83													
					Ocimum basilicum L.	0	0	0	0	207	164							Ocimum basilicum L.	0	0+207+164				
					Ocimum tenuiflorum L.	0	0	0	0	0	133													
					Plantago ovata Forssk.	0	0	0	0	0	101													
10	Capsules	7	Bacopa monnieri (L.) Wettst.	Yes		0	0	0	0	0	0	1	4	2	1	8	13				No	6	0	0 %
			Boerhaavia diffusa L.	Yes		0	0	0	0	0	0										No			
			Camellia sinensis (L.) Kuntze	Yes		0	0	0	0	0	0										No			
			Eclipta alba (L.) Hassk. (= Eclipta prostrata (L.) L.)	Yes		0	0	0	0	0	0										No			
			Phyllanthus niruri L.	Yes		0	0	0	0	0	0										No			
			Picrorhiza kurroa Royle ex Benth.	Yes		0	0	0	0	0	0										No			
			Piper longum L.	Yes		0	0	0	0	0	0										No			
					Anchusa italica Retz.	0	0	0	0	66	164							Anchusa italica Retz.	0	0+66+164				
					Asparagus racemosus Willd.	0	0	0	0	0	32													
					Cinnamomum iners Reinw. ex Blume	0	0	0	0	0	46													
					Convolvulus microphyllus Sieber ex Spreng.	0	0	0	0	0	1612													
					Convolvulus prostratus Forssk.	0	0	24127	0	56	0													
					Cuminum cyminum L.	0	0	0	0	0	23													
					Foeniculum vulgare Mill.	0	221	0	0	0	0													
					Greeniopsis sibuyanensis Elmer	0	21	0	0	0	0													
					Lactuca sativa L.	0	0	0	0	0	11													
					Mentha canadensis L.	0	0	0	0	0	33													
					Punica granatum L.	1237	0	0	0	26	328							Punica granatum L.	0	0+26+328				
					Senna alexandrina Mill.	0	0	396	0	849	3642							Senna alexandrina Mill.	0	0+849+3642				
					Senna tora (L.) Roxb.	0	0	0	0	47	301							Senna tora (L.) Roxb.	0	0+47+301				
					Solanum lycopersicum L.	0	30	0	0	0	0													
					Strychnos nux-vomica L.	0	0	0	0	15	0													
					Trachyspermum ammi (L.) Sprague	0	0	0	0	0	62													
					Tribulus terrestris L.	0	0	0	0	143	21							Tribulus terrestris L.	0	0+143+21				
					Withania somnifera (L.) Dunal	0	1268	0	140	428	1518							Withania somnifera (L.) Dunal	0	140+428+1518				
12	Tablets	22	Acorus calamus L.*	Yes		0	0	0	0	0	0	0	0	0	0	1	3				No	1	1	13 %
			Anethum sowa Roxb. ex Fleming	Genus is represented		0	0	0	0	0	0										No			
			Bacopa monnieri (L.) Wettst.*	Yes		0	0	0	0	0	0										No			
			Bacopa monnieri (L.) Wettst.	Yes		0	0	0	0	0	0										No			
			Celastrus paniculatus Willd.*	Yes		0	0	0	0	0	0										No			
			Centella asiatica (L.) Urb. *	Yes		0	0	0	0	0	0										No			
			Elettaria cardamomum (L.) Maton	Yes		0	0	0	0	0	0										No			
			Embelia ribes Burm.f.*	Yes		0	0	0	0	0	0										No			
			Emblica officinalis Gaertn. (= Phyllanthus emblica L.)*	Yes		0	0	0	0	0	0										No			
			Evolvulus alsinoides (L.) L.*	Yes		0	0	0	0	0	0										No			
			Ipomoea digitata L. (= Ipomoea cheirophylla O'Donell)	Genus is represented		0	0	0	0	0	0										No			
			Mucuna pruriens (L.) DC.	Yes		0	0	0	0	0	0										No			
			Nardostachys jatamansi*	Yes		0	0	0	0	0	0										No			
			Oroxylum indicum (L.) Kurz i*	Yes		0	0	0	0	0	0										No			
			Prunus amygdalus Stokes*	Yes		0	0	0	0	0	0										No			
			Terminalia arjuna (Roxb. ex DC.) Wight & Arn.	Yes		0	0	0	0	0	0										No			
			Terminalia chebula Retz.*	Yes		0	0	0	0	0	0										No			
			Tinospora cordifolia (Willd.) Miers *	Yes		0	0	0	0	0	0										No			
			Valeriana wallichii DC. (= Valeriana jatamansi Jones)*	Yes		0	0	0	0	0	0										No			
			Withania somnifera (L.) Dunal*	Yes		0	0	0	0	0	0										No			
			Withania somnifera (L.) Dunal	Yes		0	0	0	0	0	0										No			
			Zingiber officinale Roscoe	Yes		0	0	0	0	14	15							Zingiber officinale Roscoe	0	0+14+15	Yes			
					Tribulus terrestris L.	0	0	0	0	0	16													
					Triticum aestivum L.	0	0	0	0	0	25													
			Achillea millefolium L.*	Yes		0	0	0	0	0	0										No			
			Boerhaavia diffusa L.	Yes		0	0	0	0	0	0										No			
			Capparis spinosa L.	Yes		0	0	0	0	0	0										No			
			Cassia occidentalis L. (= Senna occidentalis (L.) Link)*	Yes		0	0	0	0	0	0										No			
			Cichorium intybus L.	Yes		0	1499	76	0	319	440							Cichorium intybus L.	0+1499+76	0+319+440	Yes			
			Embelia ribes Burm.f.	Yes		0	0	0	0	0	0										No			

13	Tablets	16	*Emblica officinalis Gaertn. (= Phyllanthus emblica L.)*	Yes		0	0	0	0	0	0	0	1	3	0	1	5				No	1	1	9 %
			Fumaria officinalis L.	Yes		0	0	0	0	0	0										No			
			Phyllanthus amarus Schumach. & Thonn.	Yes		0	0	0	0	0	0										No			
			Plumbago zeylanica L.	Yes		0	0	0	0	0	0										No			
			Raphanus sativus L. (= Raphanus raphanistrum subsp. sativus (L.) Domin)	Yes		0	0	0	0	0	0										No			
			Solanum nigrum L.*	Yes		0	0	0	0	0	0										No			
			Tamarix gallica L.*	Yes		0	0	0	0	0	0										No			
			Terminalia arjuna (Roxb. ex DC.) Wight & Arn.*	Yes		0	0	0	0	0	0										No			
			Terminalia chebula Retz.	Yes		0	0	0	0	0	0										No			
			Tinospora cordifolia (Willd.) Miers	Yes		0	0	0	0	0	0										No			
					Convolvulus prostratus Forssk.	0	0	675	0	0	0													
					Echinacea purpurea (L.) Moench	0	0	0	0	0	150													
					Eclipta prostrata (L.) L.	0	0	0	0	0	110													
					Lactuca sativa L.	0	0	0	0	0	62													
					Phyllanthus maderaspatensis L.	0	0	30	0	0	59													
14	Tablets	27	*Abutilon indicum* (L.) Sweet	Yes		0	0	0	0	0	0	4	3	2	0	0	0				No	3	1	10 %
			*Aloe barbadensis Mill. (= Aloe vera (L.) Burm.f.)**	Yes		0	0	0	0	0	0										No			
			Asparagus racemosus Willd.*	Yes		0	0	0	0	0	0										No			
			Berberis aristata DC.*	Yes		0	0	0	0	0	0										No			
			Boerhaavia diffusa L.*	Yes		0	0	0	0	0	0										No			
			Casearia esculenta Roxb.*	Genus is represented		0	0	0	0	0	0										No			
			Curcuma longa L.	Yes		0	0	0	0	0	0										No			
			Emblica officinalis Gaertn. (= Phyllanthus emblica L.)	Yes		0	0	0	0	0	0										No			
			*Eugenia jambolana Lam. (= Syzygium cumini (L.) Skeels)**	Yes		0	0	0	0	0	0										No			
			Glycyrrhiza glabra L.*	Yes		0	0	0	0	0	0										No			
			Gmelina arborea Roxb.*	Yes		0	0	0	0	0	0										No			
			Gossypium herbaceum L.*	Yes		0	0	0	0	0	0										No			
			Gymnema sylvestre (Retz.) R.Br. ex Sm.*	Yes		0	0	0	0	0	0										No			
			Momordica charantia L.	Yes		0	0	0	0	0	0										No			
			Ocimum tenuiflorum L.	Yes		0	0	0	0	0	0										No			
			Phyllanthus amarus Schumach. & Thonn.*	Yes		0	0	0	0	0	0										No			
			Piper longum L.	Yes		0	0	0	0	0	0										No			
			Piper nigrum L.	Yes		5745	4913	21465	0	0	0							*Piper nigrum* L.	745+4913+2146	0	Yes			
			Pterocarpus marsupium Roxb.*	Yes		0	0	0	0	0	0										No			
			Rumex maritimus L.	Genus is represented		0	0	0	0	0	0										No			
			Sphaeranthus indicus L.*	Yes		0	0	0	0	0	0										No			
			Swertia chirata Buch.-Ham. ex Wall.*	Yes		0	0	0	0	0	0										No			
			Terminalia bellirica (Gaertn.) Roxb.	Yes		0	0	0	0	0	0										No			
			Terminalia chebula Retz.	Yes		0	0	0	0	0	0										No			
			Tinospora cordifolia (Willd.) Miers*	Yes		0	0	0	0	0	0										No			
			Tribulus terrestris L.*	Yes		0	0	0	0	0	0										No			
			Zingiber officinale Roscoe*	Yes		0	0	0	0	0	0										No			
					Brassica oleracea L.	8093	0	13185	0	0	0							*Brassica oleracea* L.	8093+0+13185	0				
					Coriandrum sativum L.	504	0	0	0	0	0													
					Medicago sativa L.	0	0	0	0	0	0													
					Solanum lycopersicum L.	119	0	70	0	0	0							*Solanum lycopersicum* L.	119+0+70	0				
					Veronica chamaedrys L.	0	1501	0	0	0	0													
16	Tablets	8	*Achyranthes aspera* L.	Yes		0	0	0	0	0	0	2	2	6	0	0	0				No	2	0	0 %
			Cyperus scariosus R.Br.	Genus is represented		0	0	0	0	0	0										No			
			Didymocarpus pedicellata (Ait.) Ait. F.	Genus is represented		0	0	0	0	0	0										No			
			Onosma bracteatum Wall	Genus is represented		0	0	0	0	0	0										No			
			Rubia cordifolia L.	Yes		0	0	0	0	0	0										No			
			Saxifraga ligulata Murray (= Saxifraga stolonifera Curtis)	Yes		0	0	0	0	0	0										No			
			Vernonia cinerea (L.) Less. (= Cyanthillium cinereum (L.) H.Rob.)	Yes		0	0	0	0	0	0										No			
			Zingiber officinale Roscoe	Yes		0	0	0	0	0	0										No			
					Acanthospermum hispidum DC.	0	0	205	0	0	0													
					Bergenia ligulata Engl.	175	472	2010	0	0	0							*Bergenia ligulata* Engl.	175+472+2010	0				
					Cucumis melo L.	0	0	455	0	0	0													
					Oenothera drummondii Hook.	0	0	18	0	0	0													
					Oenothera laciniata Hill	0	0	570	0	0	0													
					Tribulus terrestris L.	14144	9517	724	0	0	0							*Tribulus terrestris* L.	14144+9571+724	0				
17	Tablets	6	*Saraca indica* L.	Yes		0	0	0	0	0	0	4	1	3	0	3	4				No	2	1	20 %
			Asparagus racemosus Willd.*	Yes		0	0	0	0	0	0										No			
			Centella asiatica (L.) Urb.	Yes		0	0	0	0	20	12							*Centella asiatica* (L.) Urb.	0	0+20+12	Yes			
			Glycyrrhiza glabra L.	Yes		0	0	0	0	0	0										No			
			Sida cordifolia L.	Yes		0	0	0	0	0	0										No			
			Terminalia chebula Retz.	Yes		0	0	0	0	0	0										No			
						0	0	0	0	0	0													
					Althaea officinalis L.	2632	0	0	0	0	0													
					Draba nivalis Lilj.	5060	0	0	0	0	0													
					Drymocallis rupestris (L.) Soj k	80	819	0	0	0	0							*Drymocallis rupestris* (L.) Soj k	80+819+0	0				
					Luffa echinata Roxb.	0	0	0	0	132	0													
					Nelumbo nucifera Gaertn.	0	0	471	0	46	0													
					Piper longum L.	0	0	126	0	0	0													
					Senna alexandrina Mill.	0	0	0	0	0	157													
					Trianthema portulacastrum L.	0	0	313	0	0	105													
					Tribulus terrestris L.	0	0	0	0	0	27													
					Trigonella foenum-graecum L.	498	0	0	0	0	0													
18	Tablets	6	*Boswellia serrata* Roxb. ex Colebr.*	Genus is represented		0	0	0	0	0	0	2	3	2	0	4	2				No	3	0	0 %
			Alpinia galanga (L.) Willd.*	Yes		0	0	0	0	0	0										No			
			Commiphora wightii (Arn.) Bhandari*	Yes		0	0	0	0	0	0										No			
			Glycyrrhiza glabra L.*	Yes		0	0	0	0	0	0										No			
			Tinospora cordifolia (Willd.) Miers	Yes		0	0	0	0	0	0										No			
			Tribulus terrestris L.	Yes		0	0	0	0	0	0										No			
					Althaea officinalis L.	577	1392	715	0	0	0							*Althaea officinalis* L.	577+1392+715	0				
					Cucumis sativus L.	0	0	0	0	754	0													
					Cynodon dactylon (L.) Pers.	0	0	0	0	68	0													
					Draba nivalis Lilj.	12	4579	0	0	0	0							*Draba nivalis* Lilj.	12+4579+0	0				
					Fraxinus pennsylvanica Marshall	0	0	0	0	412	0													
					Melissa officinalis L.	0	0	621	0	0	0													
					Pedicularis flammea L.	0	83	0	0	0	0													
					Solanum lycopersicum L.	0	0	0	0	0	154													
					Withania somnifera (L.) Dunal	0	0	0	0	50	524							*Withania somnifera* (L.) Dunal	0	0+50+524				
19	Tablets	4	Akik pishti$			0	0	0	0	0	0	3	0	0	0	1	3					1	0	0 %
			Asparagus racemosus Willd.	Yes		0	0	0	0	0	0										No			
			Caesalpinia bonducella (L.) Fleming (= of Caesalpinia bonduc (L.) Roxb.)	Yes		0	0	0	0	0	0										No			
			Crateva nurvala Buch.-Ham	Genus is represented		0	0	0	0	0	0										No			
			Tribulus terrestris L.	Yes		0	0	0	0	0	0										No			
					Abutilon indicum (L.) Sweet	117	0	0	0	0	0													
					Drymocallis rupestris (L.) Soj k	135	0	0	0	0	0													
					Festuca gigantea (L.) Vill.	0	0	0	0	0	62													
					Momordica charantia L.	95	0	0	0	0	0													
					Salvia plebeia R.Br.	0	0	0	0	0	50													
					Thysanolaena latifolia (Roxb. ex Hornem.) Honda	0	0	0	0	3102	5703							*Thysanolaena latifolia* (Roxb. ex Hornem.) Honda	0	0+3102+5703				
23	Tablets	4	*Asteracantha longifolia Nees (= of Hygrophila auriculata (Schumach.) Heine)*	Yes		0	0	0	0	12	42	3	2	2	0	4	6	*Asteracantha longifolia Nees (= of Hygrophila auriculata (Schumach.) Heine)*	0	0+12+42	Yes	7	1	25 %
			Crocus sativus L.	Yes		0	0	0	0	0	0										No			
			Prunus amygdalus Batsch (= Prunus dulcis (Mill.) D.A.Webb)	Yes		0	0	0	0	0	0										No			
			Tribulus terrestris L.	Yes		0	0	0	0	0	0										No			
					Centaurea cyanus L.	33	0	28	0	0	0							*Centaurea cyanus* L.	33+0+28	0				
					Cucumis sativus L.	0	0	74	0	0	20													
					Fraxinus albicans Buckley	0	0	0	0	54	372							*Fraxinus albicans* Buckley	0	0+54+372				
					Hyoscyamus niger L.	37	245	0	0	0	0							*Hyoscyamus niger* L.	37+245+0	0				
					Melissa officinalis L.	5792	1689	0	0	0	0							*Melissa officinalis* L.	5792+1689+0	0				
					Salvia plebeia R.Br.	0	0	0	0	10	44							*Salvia plebeia* R.Br.	0	0+10+14				
					Senna alexandrina Mill.	0	0	0	0	0	85													
					Withania somnifera (L.) Dunal	0	0	0	0	15	116							*Withania somnifera* (L.) Dunal	0	0+15+16				
27	Capsules	1	*Gymnema sylvestre* (Retz.) R.Br. ex Sm.	Yes		64	0	12	0	0	0	11	4	10	3	5	6	*Gymnema sylvestre* (Retz.) R.Br. ex Sm.	64+0+12	0	Yes	8	1	100 %
					Ammi trifoliatum (H.C.Watson) Trel.	11	0	0	0	0	0													
					Boerhavia diffusa L.	0	0	17	0	0	0													
					Brassica oleracea L.	33	0	10	0	0	0							*Brassica oleracea* L.	33+0+10	0				
					Centella asiatica (L.) Urb.	0	0	0	0	38	0													
					Chaerophyllum reflexum Aitch.	31	0	18	0	0	0							*Chaerophyllum reflexum* Aitch.	31+0+18	0				
					Chamaecrista olesiphylla (Vogel) H.S.Irwin & Barneby	0	0	0	0	64	0													
					Coccinia sessilifolia (Sond.) Cogn.	52	17	0	0	0	0							*Coccinia sessilifolia* (Sond.) Cogn.	52+19+0	0				
					Convolvulus prostratus Forssk.	0	0	10	0	0	0													
					Coriandrum sativum L.	41	0	0	0	0	90													
					Cuminum cyminum L.	0	0	0	0	39	181							*Cuminum cyminum* L.	0	0+39+181				
					Cynanchum staubii Bosser	0	0	0	0	0	56													
					Fagopyrum esculentum Moench	25	0	0	0	0	0													
					Foeniculum vulgare Mill.	143	177	90	0	0	0							*Foeniculum vulgare* Mill.	143+177+90	0				
					Ipomoea parasitica (Kunth) G. Don	3695	572	10367	332	439	1885							*Ipomoea parasitica* (Kunth) G. Don	3695+572+10367	322+439+1885				
					Momordica charantia L.	15	0	0	0	0	0													
					Phyllanthus emblica L.	0	0	18	0	0	0													
					Senna alexandrina Mill.	634	1168	573	831	72	1206							*Senna alexandrina* Mill.	634+1168+573	831+72+1206				
					Terminalia bellirica (Gaertn.) Roxb.	0	0	12	0	0	0													
					Trachyspermum ammi (L.) Sprague	0	0	0	0	0	10													
					Withania somnifera (L.) Dunal	0	0	0	147	0	0													
30	Capsules	3	*Emblica officinalis Gaertn. (= of Phyllanthus emblica L.)*	Yes		0	0	0	0	0	0	1	2	1	0	0	0				No	1	1	33 %
			Terminalia bellirica (Gaertn.) Roxb.	Yes		2580	1659	0	0	0	0							*Terminalia bellirica* (Gaertn.) Roxb.	2580+1659+0	0	Yes			
			Terminalia chebula Retz.	Yes		0	0	0	0	0	0										No			
					Althaea officinalis	0	0	10418	0	0	0													
					Brassica oleracea	0	4487	0	0	0	0													
31	Tablets	10	*Ocimum tenuiflorum* L.	Yes		0	0	0	152	0	24	7	5	7	1	0	1	*Ocimum tenuiflorum* L.	0	152+0+24	Yes	7	5	50 %
			Cinnamomum zeylanicum Blume (= Cinnamomum verum J.Presl)	Yes		0	0	0	0	0	0										No			
			Curcuma longa L.	Yes		0	0	0	0	0	0										No			
			Emblica officinalis Gaertn. (= of Phyllanthus emblica L.)	Yes		0	0	0	0	0	0										No			
			Glycyrrhiza glabra L.	Yes		39	37	128	0	0	0							*Glycyrrhiza glabra* L.	39+37+128	0	Yes			
			Piper longum L.	Yes		358	1336	2185	0	0	0							*Piper longum* L.	358+1336+2185	0	Yes			
			Piper nigrum L.	Yes		117	0	15	0	0	0							*Piper nigrum* L.	117+0+15	0	Yes			
			Terminalia bellirica (Gaertn.) Roxb.	Yes		133	907	1121	0	0	0							*Terminalia bellirica* (Gaertn.) Roxb.	133+907+1121	0	Yes			
			Terminalia chebula Retz.	Yes		0	0	0	0	0	0										No			
			Zingiber officinale Roscoe	Yes		0	0	0	0	0	0										No			
					Convolvulus prostratus Forssk.	18	0	0	0	0	0													
					Perilla setoyensis G.Honda	13	48	10	0	0	0							*Perilla setoyensis* G.Honda	13+48+10	0				
					Sida acuta Burm.f.	11	26	0	0	0	0							*Sida acuta* Burm.f.	11+26+0	0				
					Terminalia arjuna (Roxb. ex DC.) Wight & Arn.	0	0	11	0	0	0													
					Tribulus terrestris L.	0	0	15	0	0	0													
			Carum copticum (L.) Benth. & Hook.f. ex C.B.Clarke (= Trachyspermum ammi (L.) Sprag	Yes		0	0	0	0	0	0										No			
			Corallium rubrum#			0	0	0	0	0	0										No			
			Curcuma longa L.	Yes		0	0	0	0	0	0										No			
			Hemidesmus indicus (L.) R. Br. ex Schult.	Yes		0	0	0	0	0	0										No			
			Santalum album L.	Yes		0	0	0	0	0	0										No			
			Saxifraga granulata L.	Yes		0	0	0	0	0	0										No			
			Sphaeranthus indicus L.	Yes		0	0	0	0	0	0										No			
					Alcea rosea L.	0	0	0	0	274	0													
					Berberis asiatica Roxb. ex DC.	0	0	0	0	85	0													
					Centella asiatica (L.) Urb.	0	0	0	0	666	26							*Centella asiatica* (L.) Urb.	0	0+666+26				
					Convolvulus microphyllus Sieber ex Spreng.	0	0	0	0	0	17													
					Convolvulus prostratus Forssk.	0	0	0	0	39	0													
					Corchorus olitorius L.	0	0	0	0	25	14							*Corchorus olitorius* L.	0	0+25+14				
32	Tablets	6			*Coriandrum sativum* L.	0	0	0	0	39	21	4	5	4	3	15	11	*Coriandrum sativum* L.	0	0+39+21		12	0	0 %

					Cuminum cyminum L.	0	0	0	0	148	901							*Cuminum cyminum* L.	0	0+148+901				
					Nardostachys jatamansi (D.Don) DC.	0	0	0	0	16	0													
					Nelumbo nucifera Gaertn.	1588	2344	1126	0	0	0							*Nelumbo nucifera* Gaertn.	1588+2344+1126	0				
					Piper longum L.	2120	433	2603	0	0	0							*Piper longum* L.	2120+433+2603	0				
					Piper nigrum L.	26	37	12	0	0	0							*Piper nigrum* L.	26+37+12	0				
					Pyrus calleryana Decne.	0	0	0	0	29	0													
					Rosa rugosa Thunb.	0	0	0	0	39	12							*Rosa rugosa* Thunb.	0	0+39+12				
					Senna alexandrina Mill.	0	16	40	272	76	18							*Senna alexandrina* Mill.	0+16+40	272+76+18				
					Senna auriculata (L.) Roxb.	0	0	0	0	246	313							*Senna auriculata* (L.) Roxb.	0	0+246+313				
					Sesamum indicum L.	0	0	0	0	0	25													
					Tribulus terrestris L.	10	0	0	2790	440	2059							*Tribulus terrestris* L.	0	2790+440+2059				
					Veronica plebeia R. Br.	0	120	0	0	0	0													
					Withania somnifera (L.) Dunal	0	0	0	185	44	196							*Withania somnifera* (L.) Dunal	0	185+44+196				
34	Tablets	8	*Amomum subulatum* Roxb.	Yes		0	0	0	0	0	0	10	8	4	6	8	14				No	15	5	63 %
			Cinnamomum zeylanicum Blume (= Cinnamomum verum J.Presl)	Yes		0	0	0	0	0	0										No			
			Glycyrrhiza glabra L.	Yes		358	164	143	0	0	0							*Glycyrrhiza glabra* L.	358+164+143	0	Yes			
			Iris germanica L.	ITS2 and Genus is represented		0	0	0	0	0	0										No			
			Piper longum L.	Yes		3017	1083	189	0	0	0							*Piper longum* L.	3017+1083+189	0	Yes			
			Piper nigrum L.	Yes		1095	0	80	0	0	0							*Piper nigrum* L.	1095+0+80	0	Yes			
			Terminalia arjuna (Roxb. ex DC.) Wight & Arn.	Yes		389	34	55	0	0	0							*Terminalia arjuna* (Roxb. ex DC.) Wight & Arn.	389+34+55	0	Yes			
			Zingiber officinale Roscoe	Yes		62	10	0	0	0	0							*Zingiber officinale* Roscoe	62+10+0	0	Yes			
					Boerhavia diffusa L.	0	0	0	133	28	64							*Boerhavia diffusa* L.	0	133+28+64				
					Cinnamomum cassia (L.) J.Presl	0	0	0	110	2300	115							*Cinnamomum cassia* (L.) J.Presl	0	110+2300+115				
					Convolvulus microphyllus Sieber ex Spreng.	0	0	0	0	523	248							*Convolvulus microphyllus* Sieber ex Spreng.	0	0+523+248				
					Convolvulus prostratus Forssk.	37	10	0	657	15	0							*Convolvulus prostratus* Forssk.	37+10+0	657+15+0				
					Convolvulus subhirsutus Regel & Schmalh.	0	0	0	0	0	292													
					Coriandrum sativum L.	0	0	0	0	0	28													
					Cuminum cyminum L.	0	0	0	0	17	26							*Cuminum cyminum* L.	0	0+17+26				
					Inula racemosa Hook.f.	0	0	0	176	0	114							*Inula racemosa* Hook.f.	0	176+0+114				
					Justicia adhatoda L.	0	0	0	0	0	185													
					Mentha spicata L.	0	0	0	104	0	0													
					Nyctanthes arbor-tristis L.	0	0	0	0	65	67							*Nyctanthes arbor-tristis* L.	0	0+65+67				
					Pistacia atlantica Desf.	12	0	0	0	0	0													
					Plantago major L.	0	0	0	0	0	107													
					Rosa rugosa Thunb.	0	0	0	0	0	10													
					Senna occidentalis (L.) Link	0	0	0	0	0	44													
					Sesamum indicum L.	0	0	0	0	0	69													
					Sida acuta Burm.f.	101	16	0	0	0	0							*Sida acuta* Burm.f.	101+16+0	0				
					Tribulus terrestris L.	100	17	0	1275	814	800							*Tribulus terrestris* L.	100+17+0	1275+814+800				
					Vigna radiata (L.) R.Wilczek	593	103	0	0	0	0							*Vigna radiata* (L.) R.Wilczek	593+103+0	0				
					Withania somnifera (L.) Dunal	0	0	0	0	593	0													
36	Tablets	13	*Andrographis paniculata* (Burm.f.) Nees	Yes		0	0	0	0	0	0	7	6	5	1	1	1				No	7	4	31 %
			Boswellia serrata Roxb. ex Colebr.	Yes		0	0	0	0	0	0										No			
			Carum copticum (L.) Benth. & Hook.f. ex C.B.Clarke (= Trachyspermum ammi (L.) Sprag	Yes		0	0	0	0	0	0							*Trachyspermum ammi* (L.) Sprague	0	13630+29+70	Yes			
			Curcuma longa L.	Yes		0	0	0	0	0	0										No			
			Cypraea moneta$			0	0	0	0	0	0										No			
			Emblica officinalis Gaertn. (= Phyllanthus emblica L.)	Yes		0	0	0	0	0	0										No			
			Lotus arabicus L.	Yes		0	0	0	0	0	0										No			
			Myristica fragrans Houtt.	Yes		0	0	0	0	0	0										No			
			Piper longum L.	Yes		813	2001	303	0	0	0							*Piper longum* L.	813+2001+303	0	Yes			
			Swertia chirata Buch.-Ham. ex Wall.	Yes		0	0	0	0	0	0										No			
			Terminalia bellirica (Gaertn.) Roxb.	Yes		0	0	0	0	0	0										No			
			Terminalia chebula Retz.	Yes		0	0	0	0	0	0										No			
			Trigonella foenum-graecum L.	Yes		3623	425	685	0	0	0							*Trigonella foenum-graecum* L.	3623+425+685	0	Yes			
			Zingiber officinale Roscoe	Yes		14	16	12	0	0	0							*Zingiber officinale* Roscoe	14+16+12	0	Yes			
					Olea europaea L.	315	87	53	0	0	0							*Olea europaea* L.	315+87+53	0				
					Piper nigrum L.	14	0	0	0	0	0													
					Raphanus sativus L.	24	0	10	0	0	0							*Raphanus sativus* L.	24+0+10	0				
					Trachyspermum ammi (L.) Sprague	0	29	0	13630	29	70													
					Tribulus terrestris L.	18	10	0	0	0	0							*Tribulus terrestris* L.	18+10+0	0				
40	Capsules	4	*Cissus quadrangularis* L.	Yes		0	0	0	0	51	13	3	2	2	4	6	9	*Cissus quadrangularis* L.	0	0+51+13	Yes	8	1	25 %
			Ocimum gratissimum L.	Yes		0	0	0	0	0	0										No			
			Phyllanthus emblica L.	Yes		0	0	0	0	0	0										No			
			Terminalia arjuna (Roxb. ex DC.) Wight & Arn.	Yes		0	0	0	0	0	0										No			
					Boerhavia diffusa L.	0	10	19	103	0	37							*Boerhavia diffusa* L.	0+10+19	103+0+37				
					Damrongia cyanantha Triboun	0	0	0	0	187	244							*Damrongia cyanantha* Triboun	0	0+187+244				
					Drypetes hoaensis Gagnep.	0	0	0	199	500	205							*Drypetes hoaensis* Gagnep.	0	199+500+205				
					Loxocarpus alata R.Br.	0	0	0	0	0	19													
					Ocimum basilicum L.	90	0	0	2466	138	239							*Ocimum basilicum* L.	0	2466+138+239				
					Ocimum tenuiflorum L.	0	0	0	239	70	283							*Ocimum tenuiflorum* L.	0	239+70+283				
					Perilla setoyensis G.Honda	89	11	42	0	0	0							*Perilla setoyensis* G.Honda	89+11+42	0				
					Plantago major L.	0	0	0	0	21	26							*Plantago major* L.	0	0+21+26				
					Tinospora cordifolia (Willd.) Miers	0	0	0	0	0	39													
					Vitex agnus-castus L.	14	0	0	0	0	0													
52	Powder	1	*Eclipta alba (L.) Hassk. (= Eclipta prostrata (L.) L.)*	Yes		539	0	12	1389	6616	78297	1	1	1	1	1	2	*Eclipta alba (L.) Hassk. (= Eclipta prostrata (L.) L.)*	539+0+12	1389+6616+78297	Yes	1	1	100 %
					Amaranthus viridis L.	0	0	0	0	0	12													
55	Powder	1	*Sida cordifolia* L.	Yes		0	27	20	0	0	0	6	4	3	5	1	4	*Sida cordifolia* L.	0+27+20	0	Yes	4	1	100 %
					Justicia adhatoda L.	0	12	0	0	0	11													
					Melissa officinalis L.	10	0	0	0	0	0													
					Momordica charantia L.	22	0	0	180	0	0													
					Polypogon monspeliensis (L.) Desf.	0	0	0	132	0	0													
					Sida acuta Burm.f.	3834	1621	318	0	0	16							*Sida acuta* Burm.f.	3834+1621+318	0				
					Solanum virginianum L.	0	0	0	124	0	0													
					Terminalia chebula Retz.	10	0	0	0	0	0													
					Trianthema portulacastrum L.	31	0	0	342	15	34							*Trianthema portulacastrum* L.	0	342+15+34				
					Tribulus terrestris L.	208	154	0	1216	0	117							*Tribulus terrestris* L.	439+222+0	7375+16+117				
					Vigna mungo (L.) Hepper	0	0	28	0	0	0													
58	Powder	1	*Tinospora cordifolia* (Willd.) Miers	Yes		390	47	137	0	39	39	2	1	2	1	2	3	*Tinospora cordifolia* (Willd.) Miers	390+47+137	0+39+39	Yes	2	1	100 %
					Coccinia adoensis (Hochst. ex A. Rich.) Cogn.	0	0	43	0	0	0													
					Coccinia grandis (L.) Voigt	0	0	0	128	382	78							*Coccinia grandis* (L.) Voigt	0	128+382+78				
					Vigna radiata (L.) R.Wilczek	33	0	0	0	0	0													
					Withania somnifera (L.) Dunal	0	0	0	0	0	64													
60	Capsules	1	*Terminalia arjuna* (Roxb. ex DC.) Wight & Arn.	Yes		0	0	0	0	0	0	1	0	0	0	1	2				No	1	1	0 %
					Eclipta prostrata (L.) L.	0	0	0	0	0	77													
					Humulus lupulus L.	13440	0	0	0	0	0													
					Solanum lycopersicum L.	0	0	0	0	46	74							*Solanum lycopersicum* L.	0	0+46+74				
61	Capsules	1	*Boerhaavia diffusa* L.	Yes		195	1249	0	0	0	0	7	8	6	1	2	5	*Boerhaavia diffusa* L.	195+1249+0	0	Yes	5	1	100 %
					Althaea officinalis L.	0	0	0	0	0	111													
					Baccharoides anthelmintica (L.) Moench	0	0	2769	0	0	0													
					Cucumis sativus L.	17316	0	0	0	0	0													
					Cuminum cyminum L.	0	0	0	0	64	24							*Cuminum cyminum* L.	0	0+64+24				
					Doniophyton weddellii Katinas & Stuessy	268	878	0	0	0	0							*Doniophyton weddellii* Katinas & Stuessy	268+878+0	0				
					Doniophyton weddellii Katinas & Stuessy	0	0	719	0	0	0													
					Draba nivalis Lilj.	0	4329	0	0	0	0													
					Eclipta prostrata (L.) L.	14617	0	3654	0	0	133							*Eclipta prostrata* (L.) L.	14617+0+3654	0				
					Euodia hortensis J.R. Forst. & G. Forst.	0	1407	0	0	0	0													
					Hibiscus syriacus L.	103	0	0	0	0	0													
					Hieronyma alchorneoides Allemão	0	121	0	0	0	0													
					Momordica charantia L.	0	343	0	0	0	0													
					Ocimum basilicum L.	0	17	0	0	0	0													
					Plukenetia madagascariensis Leandri	0	0	11	0	0	0													
					Salvia rosmarinus Schleid.	0	0	0	0	0	143													
					Senna alexandrina Mill.	6633	0	0	0	0	0													
					Sida acuta Burm.f.	0	0	394	0	0	0													
					Trianthema portulacastrum L.	20	4149	21378	102	821	705							*Trianthema portulacastrum* L.	20+4149+21378	102+821+705				
63	Capsules	3	*Phyllanthus emblica* L.	Yes		0	0	0	0	0	0	4	1	2	0	6	7				No	6	1	33 %
			Phyllanthus niruri L.	Yes		0	0	0	0	0	0										No			
			Tinospora cordifolia (Willd.) Miers	Yes		127	0	36	0	96	39							*Tinospora cordifolia* (Willd.) Miers	127+0+36	0+96+39	Yes			
						0	0	0	0	0	0													
					Coccinia grandis (L.) Voigt	0	0	0	0	30	36							*Coccinia grandis* (L.) Voigt	0	0+30+36				
					Echinochloa colona (L.) Link	683	30	208	0	13	379							*Echinochloa colona* (L.) Link	683+30+208	0+13+379				
					Echinochloa pyramidalis (Lam.) Hitchc. & Chase	72	0	0	0	0	0													
					Ocimum tenuiflorum L.	0	0	0	0	0	34													
					Phyllanthus maderaspatensis L.	235	0	0	0	211	326							*Phyllanthus maderaspatensis* L.	0	0+211+326				
					Phyllanthus tenellus Roxb.	0	0	0	0	34	2708							*Phyllanthus tenellus* Roxb.	0	0+34+2708				
					Trigastrotheca molluginea F.Muell.	0	0	0	0	14	25							*Trigastrotheca molluginea* F.Muell.	0	0+14+25				
66	Capsules	6	*Azadirachta indica* A.Juss.	Yes		29	17	52	0	0	22	2	2	2	1	3	4	*Azadirachta indica A.Juss.*	29+17+52	0	Yes	5	3	50 %
			Curcuma longa L.	Yes		0	0	0	0	0	0										No			
			Ocimum tenuiflorum L.	Yes		0	0	0	119	27	146							*Ocimum tenuiflorum* L.	0	119+27+146	Yes			
			Pterocarpus santalinus L.f.	Yes		0	0	0	0	0	0										No			
			Rubia cordifolia L.	Yes		0	0	0	0	0	0										No			
			Tinospora cordifolia (Willd.) Miers	Yes		0	0	0	0	15	119							*Tinospora cordifolia (Willd.) Miers*	0	0+15+119	Yes			
					Ocimum basilicum L.	0	0	0	0	770	522							*Ocimum basilicum* L.	0	0+770+522				
					Perilla setoyensis G.Honda	13	0	10	0	0	0							*Perilla setoyensis* G.Honda	13+0+10	0				
					Spondias tuberosa Arruda	0	15	0	0	0	0													
68	Capsules	1	*Glycyrrhiza glabra* L.	Yes		0	0	0	0	0	0	4	5	4	1	10	7				No	8	0	0 %
					Althaea officinalis L.	0	0	0	0	32	352							*Althaea officinalis* L.	0	0+32+352				
					Baccharoides anthelmintica (L.) Moench	0	0	0	0	16	16							*Baccharoides anthelmintica* (L.) Moench	0	0+16+16				
					Cichorium intybus L.	0	0	0	0	89	0													
					Coriandrum sativum L.	0	0	0	0	0	225													
					Dyschoriste siphonantha (Nees) Kuntze	70	110	106	0	0	0							*Dyschoriste siphonantha* (Nees) Kuntze	70+110+106	0				
					Eclipta prostrata (L.) L.	0	0	0	0	16	0													
					Gymnema sylvestre (Retz.) R.Br. ex Sm.	0	0	0	0	12	0													
					Ipomoea parasitica (Kunth) G. Don	0	0	0	0	0	188													
					Justicia adhatoda L.	126	1112	3061	0	0	0							*Justicia adhatoda* L.	126+1112+3061	0				
					Ocimum basilicum L.	0	0	0	263	170	44							*Ocimum basilicum* L.	0	263+170+44				
					Oddoniodendron normandii Aubrev.	0	137	0	0	0	0													
					Piper longum L.	61	25	18	0	0	0							*Piper longum* L.	61+25+18	0				
					Salvia plebeia R.Br.	0	0	0	0	172	371							*Salvia plebeia* R.Br.	0	0+172+371				
					Saraca asoca (Roxb.) Willd.	900	4797	9881	0	0	0							*Saraca asoca* (Roxb.) Willd.	900+4797+9881	0				
					Solanum lycopersicum L.	0	0	0	0	32	0													
					Trianthema portulacastrum L.	0	0	0	0	0	353													
					Tribulus terrestris L.	0	0	0	0	57	0													
					Withania somnifera (L.) Dunal	0	0	0	0	74	0													
69	Capsules	1	*Saraca asoca* (Roxb.) Willd.	Yes		69	716	0	0	0	0	6	12	15	4	3	5	*Saraca asoca* (Roxb.) Willd.	69+716+0	0	Yes	13	1	100 %
					Allophylus bojerianus (Cambess.) Blume	0	0	44	0	0	0													
					Althaea officinalis L.	0	449	2216	0	0	0							*Althaea officinalis* L.	0+449+2216	0				
					Baccharoides anthelmintica (L.) Moench	0	2698	205	0	0	0							*Baccharoides anthelmintica* (L.) Moench	0+2698+205	0				
					Cichorium intybus L.	105	0	0	0	0	0													
					Coriandrum sativum L.	0	45	15	13	0	16							*Coriandrum sativum* L.	0+45+15	13+0+16				
					Eclipta prostrata (L.) L.	0	0	150	0	0	0													
					Evolvulus alsinoides (L.) L.	628	77	560	0	0	0							*Evolvulus alsinoides* (L.) L.	628+77+560	0				
					Foeniculum vulgare Mill.	554	653	79	0	0	0							*Foeniculum vulgare* Mill.	554+653+79	0				
					Gossypium hirsutum L.	0	12	0	0	0	0													
					Hibiscus sabdariffa L.	0	0	0	174	0	314							*Hibiscus sabdariffa* L.	0	174+0+314				
					Ipomoea parasitica (Kunth) G. Don	91	102	134	0	0	0							*Ipomoea parasitica* (Kunth) G. Don	91+102+134	0				
					Jatropha curcas L.	0	0	30	0	0	0													
					Justicia adhatoda L.	0	0	0	9218	8457	15371							*Justicia adhatoda* L.	0	9218+8457+15371				
					Mentha arvensis L.	0	0	0	0	0	21													

					Ocimum basilicum L.	0	0	0	0	61	0													
					Ocimum tenuiflorum L.	0	0	0	172	156	0							*Ocimum tenuiflorum* L.	0	172+156+0				
					Perilla setoyensis G.Honda	0	14	0	0	0	0													
					Salvia plebeia R.Br.	0	17	68	0	0	0							*Salvia plebeia* R.Br.	0+17+68	0				
					Senna alexandrina Mill.	0	0	50	0	0	0													
					Swertia chirayita (Roxb.) Buch.-Ham. ex C.B.Clarke	0	0	11	0	0	0													
					Taraxacum officinale (L.) Weber ex F.H.Wigg.	0	129	2672	0	0	0							*Taraxacum officinale* (L.) Weber ex F.H.Wigg.	0+129+2672	0				
					Tinospora cordifolia (Willd.) Miers	0	0	10	0	0	0													
					Trianthema portulacastrum L.	234	205	47	0	0	0							*Trianthema portulacastrum* L.	234+205+47	0				
					Triticum aestivum L.	0	0	0	0	0	15													
70	Capsules	1	*Aegle marmelos* (L.) Corrêa	Yes		0	0	0	0	0	0	1	4	3	4	0	3				No	2	0	0 %
					Baccharoides anthelmintica (L.) Moench	0	39542	69783	460	0	10							*Baccharoides anthelmintica* (L.) Moench	0+39542+69783	460+0+10				
					Boerhavia coccinea Mill.	0	0	0	203	0	0													
					Cucumis sativus L.	0	0	0	100	0	0													
					Eleutherococcus sessiliflorus (Rupr. & Maxim.) S.Y.Hu	0	205	0	0	0	0													
					Hieracium fuscum Vill.	2022	0	0	0	0	0													
					Hypochaeris maculata L.	0	0	107	0	0	0													
					Lactuca sativa L.	0	0	3458	0	0	0													
					Plantago ovata Forssk.	0	0	0	15	0	213							*Plantago ovata* Forssk.	0	15+0+213				
					Swertia chirayita (Roxb.) Buch.-Ham. ex C.B.Clarke	0	304	0	0	0	0													
					Taraxacum officinale (L.) Weber ex F.H.Wigg.	0	772	0	0	0	0													
					Trianthema portulacastrum L.	0	0	0	0	0	1135													
73	Tablets	14	*Asparagus racemosus* Willd.	Yes		0	0	0	0	0	0	12	14	3	8	17	18				No	14	3	21 %
			Cassia fistula L.	Yes		0	0	0	0	0	0										No			
			Echinops echinatus Roxb.	Yes		0	0	0	0	0	0										No			
			Hordeum vulgare L.	Yes		0	0	0	0	32	27							*Hordeum vulgare* L.	0	0+32+27	Yes			
			Oryza sativa L.	Yes		169	10	0	0	0	0							*Oryza sativa* L.	169+10+0	0	Yes			
			Picrorhiza kurroa Royle ex Benth.	Yes		0	0	0	0	0	0										No			
			Premna mucronata Roxb. (= Premna mollissima Roth)	Yes		0	0	0	0	0	0										No			
			Pueraria tuberosa (Willd.) DC.	Yes		0	0	0	0	0	0										No			
			Saccharum munja Roxb. (= Saccharum bengalense Retz.)	Yes		0	0	0	0	0	0										No			
			Saccharum officinarum L.	Yes		0	0	0	0	0	0										No			
			Sida cordifolia L.	Yes		0	0	0	0	44	106							*Sida cordifolia* L.	0	0+44+106	Yes			
			Solanum indicum L.	Yes		0	0	0	0	0	0										No			
			Solanum surattense Burm. f.	Yes		0	0	0	0	0	0										No			
			Tinospora cordifolia (Willd.) Miers	Yes		0	0	0	0	0	0										No			
					Abelmoschus esculentus (L.) Moench	0	41	0	0	59	26							*Abelmoschus esculentus* (L.) Moench	0	0+59+26				
					Althaea officinalis L.	22	0	0	0	0	45													
					Arachis correntina (Burkart) Krapov. & W.C.Greg.	12	0	0	0	0	0													
					Baccharoides anthelmintica (L.) Moench	0	0	69783	0	0	0													
					Boerhavia diffusa L.	0	320	0	0	0	0													
					Cenchrus americanus (L.) Morrone	0	19	0	4420	1544	246							*Cenchrus americanus* (L.) Morrone	0	4420+1544+246				
					Citrullus lanatus (Thunb.) Matsum. & Nakai	18	0	0	0	0	0													
					Combretum nigricans Lepr. ex Guill. & Perr.	0	148	0	0	0	0													
					Convolvulus prostratus Forssk.	0	241	0	105	106	74							*Convolvulus prostratus* Forssk.	0	105+106+74				
					Coriandrum sativum L.	0	0	0	0	0	26													
					Cullen corylifolium (L.) Medik.	0	12	0	0	0	0													
					Foeniculum vulgare Mill.	352	0	0	0	0	0													
					Gossypium hirsutum L.	23	12	0	242	52	72							*Gossypium hirsutum* L.	23+12+0	242+52+72				
					Helwingia chinensis Batalin	380	0	0	0	0	0													
					Hibiscus sabdariffa L.	0	0	0	0	141	171							*Hibiscus sabdariffa* L.	0	0+141+171				
					Homalium ceylanicum (Gardner) Benth.	0	0	0	0	12	0													
					Hypochaeris maculata L.	0	0	107	0	0	0													
					Ipomoea parasitica (Kunth) G. Don	0	756	0	301	1357	28							*Ipomoea parasitica* (Kunth) G. Don	0	301+1357+28				
					Jatropha curcas L.	0	0	0	0	0	37													
					Lactuca sativa L.	0	0	3458	0	0	0													
					Mentha canadensis L.	0	0	0	0	0	59													
					Microberlinia brazzavillensis A.Chev.	14	0	0	0	0	0													
					Nuxia oppositifolia (Hochst.) Benth.	520	0	0	0	0	0													
					Ocimum basilicum L.	0	0	0	0	0	40													
					Ocimum tenuiflorum L.	0	0	0	192	12	0							*Ocimum tenuiflorum* L.	0	192+12				
					Pleuropetalum darwinii Hook. f.	0	0	0	0	12	0													
					Pseudoselago serrata (P.J. Bergius) Hilliard	138	0	0	0	0	0													
					Rauvolfia serpentina (L.) Benth. ex Kurz	0	0	0	0	0	84													
					Senna alexandrina Mill.	0	272	0	959	210	129							*Senna alexandrina* Mill.	0	959+210+129				
					Senna tora (L.) Roxb.	0	0	0	162	0	0													
					Sesamum indicum L.	0	0	0	0	52	40							*Sesamum indicum* L.	0	0+52+40				
					Silene uralensis (Rupr.) Bocquet	13	0	0	0	0	0													
					Sorghum bicolor (L.) Moench	1198	988	0	130	14	12							*Sorghum bicolor* (L.) Moench	1198+988+0	130+14+12				
					Tamarindus indica L.	0	21	0	0	0	0													
					Thiloa glaucocarpa (Mart.) Eichler	0	177	0	0	0	0													
					Vigna unguiculata (L.) Walp.	0	13	0	0	0	0													
					Withania somnifera (L.) Dunal	0	0	0	0	674	19							*Withania somnifera* (L.) Dunal	0	0+674+19				
					Xenostegia tridentata (L.) D.F. Austin & Staples i	0	0	0	0	55	0													
					Zea mays L.	0	0	0	0	48	0													
74	Tablets	9	*Acorus calamus* L.*	Yes		0	0	0	0	0	0	2	1	5	1	4	6				No	5	3	33 %
			Bacopa monnieri (L.) Wettst.*	Yes		0	0	0	0	0	0										No			
			Celastrus paniculatus Willd.*	Yes		20	0	110	0	20	14							*Celastrus paniculatus* Willd.	20+0+110	0+20+14	Yes			
			*Convolvulus pluricaulis Choisy (= Convolvulus prostratus Forssk.)**	Yes		0	0	33005	1192	4808	3937							*Convolvulus pluricaulis Choisy (= Convolvulus prostratus Forssk.)*	0	1192+4808+3937	Yes			
			Corallium rubrum$			0	0	0	0	0	0													
			Foeniculum vulgare Mill.	Yes		0	0	0	0	0	0										No			
			Lavandula stoechas L.	Yes		0	0	0	0	0	0										No			
			Mytillus margaretiferous$			0	0	0	0	0	0										No			
			Nardostachys jatamansi (D.Don) DC.	Yes		0	0	0	0	0	0										No			
			Onosma bracteatum Wall	Genus is represented		0	0	0	0	0	0										No			
			Withania somnifera (L.) Dunal	Yes		0	0	0	0	116	11							*Withania somnifera* (L.) Dunal	0	0+116+11	Yes			
					Boerhavia diffusa L.	0	0	0	0	0	21													
					Centella asiatica (L.) Urb.	0	0	0	0	104	258							*Centella asiatica* (L.) Urb.	0	0+104+258				
					Cucumis melo L.	0	0	16	0	0	27													
					Eleutherococcus sessiliflorus (Rupr. & Maxim.) S.Y.Hu	0	0	62	0	0	0													
					Phyllanthus maderaspatensis L.	0	0	12	0	0	0													
					Solanum lycopersicum L.	26	205	0	0	0	0							*Solanum lycopersicum* L.	26+205+0	0				
75	Tablets	3	*Azadirachta indica* A.Juss.	Yes		0	0	49	0	73	33	0	0	5	1	2	3	*Azadirachta indica* A.Juss.	0	0+73+33	Yes	3	2	67 %
			Ocimum tenuiflorum L.	Yes		0	0	0	0	665	128							*Ocimum tenuiflorum* L.	0	0+665+128	Yes			
			Tinospora cordifolia (Willd.) Miers	Yes		0	0	0	0	0	0										No			
					Ocimum basilicum L.	0	0	0	569	0	494							*Ocimum basilicum* L.	0	569+0+494				
					Perilla setoyensis G.Honda	0	0	87	0	0	0													
					Sida acuta Burm.f.	0	0	16	0	0	0													
					Spondias tuberosa Arruda	0	0	10	0	0	0													
					Vigna mungo (L.) Hepper	0	0	12	0	0	0													
77	Powder	1	*Asparagus racemosus* Willd.	Yes		0	0	0	597	1507	353	2	3	3	1	5	8	*Asparagus racemosus* Willd.	0	597+1507+353	Yes	7	1	100 %
					Behnia reticulata (Thunb.) Didr.	1239	2765	0	0	0	0							*Behnia reticulata* (Thunb.) Didr.	1239+2765+0	0				
					Convolvulus prostratus Forssk.	0	0	0	0	0	12													
					Lawsonia inermis L.	0	16	30	0	0	26							*Lawsonia inermis* L.	0+16+30	0				
					Mentha spicata L.	0	0	0	0	0	23													
					Ocimum basilicum L.	0	0	0	0	15	59							*Ocimum basilicum* L.	0	0+15+59				
					Pseudoselago serrata (P.J. Bergius) Hilliard	0	15	0	0	0	0													
					Secale cereale L.	44	0	15	0	33	0							*Secale cereale* L.	44+0+15	0				
					Senna tora (L.) Roxb.	0	0	0	0	61	230							*Senna tora* (L.) Roxb.	0	0+61+230				
					Sida acuta Burm.f.	0	0	222	0	0	0													
					Triticum aestivum L.	0	0	0	0	30	109							*Triticum aestivum* L.	0	0+30+109				
					Withania somnifera (L.) Dunal	0	0	0	0	0	17													
79	Powder	1	*Terminalia arjuna* (Roxb. ex DC.) Wight & Arn.	Yes		0	0	0	0	0	0	2	3	1	0	3	1				No	1	0	0 %
					Althaea officinalis L.	0	727	0	0	73	0													
					Cyamopsis tetragonoloba (L.) Taub.	41	0	0	0	0	0													
					Lophocereus marginatus (DC.) S.Arias & Terrazas	0	57	0	0	0	0													
					Ocimum basilicum L.	0	0	0	0	44	273							*Ocimum basilicum* L.	0	0+44+273				
					Pisum sativum L.	0	0	14	0	0	0													
					Stenocereus chrysocarpus Sánchez-Mej.	19	0	0	0	0	0													
					Tribulus terrestris L.	0	0	0	0	31	0													
					Xeronema callistemon W.R.B.Oliv.	0	284	0	0	0	0													

Notes. "#Our quality criteria selection for plat species detection using DNA metabarcoding: a species was considered and validaded as being present within the product only if it was detected in at least 2 out of the 3 replicates. $ Non-plant ingredients. *Scientific names that indicate the use of various refined/standardized herbal substances within the product; whereas, all others indicate the use of not extracted plant material, including simply processed and comminuted plant material, within the product."
